# Oesophageal tissue screening system for assessing the retention and mucosal absorption of biologics

**DOI:** 10.1038/s41551-026-01685-9

**Published:** 2026-06-12

**Authors:** Christina Karavasili, Thomas Young, Alvin Chan, Nikhil Lal, Charmaine Jacqueline Chang, Lena Neufeld, Shriya Rangaswamy, Yuebin Huang, Jonathan Woo, Stephanie Owyang, Andrew Pettinari, Ashley Guevara, Ben Laidlaw, Niora Fabian, Alison Hayward, Giovanni Traverso

**Affiliations:** 1 David H. Koch Institute for Integrative Cancer Research and Department of Chemical Engineering, Massachusetts Institute of Technology, Cambridge, MA USA; 2 Division of Gastroenterology, Brigham and Women’s Hospital, Harvard Medical School, Boston, MA USA; 3 Department of Pharmacy, Aristotle University of Thessaloniki, Thessaloniki, Greece; 4 Department of Bioengineering, Northeastern University, Boston, MA USA; 5 Department of Mechanical Engineering, Massachusetts Institute of Technology, Cambridge, MA USA; 6 College of Computing and Data Science, Nanyang Technological University, Singapore, Singapore; 7 Lee Kong Chian School of Medicine, Nanyang Technological University, Singapore, Singapore; 8 Massachusetts Institute of Technology Media Lab, Cambridge, MA USA; 9 Department of Pharmacology and Toxicology, University of Toronto, Toronto, Ontario Canada; 10 Division of Engineering Science, University of Toronto, Toronto, Ontario Canada; 11 Division of Comparative Medicine, Massachusetts Institute of Technology, Cambridge, MA USA; 12 Broad Institute of MIT and Harvard, Cambridge, MA USA

**Keywords:** Biomedical engineering, Oesophagitis, Tissue engineering

## Abstract

Drug delivery to the oesophagus poses unique challenges, including rapid transit time due to gravity and the presence of a stratified squamous non-keratinized epithelium. Here, to rapidly identify formulations of excipients for enhanced drug delivery to the oesophagus, we developed an oesophageal tissue screening system consisting of specialized custom plates, to incorporate gravity effects, and excised oesophageal mucosa tissues. Using the screening system, we built an excipient library identifying the most effective non-toxic permeation enhancers and selected the formulation that could prolong the retention on the oesophageal mucosa. We identified an absorption enhancer that resulted in a 876-fold increase in the oesophageal transport of a model drug (4 kDa) in pig tissue. We validated this formulation in human oesophageal tissue and in vivo in pigs with the model drug and infliximab (149 kDa), demonstrating enhanced permeability. We characterized the mechanism of the approach, noting its capacity for enhanced delivery without causing cellular disruption of the oesophageal tissue. The oesophageal tissue screening system shows promise for high-throughput screening of effective oesophageal drug delivery systems.

## Abstract (web summary)

A high-throughput screening system enables the study of drug retention and absorption in the oesophagus to quickly identify effective drug formulations that overcome rapid transit time and the selective permeability of the tissue.

## Main

Oesophageal inflammatory conditions are recognized to be rising globally^1^. To address this, several interventions have been recently developed, including targeted oesophageal delivery through increased viscosity^2^, as well as the continued development of therapeutics targeting specific immunological pathways involved in the pathogenesis of these conditions, including biologic therapies such as anti-tumour necrosis factor (TNF) agents and anti-interleukin (IL)-4 receptor alpha therapies. Despite their potency and high specificity, parenteral administration of biologics remains the main route of administration and can compromise patient compliance and treatment effectiveness^2^. As a result, the development of non-invasive delivery strategies for biologic drugs has been the ‘holy grail’ of pharmaceutical research for the past few decades. Given that the oral route has long been the preferred and most convenient method for drug delivery^3^, there has been increasing interest in the development of orally delivered biologics^4^. Most formulation approaches enabling the delivery of biologic aim to enhance biologic drug delivery in the distal small bowel tract^5–10^, whereas macromolecular delivery to the upper gastrointestinal (GI) tract has been limited to the buccal^11,12^ and gastric^13,14^ mucosa, rendering the oesophagus an underinvestigated part of the GI tract in this field.

Drug targeting to the oesophagus presents several challenges for small and macromolecular therapeutics^15^. The short transit time due to the effect of gravity across the oesophageal lumen (in general, less than 14 s)^16^ and the natural clearance mechanisms of the oesophagus make it difficult to achieve prolonged drug exposure, while the oesophageal mucosa consisting of stratified squamous non-keratinized epithelium is an important barrier in drug permeation and absorption, particularly for macromolecules^15^. The existing treatment options for oesophageal disorders either are invasive, as in the case of intralesional steroid injections for oesophageal strictures^17^, or involve untargeted formulations, as used in the treatment of eosinophilic oesophagitis (EoE), a chronic and immune-mediated inflammatory disease. These treatment options include topical steroids swallowed in the form of a viscous slurry (budesonide oral suspension, EOHILIA), from a meter-dosed inhaler (fluticasone), and/or antibodies administered subcutaneously (DUPIXENT, dupilumab)^18^. Dupilumab is currently the only US Food and Drug Administration-approved biologic therapy for the symptomatic treatment of EoE and was recently approved for use in children aged 1–11 years. However, it is associated with side effects, including injection site reaction, conjunctivitis, upper respiratory tract infections, arthralgia and facial redness^19^. Furthermore, adoption of the subcutaneous injection route may also be limited by patient acceptability and/or compliance, especially in those with needle phobia. Untargeted formulations may result in suboptimal clinical outcomes, negatively affecting patient quality of life and further contributing to increasing healthcare costs. These concerns are particularly relevant for EoE, which has emerged as a common cause of upper GI tract morbidity over the past two decades. EoE is most commonly characterized by oesophageal damage (including stricturing disease), dysphagia, food impaction, heartburn or chest pain^20^ and is prevalent among both children and adults, with the highest incidence in patients aged 35–44 years^1^.

So far, research efforts have focused on increasing drug retention in the oesophagus by developing medical devices (stents^21–24^ and films^25^), mucoadhesive^26–28^ dosage forms or dosage forms that prolong the salivary concentrations of the drug^29^. The need to increase drug residency at the site of action is crucial, but it is equally important to simultaneously enhance drug mucosal uptake without compromising tissue integrity. Nevertheless, this latter aspect has not yet been systematically explored, particularly for macromolecular therapeutics.

**Fig. 1:**
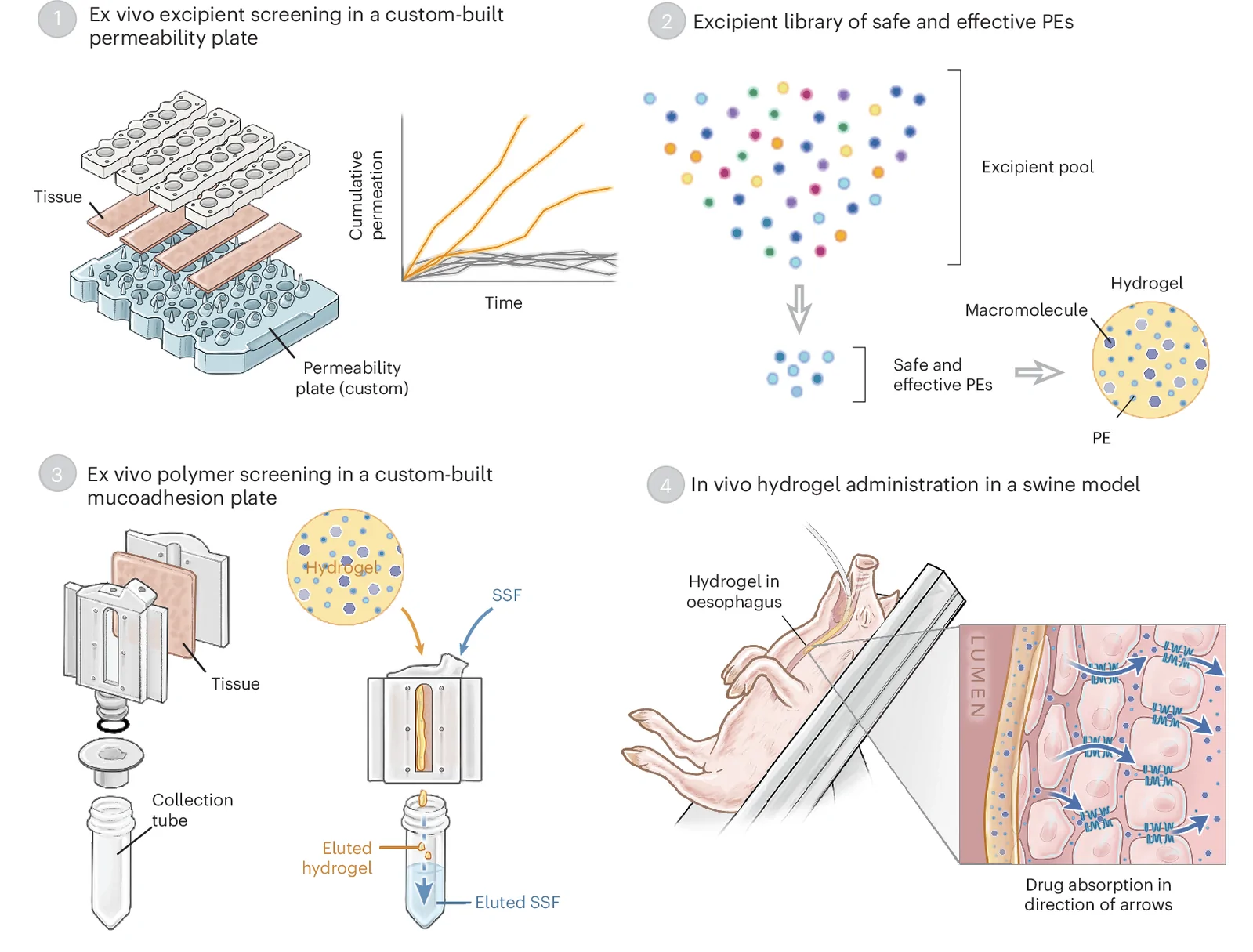
Schematic illustration of the OTiS workflow for developing the oesophageal-targeted dosage form for biologic administration. (1) We first identified excipients that increased macromolecular permeability through an ex vivo excipient screening study using a custom-built permeability plate. (2) From the generated excipient library, we identified PEs that were both effective and non-toxic to oesophageal mucosa. (3) We selected hydrogels that can prolong retention on the oesophagus based on an ex vivo mucoadhesion screening of polymers using a custom-built vertical plate. The plate is suitable for adhesion and permeability studies under the effect of gravity and the clearance effect of SSF. (4) The best-performing PEs and the hydrogel showing the longest retention on oesophageal mucosa were coformulated, and the efficacy of the system was tested in vivo in a large animal model. The hydrogel was administered endoscopically to the oesophagus of the swine secured at a semi-orthostatic position. Formulation retention and mucosal uptake of infliximab, a chimeric anti-TNF IgG1 monoclonal antibody, were monitored for up to 4 h post-administration. SSF, simulated salivary fluid.

Here, we describe the development of an oesophageal tissue screening (OTiS) platform to support the development of dosage forms that achieve prolonged retention in the oesophagus and increased mucosal uptake of macromolecular compounds. A schematic illustrating the OTiS workflow for developing the oesophageal-targeted dosage forms for biologic administration is shown in Fig. 1 involving the development of (1) high-throughput interrogation platforms, (2) rational design of a formulation library for enhancement of macromolecular therapeutics, and (3) a gravity-sensitive drug formulation evaluation system. Initially, we developed methods for the interrogation of oesophageal mucosa with respect to drug transport in a higher-throughput setting. We applied this system and identified a binary mixture (BM) of bile salts that can greatly increase macromolecular permeability through an ex vivo permeability screening of different excipient classes. After histologically confirming their compatibility with the oesophageal mucosa, we then formulated the BM of permeation enhancers (PEs) to a viscous and mucoadhesive hydrogel based on i-carrageenan. We selected a viscous hydrogel formulation because thickened fluids are recommended for managing dysphagia, a common condition in patients with EoE^30^. We tested the hydrogel’s efficacy in enhancing macromolecular permeability ex vivo across the human oesophagus under the influence of gravitational forces and simulated saliva flow. Finally, we assessed the translational potential of the developed formulation for eosinophilic esophagitis by evaluating formulation retention and infliximab mucosal uptake following in vivo administration to the oesophagus of a large animal model in a semi-orthostatic position.

## Results

### Fabrication and validation of the OTiS permeability system

**Fig. 2:**
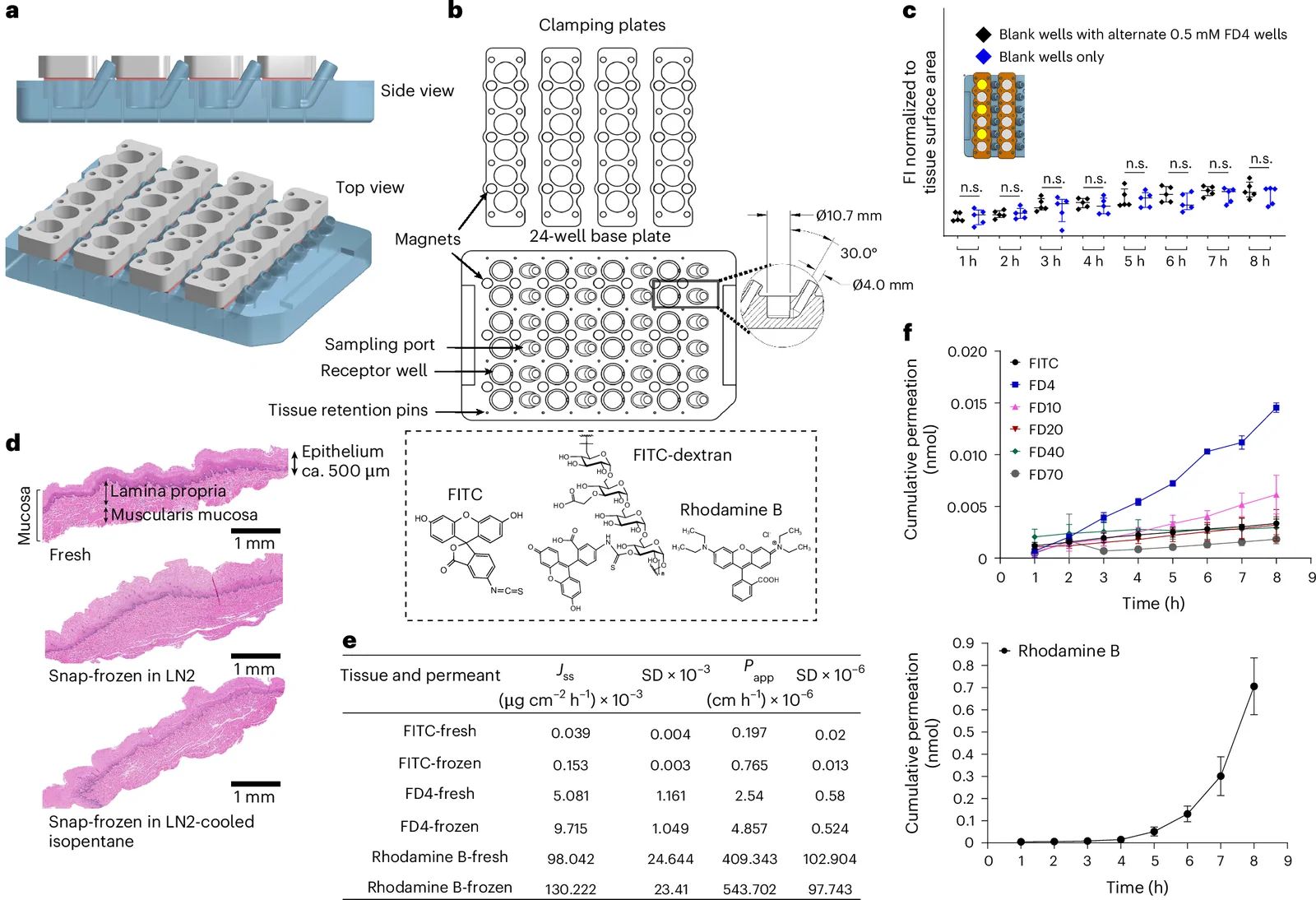
Fabrication and validation of the ex vivo permeability experimental set up. **a**, Side and top view of the horizontal permeability plate consisting of a 24-well base plate, a set of 4 compression plates, and the optional use of pins to retain the tissue laterally. **b**, The tissue plate consists of 24 acceptor wells with a well volume of 1.03 ml and an equal number of sampling ports designed to provide a 15° access angle. Aluminium alloy clamping plates are guided into place on top of the tissue by using magnetic force to form a series of donor wells. **c**, Watertight seal of the tissue between the compression and base plate was validated to confirm that no leakage would occur between adjacent wells. Data are shown as individual data points. The central horizontal line indicates the median, and the vertical bars represent the interquartile range (25th–75th percentiles). Each data point represents one independent biological replicate (*n* = 5 ± s.d., 1 h: *P* = 0.9303, 2 h: *P* = 0.7628, 3 h: *P* = 0.5455, 4 h: *P* = 0.5103, 5 h: *P* = 0.8421, 6 h: *P* = 0.1732, 7 h: *P* = 0.3264, 8 h: *P* = 0.3677). Statistical analysis was performed using one-way ANOVA (two-sided), followed by Tukey’s multiple-comparisons test. **d**, Representative histological section of fresh, snap frozen in liquid nitrogen (LN2), and in LN2-cooled isopentane oesophageal mucosae, used to evaluate the effect of cryopreservation processing on tissue integrity. Similar results were obtained from *n* = 2 independent biological replicates. **e**, Assessment of the effect of tissue processing on the permeability parameters (*J*_ss_, *P*_app_) of model fluorescent dyes (FITC, FD4 and Rhodamine B) across fresh and frozen oesophageal mucosa. **f**, Permeability mapping of the fresh oesophageal mucosa using model hydrophilic fluorescent dyes of increasing MWs (FITC, FD4 kDa–FD70 kDa) and a model lipophilic dye (Rhodamine B). Data are presented as mean ± s.d. of independent biological replicates (FITC: *n* = 3, FD4: *n* = 3, FD10: *n* = 4, FD20: *n* = 5, FD40: *n* = 4, FD70: *n* = 6, Rhodamine B: *n* = 5). SD, standard deviation; FI, fluorescence intensity. Source data

A schematic of the assembled device is shown in Fig. 2a. Plates were fabricated using clear resin to facilitate monitoring and removal of bubbles formed during tissue placement and assembly between the base and clamping plates. The 24 individual wells of the plate were designed to resemble the geometry of the Franz-type cells (Fig. 2b). Magnetic force was used to firmly seal the tissue strips between the upper clamping plates and the base plate. We validated the tissue–plate interaction by performing transport studies across freshly excised oesophageal mucosa using a fluorescent probe (FITC-dextran, molecular weight (MW) 4,000 Da; FD4) in alternating wells of the plate. Through this, we confirmed that no well-to-well leakage occurred throughout the duration of the ex vivo permeability study (Fig. 2c). To reduce our reliance on fresh tissue availability for our ex vivo excipient screening study and avoid time-consuming tissue culturing techniques, we aimed to determine whether cryopreservation would compromise tissue integrity. We tested two snap-freezing methods: liquid nitrogen and isopentane cooled in liquid nitrogen. We then evaluated the effect of each method on tissue morphology with histology. We found that the structure of the snap-frozen oesophageal mucosa (epithelium, lamina propria and muscularis mucosa) was well preserved with either method, compared with the fresh tissue control (Fig. 2d). We selected snap freezing in liquid nitrogen as our cryopreservation method, primarily because it does not involve the use of any organic solvents. We further validated the tissue integrity by testing the effect of cryopreservation on the permeability parameters of three fluorescent probes differing in their MWs (FITC and FD4) and lipophilicity (Rhodamine B). We observed slightly higher apparent permeability values for all three model compounds across frozen tissue, albeit within the same order of magnitude (Fig. 2e). Following confirmation of tissue integrity retention, our objective was the establish the permeability cut-off of the oesophageal mucosa. As depicted in Fig. 2f, hydrophilic compounds (FITC-dextrans) larger than 10 kDa did not penetrate the tissue, whereas the highly lipophilic Rhodamine B exhibited substantially higher permeability across the mucosa.

### Overcoming the oesophageal mucosal barrier with a BM of PEs

**Fig. 3:**
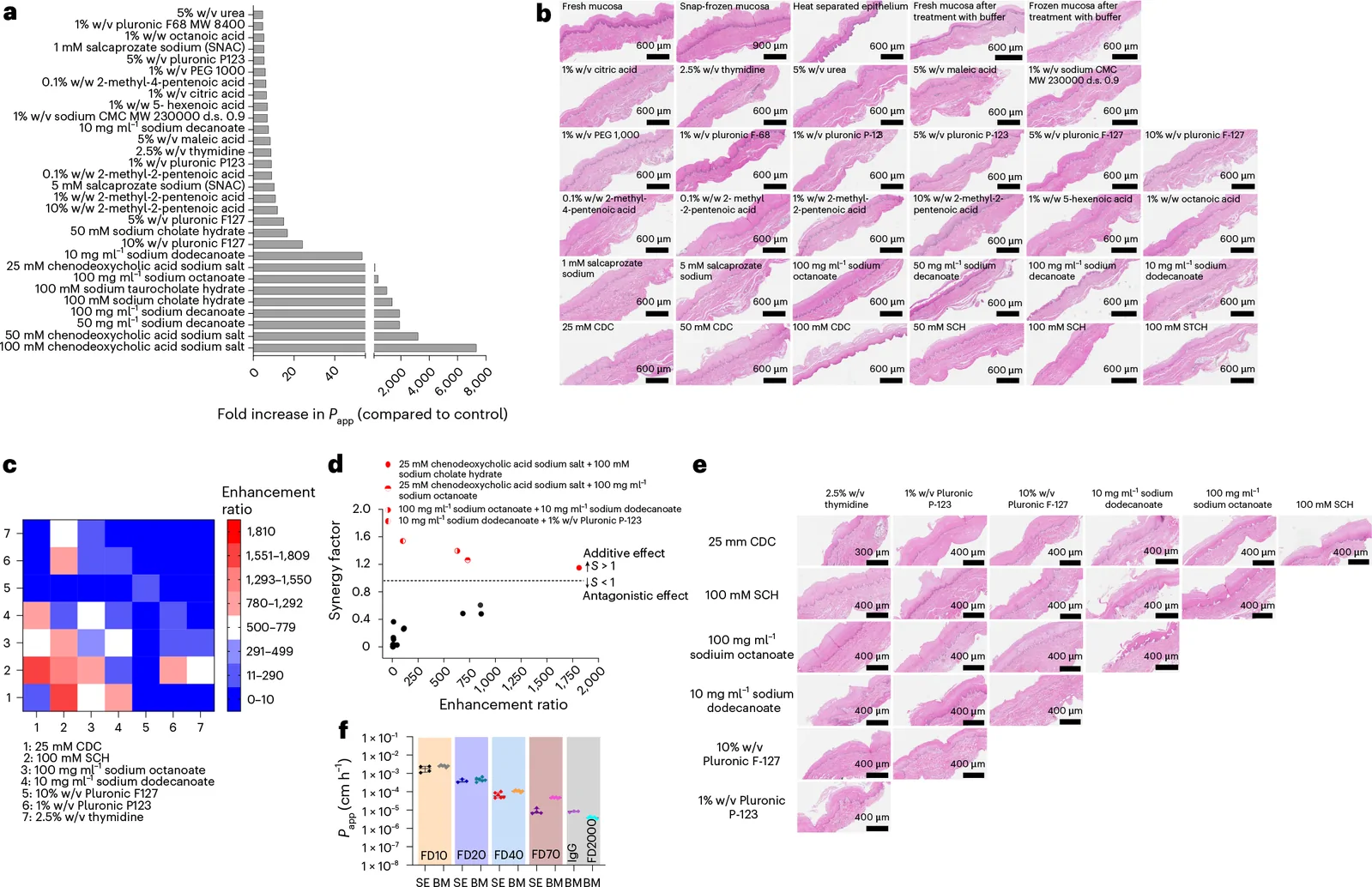
Assessment of the safety and efficacy of oesophageal PEs. **a**, Bar chart showing excipients that increased the apparent permeability of FD4 by at least a factor of 5. **b**, H&E staining of the oesophageal mucosae was conducted for tissues treated with excipients that resulted in a greater than five-fold increase in the apparent permeability of FD4. Representative histological section from one tissue sample; similar results were obtained from *n* = 2 independent biological replicates. **c**, The excipients that were confirmed to be both safe and effective as oesophageal PEs were further combined in BMs, and the potential synergy in their permeation-enhancing effect was assessed. The colour code of the heatmap indicates the enhancement ratio of the BM, with red indicating the highest and blue indicating the lowest enhancement in the permeability of FD4. **d**, The interaction effect of excipients in the BM was determined by calculating the ratio of the enhancement ratio (ER) achieved by the combination of PEs to the sum of the enhancement ratios achieved by the individual components of the combination at equivalent concentrations. Synergy (*S*) values greater than 1 indicated synergy in the permeation-enhancing effect of the excipient combination. **e**, The effect of all BMs on tissue integrity was assessed histologically. Representative histological section from one tissue sample; similar results were obtained from *n* = 2 independent biological replicates. **f**, The safest and best-performing SE (100 mM SCH) and BM (25 mM sodium CDC + 100 mM SCH) as oesophageal PEs were further evaluated for their effect on the permeability of higher-MW model compounds. The permeability of both IgG and FITC-dextran 2,000 kDa (FD2000) was tested only in the presence of the BM owing to their limited solubility in 100 mM SCH. Data are shown as individual data points. The central horizontal line indicates the median, and the vertical bars represent the interquartile range (25th–75th percentiles). Each data point represents one independent biological replicate (FD10 SE: *n* = 4, FD10 BM: *n* = 6, FD20 SE: *n* = 3, FD20 BM: *n* = 6, FD40 SE: *n* = 6, FD40 BM: *n* = 6, FD70 SE: *n* = 4, FD70 BM: *n* = 6, FD2000 BM: *n* = 5, IgG BM: *n* = 3). Source data

The large size of macromolecular compounds hinders their penetration and diffusion across mucosal barriers. As such, our goal was to identify excipients that could enhance macromolecule permeation across the oesophageal mucosal barrier. We conducted an ex vivo permeability screening of diverse classes of chemical compounds using FD4 as a model macromolecular drug (Supplementary Fig. 1). Our screening identified a variety of excipients that enhanced FD4 permeability several hundred to thousands of times (Fig. 3a). To verify that the observed permeation-enhancing effect was not the result of compromised tissue integrity, we conducted histological assessments of the oesophageal mucosae treated with the identified PEs. From this, we determined that some of the most potent PEs (100 mM chenodeoxycholic acid sodium salt (CDC), 50–100 mg ml^−1^ sodium decanoate) reduced the mucosal barrier integrity (Fig. 3b). Nevertheless, we were able to identify PEs that were both effective and non-toxic, such as 25 mM CDC, 100 mg ml^−1^ sodium octanoate and 100 mM sodium cholate hydrate (SCH), which induced a 180-fold, a 394-fold and a 1,390-fold increase in the apparent permeability (*P*_app_) of FD4, respectively.

We then analysed the impact of BMs of the most effective and safe PEs on FD4 permeability ex vivo (Fig. 3c). Our findings demonstrated that specific combinations of excipients had an additive effect on FD4 permeability compared with individual excipients alone (Fig. 3d). In particular, the BM of 25 mM CDC and 100 mM SCH had the strongest synergistic effect (*S* > 1), resulting in a 1,806-fold increase in the *P*_app_ of FD4. Importantly, this enhancement did not compromise tissue integrity, as confirmed by histology (Fig. 3e). To test the potency of the best PEs identified, either as a single excipient (SE: 100 mM SCH) or in a BM (25 mM CDC and 100 mM SCH), we evaluated their effect on enabling oesophageal permeability of model compounds with MWs ranging up to 2,000 kDa. Having already demonstrated that compounds larger than 10 kDa do not permeate the oesophageal mucosa, the observed *P*_app_ values for all MWs tested were unexpectedly high and were found to be 2.53 × 10^−3^ ± 2.91 × 10^−4^ cm h^−1^ for FD10 (BM), 4.65 × 10^−4^ ± 1.15 × 10^−4 ^cm h^−1^ for FD20 (BM), 1.08 × 10^−4^ ± 1.21 × 10^−5^ cm h^−1^ for FD40 (BM), 4.76 × 10^−5^ ± 3.25 × 10^−6^ cm h^−1^ for F70 (BM), 8.27 × 10^−6^ ± 6.81 × 10^−7^ cm h^−1^ for IgG (SE) and 3.86 × 10^−6^ ± 4.48 × 10^−7^ cm h^−1^ for FD2000 (SE), showing a trend towards decreasing permeability with increasing MW. The BM of PEs was found to be more effective in enhancing permeability compared with the single PE alone, prompting its selection for further formulation development (Fig. 3f).

### ML explanations for the permeability-enhancing properties of the excipients

We used a machine learning (ML) explanation approach to elucidate how the properties of a particular compound influence its capability to enhance permeability. First, we extracted the physiochemical properties of each compound and then trained a ML model to predict the permeability enhancement values of compounds with their physiochemical properties as input. Using a ML explanation method, TreeExplainer^31^, we extracted the SHapley Additive exPlanations (SHAP) values^32^ for each property. SHAP values are scalar values where a positive value indicates that the property contributes positively to the model output (such as predicted permeability enhancement), with the contribution proportionate to the magnitude of the SHAP value. Conversely, a negative SHAP value implies that the input property lowers the predicted permeability enhancement of the compound.

**Fig. 4:**
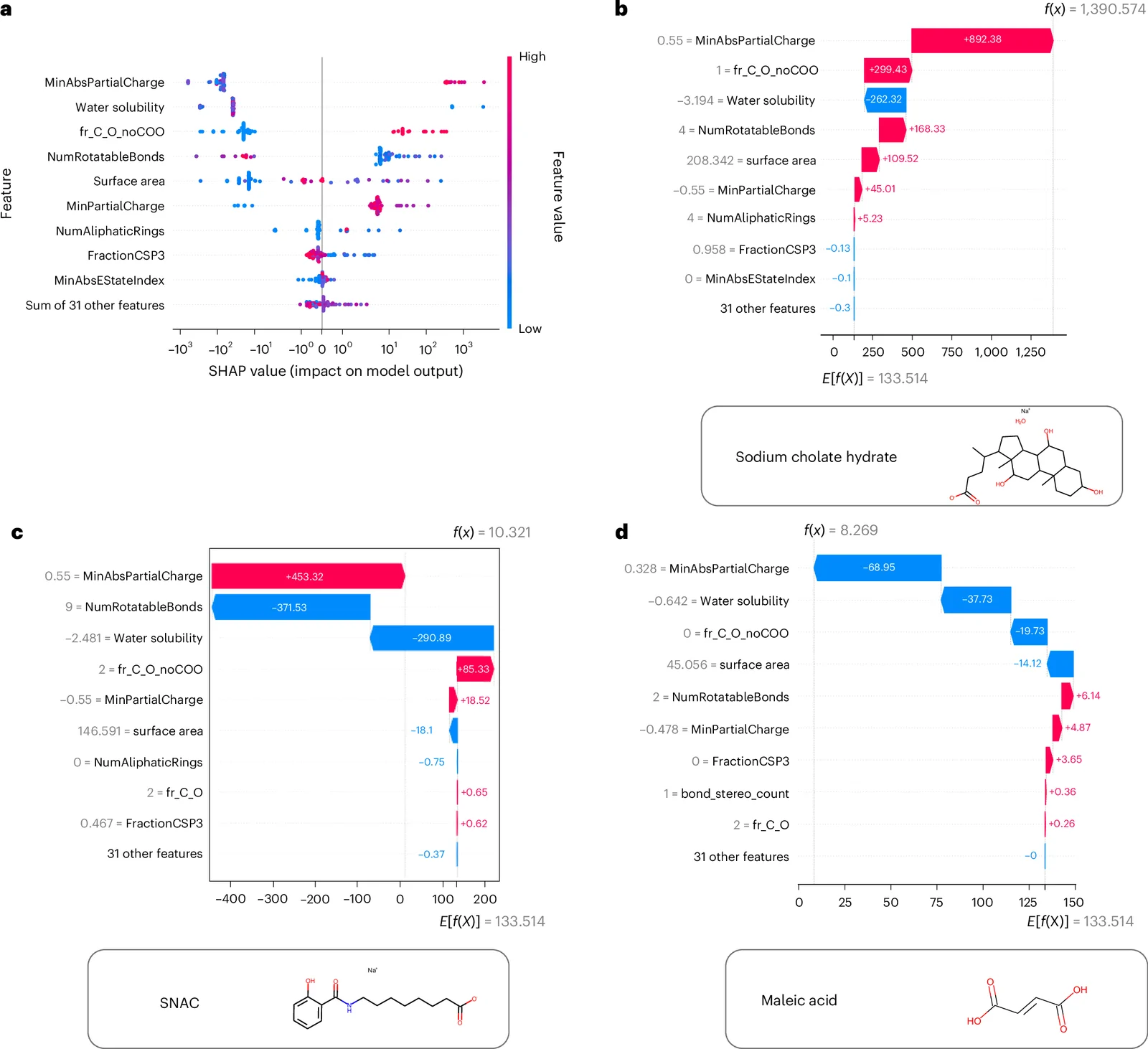
Explanations for the permeation-enhancing properties of the excipients. **a**, Global explanations showing the most influential molecular features for excipient effectiveness. **b**–**d**, Explanations for permeability-enhancing excipients show that while electron distribution is the primary driver for both sodium cholate hydrate (**b**) and SNAC (**c**), their secondary influential properties differ drastically (for example, C–O bonds positively impacting **b** versus rotatable bonds negatively impacting **c**). Panel (**d**) demonstrates that three-dimensionality is the dominant factor determining the effectiveness of maleic acid. Source data

We studied the explanations of the excipients globally (Fig. 4a) and observed that several properties have a clear relationship between property values and the effect on permeability enhancement. For properties such as ‘MinAbsPartialCharge’, ‘MinPartialCharge’ and ‘fr_C_O_noCOO’, larger values contribute positively to the predicted permeability enhancement, marked by the concentration of high-valued samples (red dots) on the right side (positive SHAP value) of the plot. Interestingly, these properties are related to the electron distribution and polarity of the compound (Supplementary Table 2) and are some of the most important factors contributing to predicted permeability enhancement. Larger values for these properties generally imply that the molecule has uneven electron distribution among its atoms. Conversely, ‘NumRotatableBonds’ and ‘FractionCSP3’ have a clear inverse effect on the predicted permeability enhancement, with low-valued samples (blue dots) concentrated on the right side of the plot. These two properties are related to the structural three-dimensionality of the excipient compound (Supplementary Table 2). Lower values for these two properties generally indicate that there is a less complex three-dimensional (3D) structure to the molecule, implying that the excipient’s three-dimensionality may not positively contribute to its permeation-enhancing properties. The list of top 40 most highly correlated features along with its Pearson correlations with respect to the permeability experimental results is presented in Supplementary Table 3. We observed that only ten of these properties have an absolute correlation value larger than 0.1.

From the SHAP explanations, we observed that different properties contribute to the enhanced permeability of different excipients. For the top-performing bile salts, such as SCH and CDC, their electron distribution (that is, ‘MinAbsPartialCharge’) has the largest contribution in enhancing permeability, marked by its large positive SHAP value (Fig. 4b and Supplementary Fig. 2). Another example is salcaprozate sodium (SNAC), which has the largest positive SHAP values from its fr_C_O_noCOO and MinAbsPartialCharge properties (Fig. 4c). From this compound, we also noted that the effects of electron distribution and three-dimensionality can conflict with each other. Here, the electron distribution properties (for example ‘fr_C_O_noCOO’ and ‘MinAbsPartialCharge’) have positive SHAP values, while its three-dimensionality (that is, NumRotatableBonds) has a negative SHAP value of the largest magnitude. In this case, its favourable electron distribution can redeem its permeation-enhancing properties from the unfavourable three-dimensionality property. Conversely, we observed that for maleic acid (Fig. 4d), favourable three-dimensionality properties (for example, NumRotatableBonds and FractionCSP3) outweigh the relatively unfavourable electron distribution (for example, MinAbsPartialCharge) property, which contributed to its permeation-enhancing properties.

### Formulation approach

The development of an oesophageal-targeted dosage form is also a challenging task because it has to overcome the effect of gravity and the natural clearance mechanisms of the oesophagus. We therefore selected a bioadhesive formulation designed to adhere to the mucosa for a sufficient length of time. This formulation, when combined with the BM of PEs, facilitates rapid drug throughput, enabling macromolecular drug absorption by oesophageal epithelial cells. The requirements we aimed to address through our formulation approach included (1) ease of manufacturing, (2) scalability, (3) ease of administration, ideally without the intervention of a medical professional, (4) cost-effectiveness, (5) safety and (6) stability of both the dosage form and the loaded macromolecular drug. We hypothesized that a thickened liquid, such as a hydrogel, could satisfy most of the aforementioned criteria. Thickened fluids are commonly used for patients with dysphagia because they extend swallowing duration. This property that could also be beneficial for an oesophageal-targeted dosage form^33^, while lyophilization can further ensure long-term stability. We investigated multiple polysaccharides of natural or semi-synthetic origin with well-established safety profiles. Rheological testing (Supplementary Fig. 3a–c) showed that most polysaccharides formed hydrogels (Supplementary Fig. 3b) with shear-thinning properties (Supplementary Fig. 3c). However, the hydrogels formed by the cellulose derivatives tended to be weaker and less viscous.

### Bioadhesive i-carrageenan hydrogels prolong retention on the oesophagus

**Fig. 5:**
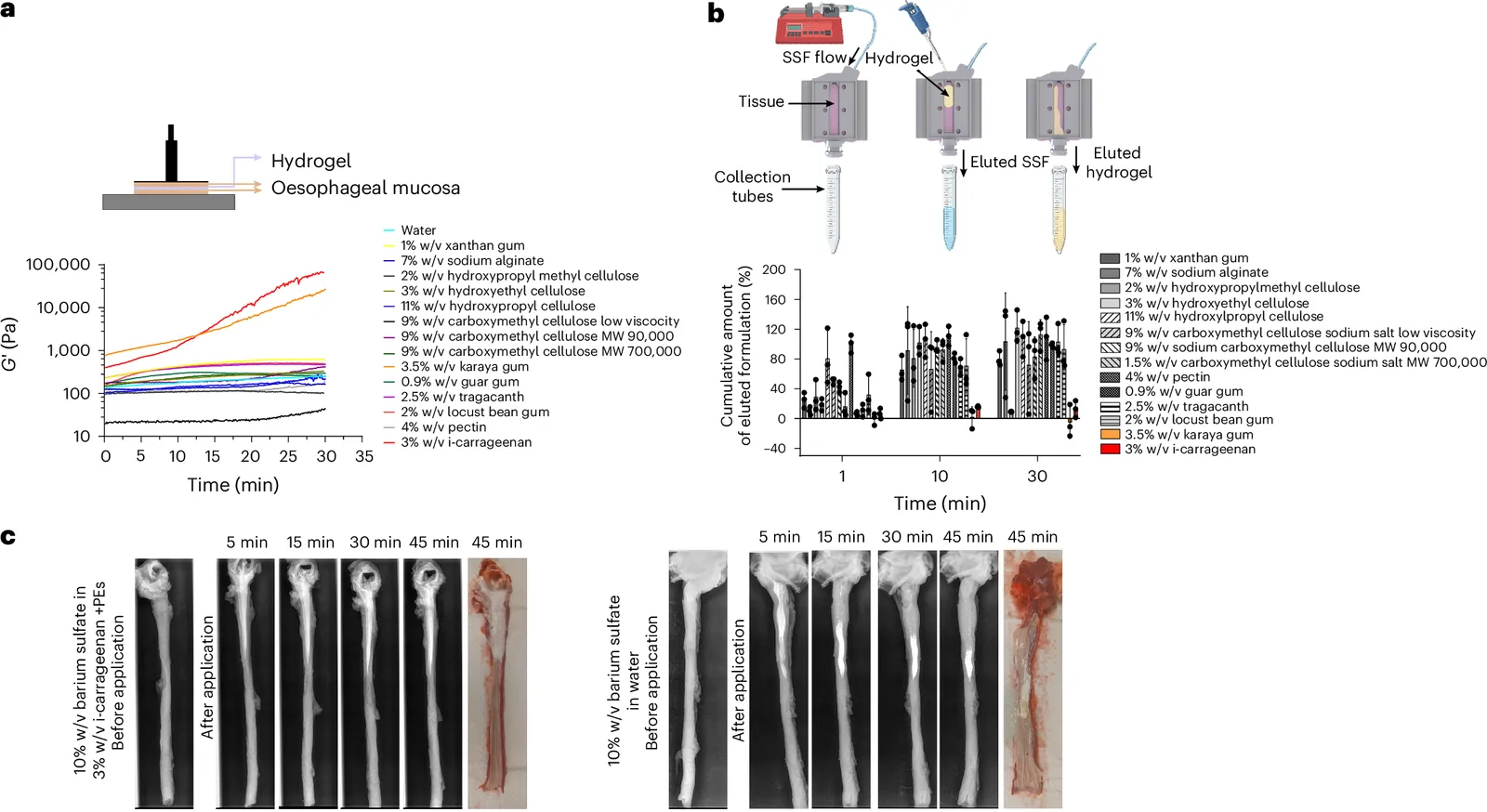
Ex vivo assessment of the bioadhesive properties of the hydrogels. **a**, Adhesive properties were tested using rheology. The hydrogels were mounted between porcine oesophageal mucosae attached to the bottom and upper plates of the rheometer. Time sweeps were performed at 1% strain and 10 rad s^−1^ for 30 min. Rheological data are shown from one representative experiment; similar results were obtained from *n* = 3 independent biological replicates. **b**, Hydrogel retention on the oesophagus was tested ex vivo using a vertical plate configuration that mimics the anatomical orientation of the oesophagus and simulates the effect of saliva clearance supplied through a pump under a constant flow rate (0.3 ml min^−1^). The cumulative amount of eluted formulation (%) was recorded for all hydrogels over 30 min. Data are presented as mean ± s.d. of *n* = 3 independent biological replicates. **c**, X-ray images of whole oesophagi were taken after applying the 3% w/v i-carrageenan hydrogel (with PEs + 10% w/v barium sulphate) to the tissue. The images evaluate tissue adhesion and retention under the flow of simulated saliva fluid (0.3 ml min^−1^), compared with the barium sulfate aqueous suspension control. Representative X-ray images from a single ex vivo porcine oesophagus experiment are shown. Illustrations in **b** created in BioRender; Traverso, G. https://biorender.com/0pz1aa1 (2026). Source data

To identify the most bioadhesive hydrogel, we investigated the rheological response of hydrogels coformulated with the BM of PEs in contact with the porcine oesophageal mucosa. Frequency sweep tests showed an increase in the storage modulus of all hydrogels when in contact with the mucosa, a pattern not observed without the mucosa (Supplementary Fig. 4). In addition, we performed time sweeps using the same experimental set-up and water as the control (Fig. 5a). Unlike frequency sweeps, time sweeps more effectively identified structural changes in the hydrogels upon contact with the mucosa. These changes indicated the progression of interactions developed between the hydrogel and the tissue over time. A steep increase in the storage modulus was observed for the 3% w/v i-carrageenan and the 3.5% w/v karaya gum hydrogels, with the increase being more pronounced for the i-carrageenan hydrogel. In contrast, the remaining hydrogels showed negligible change in their storage modulus over time.

To further substantiate these findings and assess the efficacy of our formulations, we investigated the hydrogels’ retention on the mucosal surface using a set-up that mimics the anatomical orientation of the oesophagus and simulates the effect of saliva clearance. Using the vertical plate configuration shown in Fig. 5b, we applied the hydrogels to the upper portion of the mucosal surface and recorded the elution of the formulations over time under a continuous flow of simulated salivary fluid (SSF) at a rate of 0.3 ml min^−1^. The hydrogels comprising 3% w/v i-carrageenan and 3.5% w/v karaya gum exhibited the lowest cumulative elution and demonstrated the longest retention time on the oesophageal mucosa. These results align with the conclusions drawn from the time sweep study, which corroborated the superior mucoadhesive properties of the hydrogels comprising 3% w/v i-carrageenan and 3.5% w/v karaya gum. We selected 3% w/v i-carrageenan for further studies due to the lower polymer concentration required to achieve the desired mucoadhesive effect. Radiographs of the entire oesophagus confirmed the superior adhesion and retention of the 3% w/v i-carrageenan hydrogel on the tissue, as compared with the barium sulfate aqueous suspension control (Fig. 5c).

### Bioadhesive i-carrageenan hydrogels enhance macromolecular permeability ex vivo across porcine and human oesophagus

**Fig. 6:**
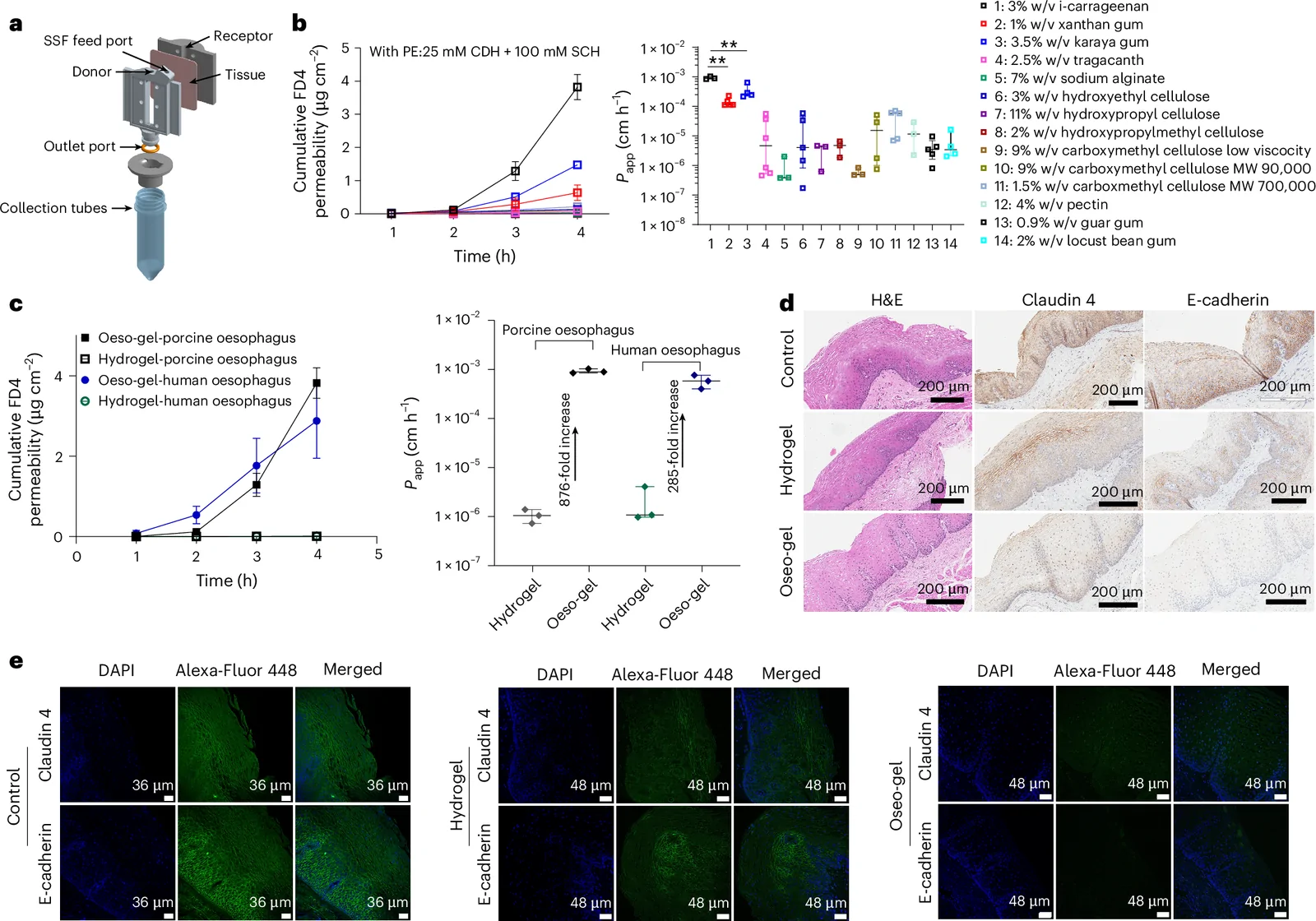
Ex vivo assessment of the permeation-enhancing properties of Oeso-gel. **a**, The ex vivo performance of the developed formulations was tested in a plate set-up that simulates the gravitational effect on drug permeability along the horizontal direction and the clearance effect of SSF. **b**, Following the application of the hydrogels containing the BM of PEs on the oesophageal mucosa, FD4 permeability was studied over 4 h. The *P*_app_ values of FD4 were calculated for the developed hydrogel formulations. All data are presented as mean ± s.d. of independent biological replicates. *P*_app_ data are shown as individual data points. The central horizontal line indicates the median, and the vertical bars represent the interquartile range (25th–75th percentiles). Each data point represents one independent biological replicate (3% w/v carrageenan: *n* = 3, 1% w/v xanthan gum: *n* = 5, 3.5% w/v karaya gum: *n* = 4, 2.5% w/v tragacanth: *n* = 6, 7% w/v sodium alginate: *n* = 3, 3% w/v hydroxyethylcellulose: *n* = 5, 11% w/v hydroxypropylcellulose: *n* = 3, 2% w/v hydroxypropylmethylcellulose: *n* = 3, 9% w/v carboxymethylcellulose low viscosity: *n* = 3, 9% w/v carboxymethylcellulose MW 90,000 g mol^−^^1^: *n* = 4, 1.5% carboxymethylcellulose MW 700,000 g mol^−1^: *n* = 5, 4% w/v pectin: *n* = 3, 0.9% w/v guar gum: *n* = 5, 2% w/v locust bean gum: *n* = 4). Statistical analysis was performed using one-way ANOVA (two-sided), followed by Tukey’s multiple-comparisons test. **P < 0.01 (3% w/v i-carrageenan versus 1% w/v xanthan gum: *P* = 0.000004972, 3% w/v i-carrageenan versus 3.5% w/v karaya gum: *P* = 0.006041). CDH, chenodeoxycholic acid. **c**, Ex vivo permeability studies were performed for the 3% w/v i-carrageenan hydrogel with (Oeso-gel) and without (hydrogel) the BM of PEs across porcine and human oesophagi from tissue donors. An 876-fold and 285-fold increase in the *P*_app_ of FD4 was observed for Oeso-gel across porcine and human oesophagi, respectively. Data are presented as mean ± s.d. of *n* = 3 independent biological replicates. *P*_app_ data are shown as individual data points. The central horizontal line indicates the median, and the vertical bars represent the interquartile range (25th–75th percentiles). Each data point represents one independent biological replicate (*n* = 3). **d**, Representative H&E staining and immunohistochemical staining of Claudin 4 and E-cadherin from one independent human oesophageal biological sample for the control and hydrogel groups and from two independent biological replicates for the Oeso-gel group. **e**, Representative immunofluorescence images of Claudin 4 and E-cadherin staining in the human oesophageal mucosae before treatment (control) and after treatment with Oeso-gel or the hydrogel. Similar staining patterns were observed in *n* = 2 independent biological replicates. Source data

We then validated the performance of the developed formulations in a set-up that would better approximate in vivo conditions. To achieve this, we used the vertical plate setup that we also employed to test adhesion (Fig. 6a), to assess the gravitational effect on drug permeability along the horizontal direction while simultaneously accounting for the clearance effect of SSF. Following the application of hydrogels containing the BM of PEs to the oesophageal mucosa, we quantified the permeability of FD4 across the tissue over 4 h. The hydrogels comprising 3% w/v i-carrageenan and 3.5% w/v karaya gum exhibited the highest cumulative FD4 permeability (Fig. 6b). This trend mirrored the formulation ranking observed during the ex vivo mucoadhesion studies (Fig. 5a). The apparent permeability coefficient of FD4 was significantly higher (*P* < 0.01) for the 3% w/v i-carrageenan (9.23 × 10^−4^ ± 0.9 × 10^−4^ cm h^−1^), compared with both the 3.5% w/v karaya gum (3.9 × 10^−4^ ± 2.16 × 10^−4 ^cm h^−1^, *P* = 0.006041) and the 1% w/v xanthan gum (1.42 × 10^−4^ ± 0.49 × 10^−4^ cm h^−1^, *P* = 0.000004972) formulations (Fig. 6b).

To elucidate the significance of the PEs on FD4 permeability across the oesophagus, we compared the permeability profiles of FD4 from the 3% w/v i-carrageenan hydrogel with (Oeso-gel) and without (hydrogel) the BM of PEs across porcine and human oesophagi from tissue donors (Fig. 6c). Oeso-gel led to a substantial enhancement in the *P*_app_ of FD4, with a remarkable 876-fold increase observed across porcine oesophagus and a 285-fold increase across human oesophagus. No statistically significant differences (*P* > 0.01) were detected in the *P*_app_ of FD4 for Oeso-gel between porcine and human oesophagi (*P* = 0.04596) (Fig. 6c). This highlights the translational potential of our formulation and validates the utility of porcine oesophagus as a suitable ex vivo model for human oesophagus. Histological examination of the human oesophageal mucosae in contact with Oeso-gel at the end of the ex vivo permeability study showed no signs of compromised tissue integrity compared with the control (Fig. 6d).

### Oesogel’s effect on paracellular transport across TJs

Transport of hydrophilic macromolecules across epithelia occurs predominantly via the paracellular route, regulated by intercellular junction proteins (for example, tight junctions (TJs) and adherens junctions (AJs))^34^. These structures act as selective barriers, limiting the diffusion of macromolecules and ions between cells. Previous studies have demonstrated that TJ proteins, such as claudins, and AJ proteins, such as E-cadherin, are strongly associated with junction barrier function and are key regulators of epithelial integrity and permeability^35^.

Building on this foundation, we investigated whether the notable increase in FD4 permeability seen upon treatment with Oesogel could be attributed to changes in the cellular distribution of these junction proteins. To this end, ex vivo human oesophageal tissues were analysed before (control) and after contact with the hydrogel formulation (with or without the PEs) using both immunohistochemistry (IHC; Fig. 6d) and immunofluorescence (Fig. 6e). In control tissues, both proteins were localized on the cell membrane at the cell–cell contacts. Claudin 4 exhibited a strong membrane localization predominantly in the suprabasal layer, while E-cadherin staining was more prominent in the basal epithelial layer, consistent with prior findings on the stratified organization of oesophageal epithelia^36,37^. Upon treatment with the hydrogel (without PEs), a mild reduction in membrane-bound Claudin 4 and E-cadherin was observed, with E-cadherin showing a more pronounced reduction. However, in tissues treated with the Oeso-gel, both Claudin 4 and E-cadherin immunoreactivity were nearly undetectable by the end of the 4-h permeability study. The same staining pattern was also observed after immunofluorescence staining of the human oesophageal tissue samples (Fig. 6e).

Interestingly, despite the apparent loss of junctional membrane staining, no evidence of cell detachment or widespread tissue damage was observed that would indicate protein integrity impairment as a result of the tissue treatment with the Oeso-gel, suggesting that the fundamental epithelial architecture remained intact. Given this lack of overt damage yet markedly enhanced permeability, we hypothesized that the Oesogel primarily affected the paracellular pathway rather than compromising overall tissue integrity. Calcium ions (Ca^2+^) are vital for preserving TJ integrity and regulating paracellular transport^38^. Such Ca^2+^-dependent modulation of TJs is well documented in the literature and has been shown to reversibly modulate the TJ barrier and increase paracellular permeability in epithelial models^38,39^. Βile salts have long been recognized for their Ca^2+^-binding properties, effectively reducing the pool of free Ca^2+^ and further facilitating the loosening of TJ barriers^34,40^. Therefore, we hypothesized that the bile salts (PEs) present in Oesogel sequester free Ca^2+^, leading to a conformational reorganization of junction complexes. Our data revealed a concentration-dependent decrease in free Ca^2+^ with increasing concentrations of CDC and SCH (PEs), culminating in nearly complete Ca^2+^ depletion at 25 mM CDC and 100 mM SCH (Supplementary Fig. 5).

These data strongly suggest that calcium depletion in the local environment, triggered by the bile salts’ high Ca^2+^ affinity, facilitates a temporary loosening of intercellular junctions. Such an effect aligns with the mechanism proposed by earlier investigations, in which chelating or reducing extracellular Ca^2+^ resulted in heightened macromolecular transport across epithelial barriers^41^.

### Oeso-gel enhances oesophageal retention and infliximab mucosal uptake in a large animal model

**Fig. 7:**
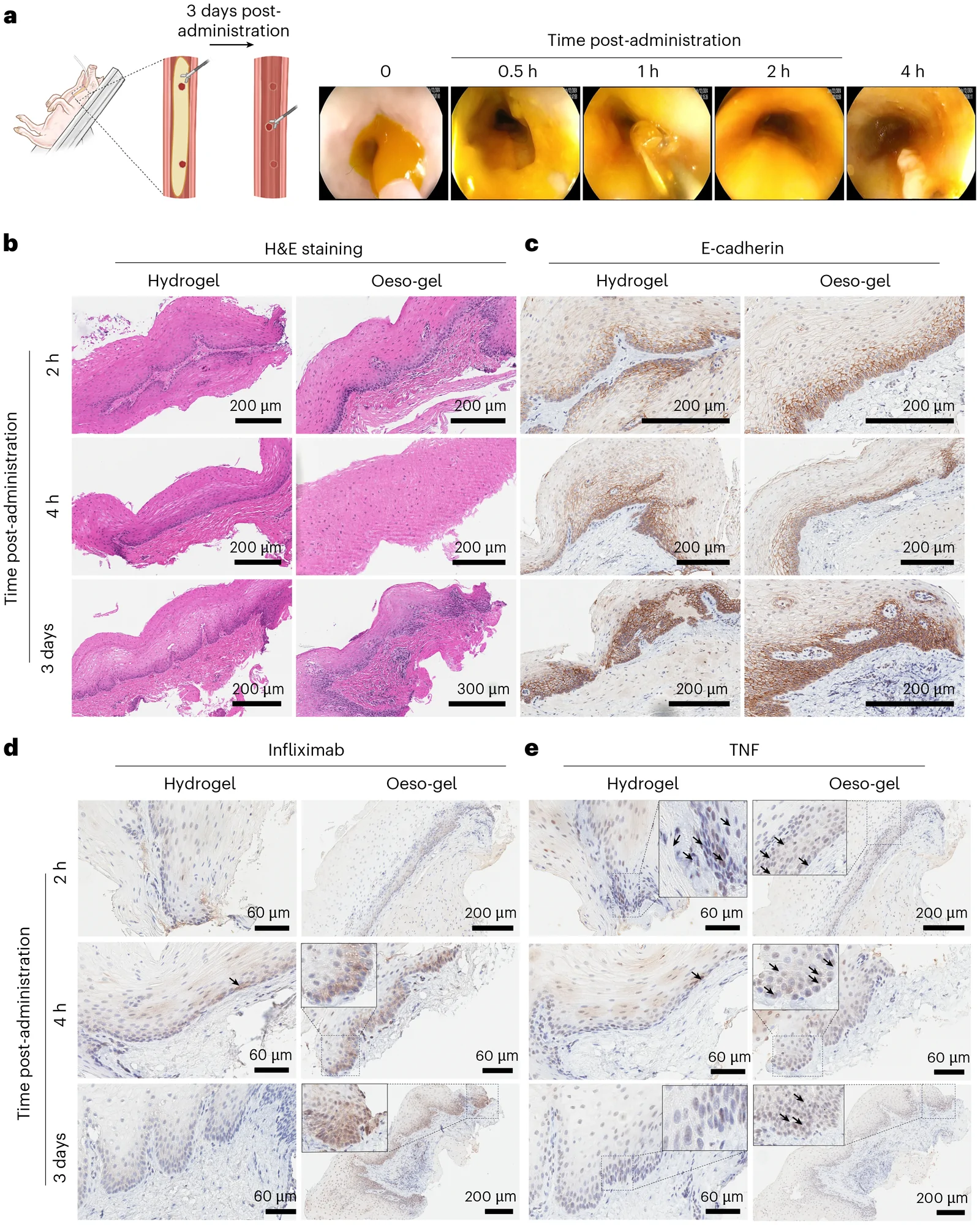
In vivo assessment of hydrogel retention and infliximab mucosal uptake after administration of Oeso-gel to the oesophagus of a large animal model. **a**, Swine were secured in a semi-orthostatic position and hydrogel retention was confirmed with endoscopy 4 h post-administration. Biopsies were collected across the flow path of Oeso-gel throughout the duration of the study and on day 3 following administration. Infliximab-loaded hydrogel (without PEs) was administered as the control. Representative endoscopic images from a single in vivo experiment are shown. **b**, Representative H&E sections of the oesophageal biopsies revealed no signs of mucosal toxicity. Similar results were obtained from *n* = 2 independent biological replicates for hydrogel and from *n* = 3 independent biological replicates for Oeso-gel. **c**, A decrease in the staining intensity of E-cadherin is evident at 2 h and 4 h post-administration of Oeso-gel, with full recovery of cell junctions observed 3 days post-administration. Similar staining patterns were observed from *n* = 2 independent biological replicates for hydrogel and from *n* = 3 independent biological replicates for Oeso-gel. **d**, Immunohistochemical staining of infliximab revealed maximum antibody uptake at 4 h post-administration, which was retained even after 3 days post-administration of Oeso-gel (as shown by the black arrows). Similar staining patterns were observed from *n* = 2 independent biological replicates for hydrogel and from *n* = 3 independent biological replicates for Oeso-gel. **e**, Immunohistochemical analysis of TNF indicated positive staining in the same mucosal regions where infliximab uptake was observed after Oeso-gel administration. Similar staining patterns were observed from *n* = 2 independent biological replicates for hydrogel and from *n* = 3 independent biological replicates for Oeso-gel. Illustrations in **a** created in BioRender. Traverso, G. https://biorender.com/lqm8v57 (2026).

We then evaluated the in vivo efficacy of Oeso-gel in a large animal model using infliximab, a potent inhibitor of the membrane-bound form of TNF that plays a crucial role in the immunopathogenesis of eosinophilic oesophagitis^42^. Endoscopic administration of Oeso-gel was performed on the oesophagus of swine secured in a semi-orthostatic position to account for the effect of gravitational forces on Oeso-gel retention. Endoscopic oesophageal mucosal biopsies were collected for up to 4 h and on day 3 post-administration (Fig. 7a). As evident from endoscopic images (Fig. 7a), Oeso-gel adhered well to the walls of the oesophagus, uniformly coating the circumference of the lumen, showing good retention even 4 h after the initiation of the study. No histological abnormalities were evident in the tissue biopsies in contact with Oeso-gel throughout the duration of the study (Fig. 7b and Supplementary Fig. 9). Immunohistochemical staining of the oesophageal biopsies for E-cadherin was performed to validate the permeation-enhancing mechanisms of Oeso-gel observed during ex vivo studies, as well as to assess the reversibility of this mechanism (Fig. 7c and Supplementary Fig. 10). A progressive decrease in E-cadherin staining intensity was observed from 2 to 4 h post-administration, consistent with a transient loosening of epithelial junctions. By day 3, staining intensity appeared to recover, suggesting reformation of cell–cell contacts and indicating the reversible nature of Oeso-gel’s action. Although a trend was observed, the changes in staining intensity probably reflect intersample variability and suggesting the need for further validation in larger cohorts. The same effect was observed for Claudin 4 (Supplementary Fig. 7). A similar staining pattern for E-cadherin was also noted on the biopsies collected from the animals to which the hydrogel (without PEs) was administered, albeit to a lesser extent compared with the Oeso-gel treated group.

Positive immunohistochemical staining for infliximab was observed in the basal layers of the oesophageal mucosa as early as 2 h following Oeso-gel administration (Fig. 7d and Supplementary Fig. 11a). Staining intensity at 4 h remained comparable to that at 2 h, indicating sustained mucosal presence of the antibody over this interval. Retention of infliximab was also evident 3 days post-administration, suggesting prolonged mucosal residence. By contrast, the hydrogel-treated group (without PEs) showed no appreciable increase in staining across the experimental timeline. The Oeso-gel group exhibited a consistent trend towards higher infliximab staining intensity compared to the hydrogel group, supporting the role of PEs in facilitating tissue uptake. No primary antibody controls were included to assess non-specific binding (Supplementary Fig. 8). Immunolocalization of the cytokine revealed that TNF staining co-localized with infliximab uptake, and low levels of TNF tissue expression were detected in the oesophageal biopsies of both groups (Fig. 7e and Supplementary Fig. 11b). A trend towards reduced TNF staining was observed in the Oeso-gel group compared with the hydrogel group at 2 h post-administration. This reduction may result from competitive binding of infliximab to TNF, limiting its detection by IHC, a phenomenon previously reported in the context of anti-TNF therapies^43,44^. While indirect, this finding may support the notion of higher infliximab uptake in the Oeso-gel group relative to hydrogel alone. No detectable infliximab levels were quantified in the serum of the animals in either group.

### Fabrication of a straw device for long-term storage and Oeso-gel administration

To identify a method for the self-administration and long-term stability of Oeso-gel, we selected a straw as an administration device to facilitate reconstitution before administration. Rehydration of the freeze-dried Oeso-gel within the straw was attempted using different static mixer designs, including a straw cleaner-type mixer, metal balls and a helix mixer. In all cases, two corrugated mesh static mixers were placed on each side of the straw to retain any undissolved material. The helix mixer yielded a suspension with suboptimal homogeneity, characterized by partially hydrated agglomerates (Supplementary Fig. 12a). Conversely, the ball mixers (Supplementary Fig. 12b) and the straw cleaner-type mixer (Supplementary Fig. 12c) demonstrated superior mixing efficiency, resulting in homogeneous hydrogels requiring minimal force during rehydration. However, achieving homogeneity required slightly more mixing time for the ball mixers compared to the straw cleaner-type mixer. The efficacy of the reconstitution process was validated by analysing the viscoelastic and mucoadhesive properties of the rehydrated Oeso-gel compared with the freshly prepared control, using carbonated and regular water as reconstitution media. All rehydrated formulations exhibited rheological properties within the same value range as the freshly prepared control. No pattern emerged indicating that either hydration medium consistently resulted in higher storage modulus values across all mixers (Supplementary Fig. 12d–f). In addition, all mixer designs, except for the helix with carbonated water, resulted in a similar amount (88–90%) of the reconstituted Oeso-gel extruded from the straw Supplementary Fig. 12g). The ex vivo retention of the reconstituted Oeso-gel on porcine oesophageal mucosa was tested using the vertical plate set-up. Similar to the control, the lowest cumulative elution and the longest retention time on the oesophageal mucosa was observed for the ball mixer, whereas the straw-cleaner type and the helix mixers showed the highest amount of eluted formulation.

## Discussion

EoE can negatively impact patients’ lives, causing symptoms such as difficulty swallowing, food impaction, chest pain and heartburn. These symptoms can lead to challenges in eating, social activities and overall quality of life. Currently the only US Food and Drug Administration-approved medications for the management of EoE are EOHILIA, a thixotropic oral suspension of a small-molecule drug (budesonide), and Dupixent, a subcutaneously administered biologic (dupilumab) that targets the IL-4Rα chain. Despite the efficacy of topical steroids, treatment discontinuation typically results in disease relapse^45^, whereas injectable therapies in chronic conditions may be associated with lower treatment compliance, especially among younger patients. In this work, we aimed to address the existing gap in non-invasive oesophageal-targeted delivery methods for biologics by developing an orally administered viscous and adhesive formulation capable of prolonging retention on the oesophageal mucosa and enhancing mucosal absorption of macromolecular therapeutics. The hydrogel formulation comprises i-carrageenan as the carrier and a BM of bile salts (chenodeoxycholic acid sodium at 25 mM salt and SCH at 100 mM) as PEs. We showed ex vivo and in live animals that the i-carrageenan-based hydrogel increases the residence time of the drug formulation at the absorptive epithelium of the oesophagus, facilitating enhanced paracellular transport of macromolecular compounds in the presence of the bile salts.

We selected a viscous liquid formulation, a well-established strategy for managing dysphagia in patients with EoE^30^. We found that i-carrageenan, which is extensively utilized in the food industry as a thickening, stabilizing and gelling agent^46^, forms viscous hydrogels that also show excellent mucoadhesive properties. We demonstrated that the polysaccharide-based hydrogel was retained on the oesophageal mucosa for at least 4 h ex vivo under the effect of gravitational forces and the clearance effect of simulated saliva flowing across the tissue. This was assessed using a custom-built plate set-up suitable for simultaneously testing formulation retention and drug permeability. We further validated our ex vivo findings in vivo using a large animal model, showing retention across the oesophagus over 4 h post-administration in swine positioned semi-orthostatically to closely mimic the anatomical orientation of human oesophagus. Previous studies on the development of oesophageal targeted dosage forms have indicated that polyvinyl alcohol-based films reside on the oesophagus of healthy volunteers for up to 15 min post-administration^47^.

Despite the fact that prolonged residency can prove beneficial in enhancing drug absorption, the paracellular transport of macromolecular compounds across mucosal barriers is known to be size-selective and regulated by the TJs between adjacent cells^34^. Through ex vivo permeability screening using custom-built permeability plates, we identified a BM of bile salts as effective oesophageal PEs for macromolecule compounds that do not compromise mucosal integrity. This was supported by a large body of evidence that has identified the ability of bile salts to enhance the paracellular transport of hydrophilic drugs^48^. Various mechanisms have been proposed to account for this permeation-enhancing effect, including bile salt micelle binding with calcium ions or bile salt micelle interaction with membrane lipids. The permeability of TJs is subject to dynamic changes influenced by a variety of external and intracellular stimuli, with calcium playing a crucial role as a key regulator of their function. Calcium-dependent cell adhesion is primarily mediated by cadherins, a family of transmembrane AJ proteins^49^, while extracellular calcium levels have also been demonstrated to influence the regulation of TJ assembly^50–52^. Low levels of calcium disrupt AJ, probably by displacing calcium ions from binding sites on the extracellular domains of E-cadherin. This displacement causes a conformational change and redistribution of E-cadherin, leading to the interruption of cell–cell adhesion. Therefore, it is possible that the BM of bile salts used in Oeso-gel enhanced macromolecular penetration through the paracellular route by binding to calcium ions. We validated this mechanism in vitro using a Ca^2+^-sensitive dye that showed complete ion complexation in the presence of the BM of bile salts. We also visualized modulation of junction proteins after treatment with Oeso-gel both ex vivo and in vivo, demonstrating in vivo that this permeation-enhancing mechanism is reversible with cell junction reformation observed three days post-Oeso-gel administration, due to extracellular Ca^2+^ replacement^53^. The reversibility of Oeso-gel’s permeation enhancing effect and the absence of mucosal toxicity are indicators of a delivery system that is both effective and safe.

Most importantly, we demonstrated for the first time enhanced mucosal uptake of infliximab co-localized with TNF following in vivo administration of Oeso-gel, at infliximab doses as low as 1 mg ml^−1^, with mucosal antibody retention observed even 3 days post-administration. Infliximab has been proven effective in the treatment of several chronic inflammatory diseases, such as ulcerative colitis, Crohn’s disease and rheumatoid arthritis by inhibiting the activity of TNF^54^. Even though TNF has been identified as a potential key mediator in the cascade of inflammatory responses in EoE^55–57^, the evidence on the clinical efficacy of infliximab against EoE is limited to a study in which three male adults with severe EoE showed no marked symptom reduction after receiving two infusions of the antibody (5 mg kg^−1^ body weight)^42^. The findings of our study, which demonstrated enhanced infliximab uptake after local application to the oesophagus at lower antibody doses compared with the parenteral ones, suggest that this alternative application mode could be considered for clinical assessment in the development of treatment options for patients with EoE. Finally, we propose an approach inspired by the concept of the medicated straw, initially proposed for drug delivery in paediatric patients^58^, to facilitate long-term storage and administration ease.

Clinical evaluation of the effect of Oeso-gel viscosity on the kinematics of the swallow response in healthy and dysphagic patients will be essential for defining the optimal viscosity range for swallow safety. In our in vivo study, the animals were sedated throughout the experimental procedure, and it is well documented that sedation can affect oesophageal motor function, making it challenging to accurately account for normal peristaltic action^59^. Furthermore, anatomical and physiological distinctions between the porcine and human oesophagus may further influence the mechanical forces acting on Oeso-gel in each species. Consequently, differences in peristaltic strength or coordination could affect how the hydrogel is transported and retained, meaning our in vivo findings in sedated pigs may not fully replicate clinical conditions in humans. It is therefore important to incorporate real-time imaging or manometric monitoring in future clinical evaluations to more accurately assess how active peristalsis affects hydrogel retention and transport under clinically relevant conditions. In addition, based on the in vivo findings of the current work, which show enhanced local uptake and retention of antibodies, future studies could also be worth assessing the systemic absorption of peptide and protein therapeutics through the oesophagus, as a less harsh alternative route for the administration of biologics.

Overall, we concluded that Oeso-gel holds great potential as a non-invasive and self-administrable oesophageal-targeted drug delivery platform for enhancing the mucosal uptake and retention of antibody therapeutics in the treatment of oesophageal disorders.

## Methods

### OTiS platform manufacturing

Vertical permeability was tested using tissue plates designed to fit the form factor typically used in a standard 48-well base plate configuration, consisting of four thick compression plates with the optional use of pins to retain the tissue laterally. The permeability plates were designed to enable high-throughput testing of vertical permeability for a variety of permeants (both fluorescent and non-fluorescent compounds), offering greater efficiency compared with conventional Franz diffusion cells. They are versatile and suitable for use with different types of tissue. The tissue plate consists of 24 acceptor wells with a well volume of 1.03 ml. The sampling ports were designed to provide a 15° access angle (diameter: 4.0 mm) for standard universal pipet tips up to 200 µl in size. Tissue strips (2 × 8 cm) were placed along four alternating columns of six wells that can be pinned down with 20-gauge steel finishing nails to mitigate the presence of folds or creases. Compression plates were placed on top of the tissue and guided into place by magnetic force to form a series of donor wells. The base plate was printed on an inkjet 3D printer (Objet260 Connex3) using the VeroClear resin, and the compression plates were provided by Protolabs’ CNC machining service using aluminium 6061 alloy. Compression plate materials explored were acrylates from Formlabs, including the ultraviolet cured Durable resin and the Clear resin. Ultimately, the compression plates were machined from 6061 aluminium alloy owing to the high stiffness and resistance to bending during plate separation. Pockets were milled into the aluminium alloy compression plates and included as 3D-printed features in the base plate for the use of nickel-plated, axially magnetized N52-grade magnets with dimensions ⅜′ diameter and ¼′ thickness. To ensure a watertight seal and mitigate well-to-well leakage, the use of 2 sets of 6 pairs of opposing magnets were used per compression plate (48 total magnets per set-up). The steel finishing nails used to support tissue retention before plate clamping were inserted from the underside of the base plate. The magnets and steel nails were retained by cyanoacrylate adhesive (Loctite 411), and to improve retention and reduce the likelihood of assembly failure, the use of an accelerator (Loctite SF 712) was used to rapidly cure the adhesive. To ensure the base plate sits flat on the bench, the base was sanded using a tabletop belt sander (Ryobi BD4601G). The magnets on the aluminium alloy compression plates were press fit using a manual drill press vise (Wilton 12800).

Formulation retention and horizontal permeability were tested in tissue plates designed to mimic the orientation of the oesophagus and simulate salivary fluid flow, providing a biorelevant environment for testing oesophageal-targeted dosage forms. The front and back plates were 3D-printed (Formlabs Form 2) using Clear resin (Formlabs). A 1/16′ clear acrylic window laser cut on a 50 W laser cutter (Universal Laser VLS 3.50) was adhered to the front plate with glue (Hernon Ultrabond Sealant 721), which was cured with an ultraviolet 365-nm flood flashlight (UVBeast V3) with an irradiance of 5,400 µW cm^−^^2^ for 1 min. The tissue was retained between the front and back plate with 2 sets of 6 axially magnetized N52 magnets (1/4′ diameter and 3/8′ in length). The magnets were secured to each plate with a cyanoacrylate adhesive (Loctite 411). To improve retention and reduce the likelihood of assembly failure, an accelerator (Loctite SF 712) was used to rapidly cure the adhesive. To further enhance the clamping pressure and form a seal with the back chamber, a set of four non-contact flow-adjustment valves made from zinc-plated steel were used to clamp the wings of the top and back plates together. The tissue was oriented with the epithelial surface facing the front plate (donor compartment), and a receptor compartment (volume 3.5 ml) was formed between the back plate and the muscularis mucosae of the tissue. SSF flow across the tissue epithelium was facilitated through a Luer lock needle hub, which had its needle removed and was glued into the top of the front plate using ultraviolet cure adhesive (Loctite 3943). The adhesive was cured with a spot ultraviolet curing flashlight (Loctite 2182210) 365 nm with an irradiance of 3,000 mW cm^−^^2^ for 30 s. A Luer lock taper fit connected to 1/16′ inner diameter (ID) silicone tubing was attached to the needle hub, and the flow rate of SSF was controlled using a syringe pump (Harvard apparatus, Harvard Bioscience). A flow rate of 0.3 ml min^−1^ was selected based on the average unstimulated whole saliva flow rate, estimated to be approximately 0.3–0.4 ml min^−1^ (ref. ^60^). The eluent was collected in a Falcon tube placed at the bottom of the vertical plate.

### Tissue preparation and storage

Porcine oesophageal tissue was procured from the local abattoir (Leman & Sons, Hilltown Pork and Blood Farm) and was processed immediately after arrival to the laboratory. The tissue was cut longitudinally, and the mucosa was separated by the underlying layers (submucosa, muscularis propria and adventitia) using blunt surgical scissors. The mucosae were rinsed with ice cold isotonic saline and were either used fresh or further processed for storage as follows. Mucosae were blotted dry and coiled on parafilm to fit in 15-ml cryogenic tubes. The tissues were snap-frozen by placing the cryogenic tubes in a thermoconductive tube rack immersed in liquid nitrogen. Snap-frozen samples were stored at −80 °C. The effect of snap-freezing on tissue integrity was assessed with histology.

Human oesophageal tissues were obtained from the National Disease Research Interchange (NDRI), a certified National Institutes of Health-funded human tissue repository. Samples originated from six deceased donors (three male and three female; five Caucasian and one Black; ages 28–62 years), as documented in the corresponding NDRI donor files. All donors were next-of-kin authorized for research (‘Research Authorization Obtained: Yes’ in all NDRI documentation). NDRI supplied fully de-identified tissues, and no identifiable private information was accessible to the researchers. Tissue specimens (10 cm) were recovered from individual donors and included the lower thoracic oesophagus and a portion of the gastroesophageal junction. Specimens were flushed with histidine tryptophan ketoglutarate or static preservation solution (SPS-1 (UW Solution)) and maintained in the same medium. Tissues were used immediately upon arrival at the laboratory.

### Excipient selection

In total, 101 chemical entities belonging to different classes of excipients (such as bile salts, fatty acids, terpenes, chitosan derivatives, non-ionic surfactants, cyclodextrins, sugars, polyethylene glycols and amino acids) were examined in the current study for their permeation-enhancing effect on oesophageal mucosa. These excipients were chosen to represent a diverse library of chemical PEs while still including conventional penetration enhancers frequently cited in the literature for use across the GI tract. A complete list of the excipients and the concentrations tested is provided in Supplementary Table 1.

### Ex vivo permeability excipient screening

Excipient screening was performed using the OTiS platform. The receptor wells were filled with phosphate-buffered saline (PBS) at pH 7.4 and maintained at 37 °C before tissue mounting on the base plate. After placement of the magnetic plates, the donor wells were filled with PBS pH 7.4 and the plate assembly was equilibrated in an incubator at 37 °C for 30 min. When frozen tissue was used, the mucosae were thawed in PBS pH 7.4 at 37 °C for 30 min before mounting on the permeability plate. After equilibration, PBS was removed from the donor wells and replaced by 100 μl of the tested solution in PBS pH 7.4. Experiments were performed at 37 °C. Samples (100 μl) were withdrawn every hour for up to 8 h and replaced by an equal volume of preheated medium.

Before the initiation of the ex vivo excipient screening assay, the watertight seal of the tissue between the magnetic and base plate was validated to confirm that no leakage would occur between adjacent wells. A 0.5 mM FD4 solution (100 μl) in PBS pH 7.4 was introduced in alternating donor wells within the same column of the permeability plate, whereas the remaining wells of the same column were filled with 100 μl of blank PBS pH 7.4 solution. Columns in which all donor wells were treated with 100 μl of blank PBS pH 7.4 solution were used as a control. The fluorescent signal of the samples withdrawn from the receptors of the wells treated with the blank PBS solution (columns with alternating FD4 treated wells and control columns) was then compared. The effect of MW and lipophilicity on tissue permeability (fresh tissue) was evaluated using fluorescein isothiocyanate dextrans (0.5 mM) of increasing MWs (4,000–70,000 Da) as the hydrophilic probes and Rhodamine B (0.5 mM) as the lipophilic probe. The impact of snap-freezing and storage at −80 °C on the barrier integrity of oesophageal mucosa was evaluated by comparing FITC, FD4 and Rhodamine B (0.5 mM) permeability across fresh and snap-frozen tissue. For the excipient screening assay, a 0.5 mM FD4 solution (100 μl) in PBS at pH 7.4 was applied to the donor wells in the presence of the excipients tested as PEs at increasing concentrations. A microplate reader (Infinite 200 PRO, Tecan) was used for the fluorescent quantification of the samples at the following excitation/emission wavelengths: 491/525 nm for FITC, 490/520 nm for FD 4–2,000 kDa and 553/627 nm for Rhodamine B. IgG from rabbit serum (Sigma-Aldrich) was quantified using the Easy-Titer Rabbit IgG Assay Kit (Thermo Scientific) following the manufacturer’s instructions.

Apparent permeability (*P*_app_, cm h^−1^) was calculated on the basis of equation (1):

1 $${P}_{\mathrm{app}}=(\Delta Q/\Delta t)(1/A{C}_{0}),$$

where ΔQ/Δt is the steady-state flux (μg cm^−^^2^ h^−1^), *A* is the surface area of the tissue (cm^2^) and *C*_0_ is the initial concentration of the permeant in the donor well (μg ml^−1^). All ex vivo permeability experiments were conducted using tissue from at least three animals.

At the conclusion of SE screening, those showing the highest permeation-enhancing effects were combined in pairs, and the effect of their interaction was calculated on the basis of equation (2)^60^:

2 $$S={\mathrm{ER}}_{{\rm{A}}+{\rm{B}}}/\left({\mathrm{ER}}_{{\rm{A}}}+{\mathrm{ER}}_{{\rm{B}}}\right),$$

where ER_A+B_ is the enhancement ratio achieved by the combination of the PEs A and B, and ER_A_ and ER_B_ are the enhancement ratios achieved by the individual components of the combination at the same concentrations. *S* values above 1 denoted synergy in the permeation-enhancing effect of the excipient combination.

### Histology

Histology was used to assess the effect of excipient treatment on the integrity of the oesophageal mucosa. Oesophageal mucosae were fixed in 10% formaldehyde buffered solution at pH 7.4 at room temperature. After 24 h, tissue specimens were placed in 70% ethanol and further processed for paraffin embedding, sectioning, and hematoxylin and eosin (H&E) staining.

### ML model training

Over 200 physicochemical features of the excipients were extracted from RDKit 2022.09.1 (‘RDKit: Open-source cheminformatics. https://www.rdkit.org’) and pkCSM^61^. We removed chemoinformatic features (for example, molecular fingerprints) to facilitate downstream interpretation. As the number of features was larger than the number of samples, we selected a subset of features to avoid overfitting by first computing the correlation between these properties of the excipients and the permeability enhancement. To obtain more meaningful explanations, the property ‘NumAliphaticCarbocycles’ was also excluded from consideration, as it tends to dominate the explanation due to the presence of multiple carbocycles in the two best-performing excipients (CDC and SCH). We then selected the top 40 most correlated features to use as input in the training data for the ML model. We used the XGBoost 1.5.0^62^, a gradient-boosted tree model, using the implementation from the Python xgboost library (https://github.com/dmlc/xgboost) to train a regressor model to predict the permeability enhancement value given the 40 input features. To generate explanations, we used the TreeExplainer algorithm from the Python SHAP (SHAP version 0.42.1) library (https://github.com/shap/shap/).

### Formulation approach

We selected naturally occurring polymers to develop a viscous hydrogel formulation for prolonged retention on oesophageal mucosa. We selected cellulose derivatives (carboxymethylcellulose, low viscosity—Sigma C5678; sodium carboxymethylcellulose, MW 90,000 g mol^−1^—Sigma 419273; carboxymethylcellulose, MW 700,000 g mol^−1^—Sigma 419338; hydroxyethyl cellulose—TCI H0418; hydroxypropyl cellulose—Alfa Aesar 43400; hydroxypropyl methyl cellulose—Sigma H7509) and polysaccharides (i-carrageenan—Sigma C1138; xanthan gum—Sigma G1253; karaya gum—Sigma G0503; tragacanth—Sigma G1128; sodium alginate—Sigma 180947; pectin—Sigma P9135; guar gum—Sigma G4129; locust bean gum—Sigma G0753). During preliminary exploration, we identified the highest concentration of each polymer that would enable hydrogel flow across a mucosal surface. Polymers were dispersed in PBS at pH 7.4 containing 25 mM CDC and 100 mM SCH and were magnetically stirred for 24 h before further testing. The concentrations tested for each polymer were the following: 9% w/v carboxymethylcellulose low viscosity, 9% w/v carboxymethylcellulose MW 90,000 g mol^−1^, 1.5% w/v carboxymethyl cellulose MW 700,000 g mol^−1^, 3% w/v hydroxyethyl cellulose, 11% w/v hydroxypropyl cellulose, 2% w/v (hydroxypropyl)methyl cellulose, 3% w/v i-carrageenan, 1% w/v xanthan gum, 3.5% w/v karaya gum, 2.5% w/v tragacanth, 7% w/v sodium alginate, 4% w/v pectin, 0.9% w/v guar gum and 2% w/v locust bean gum.

### Rheological measurements

The rheological properties of the hydrogels with and without the presence of the PEs (25 mM CDC and 100 mM SCH) were evaluated on a Discovery HR-3 Hybrid Rheometer (TA Instruments) using a parallel plate geometry (diameter 20 mm, gap 500 μm). The temperature was regulated at 25 °C by a Peltier temperature control system (TA Instruments). The linear viscoelastic region of the hydrogels was determined by strain sweeps at 10 rad s^−1^ from 0.1% to 100%. The dynamic rheological behaviour was monitored by oscillatory angular frequency sweeps in the 0.1–100 rad s^−1^ range within the linear viscoelastic region (1% strain). The flow curves of the hydrogels were obtained by shear rate sweeps between 0.1 s^−1^ and 100 s^−1^. All tests were carried out using three independent biological replicates.

### Ex vivo mucoadhesion studies

#### Rheological assessment

Tissue–formulation interactions were determined using a rotational rheometer (TA Instruments, Discovery HR-3 Hybrid Rheometer) with 8-mm parallel plates. Porcine oesophageal mucosae were cut in 8-mm samples using a biopsy punch and were attached to the top and bottom plates of the rheometer using double-sided adhesive tape. All tests were performed at 37 °C (gap 500 µm ± 250 µm). The hydrogels (100 μl) containing the PEs were applied to the surface of the oesophageal mucosa attached to the bottom plate. The top plate was then slowly lowered to contact the hydrogel, maintaining a relatively constant distance between mucosal surfaces (gap of 500 µm ± 250 µm) depending on tissue thickness. Frequency sweeps were conducted over a frequency range of 0.1–20 Hz at a constant 1% strain. Time sweeps were performed at 1% strain and 10 rad s^−1^ for 30 min. Experiments were performed using tissues from three independent biological samples.

#### Formulation retention on oesophageal mucosa

Hydrogel retention on the oesophageal mucosa was assessed using the vertical plate set-up. Porcine oesophageal mucosae were mounted between the front and back plates and 3 mL of PBS at pH 7.4 was added in the receptor compartment. SSF at pH 6.8 was introduced through a side port at 0.3 ml min^−1^, corresponding to the unstimulated or resting saliva flow rate. The hydrogels (1 ml) were applied to the top segment of the tissue and the elution of the formulation at the bottom of the plate assembly was monitored at frequent time intervals for up to 30 min. This was achieved by quantifying the weight change of the eluted medium, while accounting for the simultaneous elution of SSF. Experiments were performed using tissues from three independent biological samples. Ex vivo retention was also tested in whole porcine oesophagi with X-rays. Radiopaque barium sulfate (10% w/v) was added in 3% w/v carrageenan hydrogel (with PEs) or water. The oesophagus was secured in a vertical position and 10 ml of the hydrogel or water control was applied on the top part of the tissue. Radiographs were performed at different timepoints post-application to monitor flow speed and tissue coverage with time. SSF (0.3 ml min^−1^) was introduced through the top part of the oesophagus throughout the study. X-ray imaging of formulation retention was performed once to provide qualitative visualization of formulation positioning and retention over time.

### Ex vivo permeability formulation screening

Ex vivo permeability studies were performed using the vertical plate set-up to simulate the vertical orientation of the human oesophagus. Porcine oesophageal mucosae were mounted between the front and back plates, and 3 ml of PBS pH 7.4 was added in the receptor compartment. SSF pH 6.8 was introduced through a side port at 0.3 ml min^−1^, corresponding to the unstimulated or resting saliva flow rate. FD4 was added into the hydrogel formulations, prepared as previously described, at 2 mg g^−^1 right before study initiation. The hydrogels (1 ml) were applied on the top segment of the tissue, and samples (100 μl) were withdrawn from the receptor compartment and replaced with an equal volume of medium at 1-h intervals for up to 4 h. Experiments were performed at room temperature and repeated three times using tissue from different animals. The hydrogel formulation containing FD4 at 2 mg g^−1^ and no PEs were used as the control. The formulation that demonstrated the highest FD4 permeability was also tested using human tissue under the same experimental conditions. Biopsy samples were obtained from the human oesophageal tissue before the initiation and at the end of the ex vivo permeability study and further processed for immunohistochemical analysis of TJ proteins.

### IHC and immunofluorescent staining

Formalin-fixed paraffin-embedded human oesophageal tissues were stained for Claudin 4 at 1:10 dilution (Claudin 4 rabbit anti-human polyclonal antibody, no. PA5-32354, ThermoFisher Scientific) and E-cadherin at 1:100 (E-cadherin anti-human polyclonal antibody, no. PA5-80457, ThermoFisher Scientific, lot UK2885776). Polyclonal antibodies were diluted in Tris-buffered saline (20×), with 2% Tween 20 (ThermoFisher Scientific). Antigen retrieval was performed according to standard procedures and chromogenic staining using horseradish peroxidase (HRP)–3,3′-diaminobenzidine (DAB) detection. For immunofluorescent staining, a goat anti-rabbit IgG secondary antibody labelled with Alexa Fluor 488 (green) (catalogue number A-11008, ThermoFischer Scientific) was used at 4 μg ml^−1^. The nuclei were counterstained with DAPI (blue) (4,6-diamidino-2-phenylindole, dihydrochloride, catalogue number P36935, Invitrogen). Fluorescent samples were analysed using a confocal microscope (Nikon A1R) with a Galvano scanner and ×20 air or ×60 oil-immersion objectives.

### Bile salt complexation with calcium ions

Bile salt interaction with calcium was examined through ultraviolet–visible spectrophotometry based on a previously reported method^63^. Calcium levels were measured using the Ca^2+^-sensitive dye Pontachrome Violet SW (Sigma-Aldrich), which displays a change in absorbance after complexation with free Ca^2+^. In brief, CaCl_2_·2H_2_O was dissolved in deionized water (1,000 mg l^−1^) and a calibration curve was generated in the concentration range of 0–35 mg l^−1^ Ca^2+^ in deionized water, with 10 mg l^−1^ of Pontachrome Violet SW in each standard. To elucidate bile salt complexation with Ca^2+^, experimental samples were prepared with 10 mg l^−1^ Pontachrome Violet SW, 35 mg l^−1^ Ca^2+^, 150 mg l^−1^ NH_4_OH, and bile salts at increasing concentrations (CDC 0–35 mM, SCH 0–100 mM). Pontachrome Violet SW (10 mg l^−^1) in deionized water and Pontachrome Violet SW (10 mg l^−1^) in an aqueous solution of 150 mg l^−1^ NH_4_OH served as the controls. All measurements were taken within 20 min of solution preparation. Absorbance was measured using a microplate reader at 520, 575 and 680 nm and calcium concentration was calculated as previously reported^63^. Experiments were performed with three independent replicates.

### In vivo studies

All procedures conformed to the protocols approved by the Massachusetts Institute of Technology Committee on Animal Care under protocol number 2207000395. Female Yorkshire pigs (*Sus scrofa domestica*), 4–8 months old, were procured from the Cummings School of Veterinary Medicine at Tufts University (Grafton, MA, USA). The swine (40–103 kg) were fasted overnight with access to water ad libitum. Animals were sedated with intramuscular injection of midazolam (~0.25 mg kg^−1^) and dexmedetomidine (~0.03 mg kg^−1^), and after intubation, anaesthesia was maintained with isoflurane at 2% with oxygen. An ear catheter was inserted to allow for frequent blood sampling. Animals were then secured at a semi-orthostatic position, and the hydrogel formulations were directly administered endoscopically to the oesophagus using a tubing placed down the working channel of the endoscope to prevent formulation loss and precision over the administered volume. A 10-ml syringe was used to administer Oeso-gel (3 ml) through the tubing, advancing from the upper thoracic oesophagus to the cervical oesophagus with the endoscope. Endoscopic visualization of formulation retention in the porcine oesophagus was performed in a single animal to qualitatively assess formulation positioning and retention in vivo. The administered dose of infliximab was 1 mg ml^−1^ in Oeso-gel (*n* = 3). To match the viscosity of Oeso-gel with the control hydrogel (without PEs), we performed frequency sweeps of the i-carrageenan hydrogels without PEs at concentrations lower than 3% w/v. We found that 1.8% w/v i-carrageenan hydrogel (without PEs/control group) had similar viscosity to the Oeso-gel (Supplementary Fig. 6) and as such served as the control for the administration of infliximab (Remicade) at 1 mg ml^−1^ (*n* = 2). Blood samples were withdrawn before and at 30 min, 60 min, 120 min and 240 min post-administration and oesophageal biopsies were collected at 2 h and 4 h post-administration. Biopsies were fixed and processed for H&E staining, IHC for infliximab (human anti Infliximab, Bio-Rad HCA212, clone AbD17837, lot 168396) at 10 μg ml^−1^ using mouse anti V5-Tag:HRP (Bio-Rad MCA1360P, clone SV5-Pk1, lot 158243) as secondary antibody at 10 μg ml^−1^, TNF (anti-TNF alpha antibody, Abcam ab6671, rabbit polyclonal, lot 1053654-8) at 1:50 dilution and E-cadherin and Claudin 4, as previously described. Oesophageal mucosal biopsies were also collected 3 days post-administration and processed for IHC staining to assess recovery of the TJs and infliximab uptake. No primary antibody (infliximab) controls were included to identify false-positive staining reactions. Infliximab quantification in serum samples was performed using an enzyme-linked immunosorbent assay kit (MyBiosource.com).

Staining quantification was performed using ImageJ (v1.53). To isolate DAB-positive staining, the Color Deconvolution plugin was applied using the H DAB vector. The brown (DAB) channel was extracted and thresholded using a consistent method across all samples with the same staining and the percentage (%) area stained was calculated. Data are presented as mean ± standard error of the mean.

### Fabrication of a straw device for long-term storage and ease of administration

#### Fabrication of static mixers

Static mixers were designed using SolidWorks 2022 SP0. Mixers were sliced in PreForm software 3.28.0 and printed in Clear Resin v4 (Formlabs) using a Form 2 Formlabs printer. After printing, parts were washed for 10 min in isopropyl alcohol (IPA) using a Formlabs Form Wash and then cured at 60 °C for 30 min in a Formlabs Form Cure.

#### Hydrogel freeze drying and rehydration within the straw

The 3% w/v carrageenan hydrogel containing the BM of PEs (5 ml) was loaded into a silicone straw (1.1 cm outer diameter, Amazon) using a syringe together with a custom mixer, straw cleaner or balls (steel balls: 9587K1, plastic balls: 96455K51, McMaster Carr). The straws were frozen at −80 °C overnight and then freeze dried (Labcono Freezone 2.5 l, −50 °C, 700201000) for 48 h. After drying, straws were either immediately used or stored in a desiccator for later use. The weight of each straw was taken before freezing and after freeze drying and rehydration. Straws were reconstituted using carbonated or uncarbonated water. The formulation within each straw was crushed to fine, powdery consistency by pinching the straw. Rehydration and mixing were performed using 5-ml syringes attached to the end of the straws via custom Barb–Luer fittings. The Barb–Luer fittings were modelled and 3D-printed using the method outlined for the custom mixers. To ensure uniformity, each solution was rehydrated with an appropriate amount of water to yield a 3% w/v solution. The syringe plunger was used to pass the formulation over each mixer type and into the syringe. Each straw was mixed ten times across the mixer, rested for a 5-min wetting period, then mixed ten more times. Experiments were performed with three independent biological replicates.

#### Rheological assessment of the rehydrated hydrogels

Rheological characteristics of the rehydrated formulation were measured and compared with the freshly prepared hydrogels (controls). Frequency sweeps were performed from 0.1 rad s^−1^ to 100 rad s^−1^ at a constant 1% strain at 37 °C. The hydrogel (350 μl) was pipetted onto the 20-mm parallel plates and tested at a 500-μm gap. Experiments were performed with three independent biological replicates.

#### Formulation retention on oesophageal mucosa after rehydration

Hydrogel retention on the oesophageal mucosa was assessed immediately after rehydration using the vertical plate set-up as previously described. Experiments were performed with three independent biological replicates.

### Statistical analysis

Data analysis was performed using GraphPad Prism 10 (GraphPad Software). Data are presented as means ± standard deviation (s.d.). One-way analysis of variance (ANOVA) followed by Tukey’s post-hoc test was used for comparisons among multiple groups (*P < 0.05, **P < 0.01, ***P < 0.001).

### Reporting summary

Further information on research design is available in the Nature Portfolio Reporting Summary linked to this article.

## Supplementary information

Supplementary Information Supplementary Tables 1–3 and Figs. 1–12.

Reporting Summary

Peer Review File

Supplementary Data Source data for Supplementary Figs. 1–12.

## Source data

Source Data Figs. 2–6 Source data for Figs. 2–6.

## Data Availability

All the data supporting the results in this study are available within the Article and its Supplementary Information. Source data are provided with this paper. Additional raw histology micrographs require experiment-specific preprocessing and metadata to be interpretable. These data are available from the corresponding author upon reasonable request; requests should be made by email and will typically be fulfilled within 4 weeks.

## Code availability

The custom code used to process the raw data, train the model and generate the SHAP figures in this study is available via Zenodo at https://doi.org/10.5281/zenodo.18193963 (https://zenodo.org/records/18193963)^64^.
