# Supplementary material for: Oesophageal tissue screening system for assessing the retention and mucosal absorption of biologics
Source: Nat Biomed Eng. 2026 Jun 12;10(10):2213–29. doi: 10.1038/s41551-026-01685-9 (PMC13672101; doi:10.1038/s41551-026-01685-9)
Supplement: Supplementary file 1 [file 41551_2026_1685_MOESM1_ESM.pdf]

nature biomedical engineering

Supplementary information https://doi.org/10.1038/s41551-026-01685-9

Oesophageal tissue screening system for assessing the retention and mucosal absorption of biologics

In the format provided by the authors and unedited

Table S1. List of excipients tested in the *ex vivo* permeability screening.

Excipient	Vendor	Catalog number
DMSO	Sigma	472301
SLS	Sigma	436143
urea	Sigma	U5128
sodium cholate hydrate	U5128	C1254
sodium taurocholate hydrate	Sigma	86339
deoxycholic acid sodium salt	Sigma	D2510
chenodeoxycholic acid sodium salt	ChemCruz	sc-2883378
2-methyl-2-pentenoic acid	Sigma	W319519
2-methyl-4-pentenoic acid	Sigma	W351105
3-methylpentanoic acid	Sigma	W343706
0.1% w/w 3-methyl-4-pentenoic acid	Sigma	553115
3-methyl-2-oxopentanoic acid	Sigma	198978
2,2-Dimethylpentanoic acid (2,2-Dimethylvaleric acid)	Sigma	41740
2,2-dimethyl-4-pentenoic acid	Sigma	305138
hexanoic acid	Sigma	153745
5- hexenoic acid	Sigma	464929
2-methyl hexanoic acid	Sigma	W319104
2-hydroxy hexanoic acid	Sigma	219800
6-heptenoic acid	Sigma	358940
2-methylheptanoic acid	Sigma	W270601
ethyl heptanoate	Sigma	112364
octanoic acid	Sigma	C2875
2-octenoic acid	Sigma	5209
7-octenoic acid	Sigma	715751
4-ethyloctanoic acid	Sigma	W380008
3,7-dimethyl-6-ethyloctenoic acid	Sigma	W314220
(+-)-2-hydroxyoctanoic acid	Sigma	H7396
3,7-Dimethyl-6-octenoic acid [(S)-(-)-citronellic acid]	Sigma	364428
3,7-Dimethyl-6-octenoic acid [(R)-(-)-citronellic acid]	Sigma	340499
3,7-dimethyl-2,6-octadienoic acid (geranic acid)	Sigma	W412101
8-nonenoic acid	Sigma	715433
4-methylnonanoic acid	Sigma	W357405
9-decenoic acid	Sigma	W366005
10-Undecenoic acid	Sigma	124672
cis-9-Octadecenoic acid (oleic acid)	Sigma	O1008
sodium 8-(2-hydroxybenzamido)octanoate [Salcaprozate sodium (SNAC)]	MEDCHEMEXPRESS	Cat. No.: HY-114299
sodium octanoate	Sigma	C5038
sodium decanoate	Sigma	C4151
sodium dodecanoate	Sigma	L9755
(-)-menthol	Sigma	63660
(R)-(+)-limonene	Sigma	183164
glycol chitosan	SantaCruz Biotechnology Inc.	sc-215100A
Pluronic P123	Sigma	435465
Pluronic F68 MW 8400	Sigma	412325
Pluronic F127	Sigma	P2443

Pluronic F108	Sigma	542342
Labrasol	Gattefosse	
Tween 20	Sigma	P9416
Tween 40	Sigma	P1504
Tween 80	Sigma	P1754
Triton X-100	Sigma	T9284
β-cyclodextrin	Sigma	C4767
α-cyclodextrin	Sigma	C4642
γ-cyclodextrin	Sigma	C4892
(2-Hydroxypropyl)-β-cyclodextrin	Sigma	332607
methyl-beta-cyclodextrin	Sigma	C4555
sorbitol	Sigma	240850
D-(+)-trehalose dihydrate	Sigma	T0167
sucralose	Sigma	69293
maltodextrin	Sigma	419672
D-(+)-Maltose monohydrate	Sigma	M5885
D-(+)-Glucose monohydrate	Sigma	49159
PEG 4600	Sigma	373001
PEG 1500	Sigma	86101
PEG 3350	Sigma	88276
PEG 1000	Sigma	202428
PEG 2000 methyl ether	Sigma	202509
PEG 5000 methyl ether	Sigma	81323
PEG 2000 dimethyl ether	Sigma	445908
Kollisonv PEG1450	BASF	50424112
glycine	Sigma	G7126
L-threonine	Sigma	T8625
taurine	Sigma	T0625
L-Cysteine hydrochloride	Sigma	C1276
β-Alanine	Sigma	146064
L-glutamic acid monosodium salt monohydrate	Sigma	49621
L-lysine	Sigma	L5501
serine	Sigma	S4500
thymidine	Sigma	T9250
L-methionine	MP BIOMEDICAL	4060-712
L-proline	Sigma	P0380
cytidine	Sigma	C122106
trans-4-hydroxy-l-proline	TCI	H0296
N-acetyl-L-cysteine	Sigma	A7250
L-Histidine HCl monohydrate	MP	4060-312
citric acid	Sigma	251275
glycolic acid	Sigma	124737
maleic acid	Sigma	M0375
L-(+)- tartaric acid	Sigma	T1807
5-sulfoisopthalic acid sodium salt	Sigma	142794
lignosulfonic acid, acetate sodium salt	Sigma	370983
D-gluconic acid-lactone	Sigma	G4750
Hyaluronic acid sodium salt from Streptococcus equi	Sigma	53747
inulin from chicory	Sigma	I2255
NaCl	Sigma	S9888
guar gum	Sigma	G4129
acacia	Spectrum	GU117

sodium carboxymethylcellulose MW 250000 degree of substitution 0.9	Sigma	419303
sodium carboxymethylcellulose MW 250000 degree of substitution 1.2	Sigma	419281
hydroxypropyl cellulose MW 100,000	Alfa Aesar	43400
carboxymethyl cellulose sodium salt MW 700,000	Sigma	419338
sodium carboxymethyl cellulose MW 90,000	Sigma	419273
hydroxyethyl cellulose (800-1500 mPas- 2% in water)	TCI	H0418

Table S2. List of molecular features with the largest SHAP values and their description. Unless otherwise stated, all are extracted from RDKit.

Feature Name	Description
MinAbsPartialCharge	Refers to the minimum absolute partial charge calculated for a molecule. The descriptor quantifies the smallest absolute value of the partial charges assigned to the atoms within a molecule. These partial charges typically arise from the asymmetric distribution of electrons across the chemical bonds within the molecule
Water solubility	Water solubility value, extracted from pkCSM
fr_C_O_noCOO	Number of carbonyl O, including COO-, excluding COOH
NumRotatableBonds	Number of rotatable bonds for a molecule. A large value can be associated with higher three-dimensionality.
surface area	Total Surface Area of the compound, extracted from pkCSM
MinPartialCharge	Minimum partial charge, similar to 'MinAbsPartialCharge' but captures the minimum instead of the minimum absolute partial charge
NumAliphaticRings	Number of aliphatic (containing at least one non-aromatic bond) rings for a molecule
FractionCSP3	proportion of carbon atoms in a molecule that are *sp*3 hybridized, which implies that these carbons are bonded to four other atoms, typically with single bonds, as opposed to *sp*2 or *sp* hybridized carbons, which are involved in double or triple bonds, respectively. A higher FractionCSP3 value can be associated with a greater degree of three-dimensionality and complexity in molecular structures, which is a desirable feature in drug candidates for certain drug discovery efforts
MinAbsEStateIndex	Refers to the minimum absolute value of the electrotopological state indices for the atoms in a molecule. These indices take into account the electronic environment and the topological state of atoms in a molecule, which include both the intrinsic state of an atom and perturbations due to its interactions with neighboring atoms.

Table S3. List of the top 40 most highly correlated physiochemical features along with their raw correlation values.

Feature	Pearson Correlation
surface area	0.026200508
LabuteASA	0.02620063
NumAliphaticRings	0.026505657
NumSaturatedRings	0.026765525
TPSA	-0.027474238
NumRotatableBonds	-0.02760814
NumHeteroatoms	-0.028151638
NumHAcceptors	-0.029846038
fr_NH0	-0.029908986
fr_aldehyde	-0.030102503
fr_halogen	-0.030563398
fr_C_O	0.031854518
fr_benzene	-0.032619443
fr_Ar_N	-0.033094066
NHOHCount	-0.033154599
NOCount	-0.033471947
NumHDonors	-0.033631965
fr_ester	-0.034988469
charge	0.036554462
bond_stereo_count	-0.042719945
fr_ether	-0.046905181
NumAromaticRings	-0.04743422
fr_COO2	0.054306809
fr_COO	0.054306809
fr_Al_COO	0.060937041
fr_NH2	-0.062574962
fr_allylic_oxid	-0.088508931
MinPartialCharge	-0.08923324
MaxAbsEStateIndex	0.092823689
MaxEStateIndex	0.092823689
Water solubility	-0.131414872
FractionCSP3	0.140373557
MinAbsEStateIndex	-0.142483429
covalent_unit_count	0.156234596
MinAbsPartialCharge	0.192008349
AvgIpc	0.203547637
fr_C_O_noCOO	0.21260545
BalabanJ	-0.246823541
MaxAbsPartialCharge	0.326374735
MaxPartialCharge	0.327966602

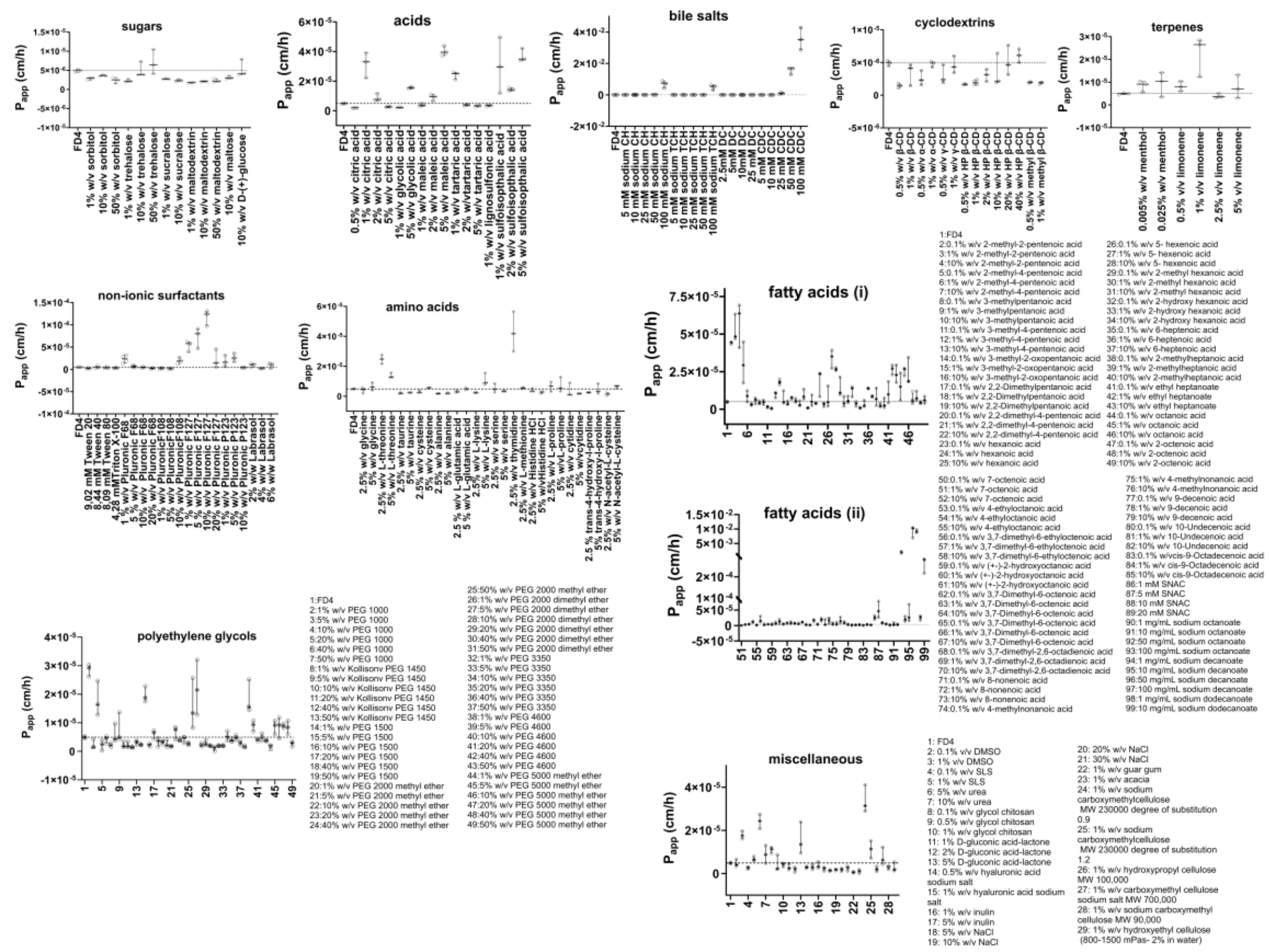

Figure S1. ***Ex vivo*** **permeability excipient screening study across porcine esophageal mucosa.** Different classes of excipients (sugars, amino acids, terpenes, bile salts, non ionic surfactants, acids, cyclodextrins, fatty acids, polyethylene glycols, miscellaneous) were evaluated at increasing concentrations for their effect on the permeability of fluorescein-isothiacyanate dextran 4 kDa (FD4) across porcine esophagus. The dashed line denotes the permeability baseline based on the apparent permeability value (P_{app}) calculated for FD4. Data are shown as individual data points of n=3 independent biological replicates, unless otherwise noted (10mM deoxycholic acid sodium salt: n=5, 1 % w/v Pluronic F68: n=4, 5 % w/v Pluronic F68: n=5, 1% w/v hydroxypropyl β-cyclodextrin: n=4, 0.1% w/w 3-methyl-2-oxopentanoic acid: n=4, 0.1% w/w 2-octenoic acid: n=4, 0.1% w/w 4-methylnonanoic acid: n=4). The central horizontal line indicates the median, and the vertical bars represent the interquartile range (25th–75th percentiles, IQR).

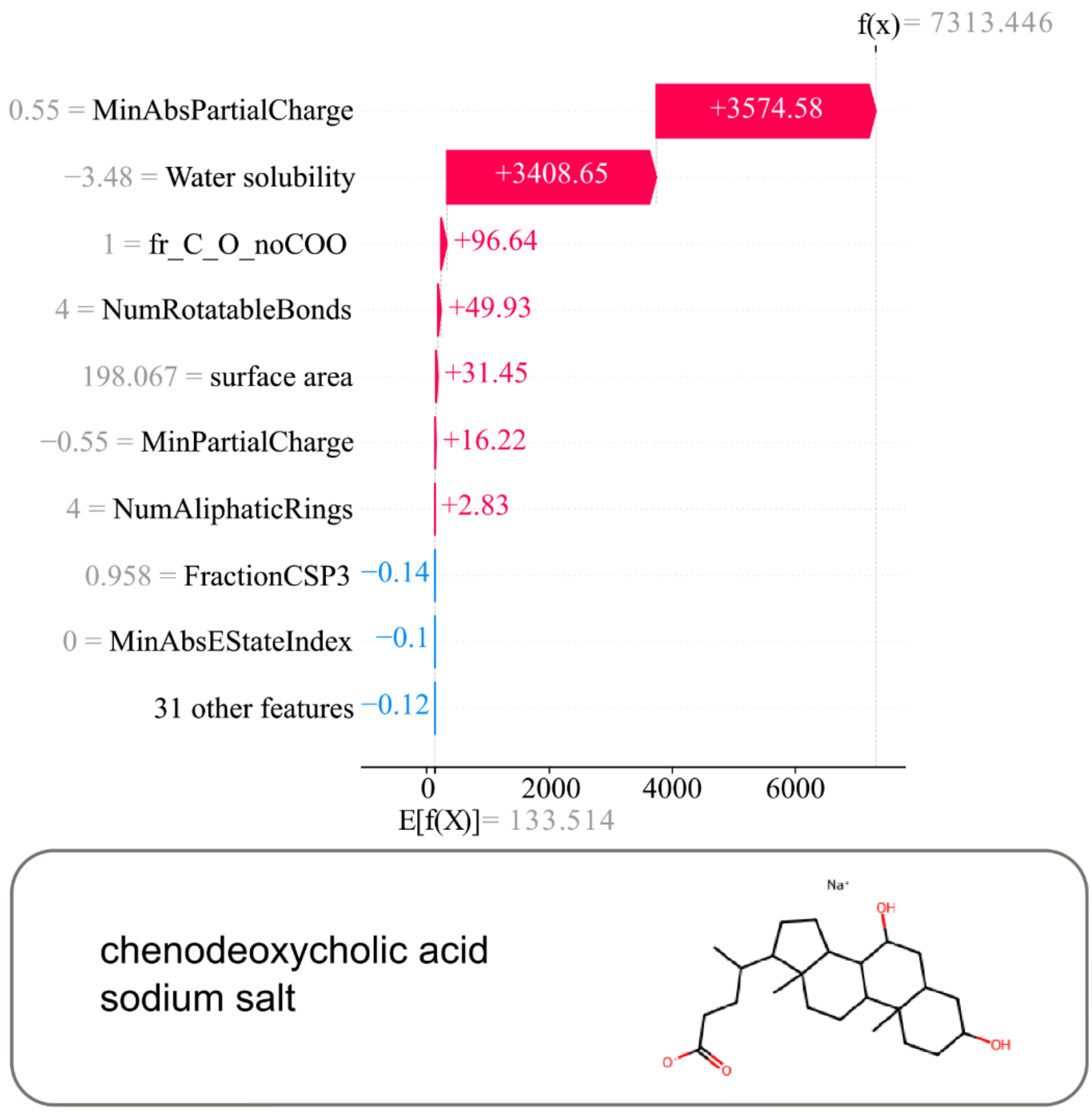

Figure S2. SHAP explanations for chenodeoxycholic acid sodium salt.

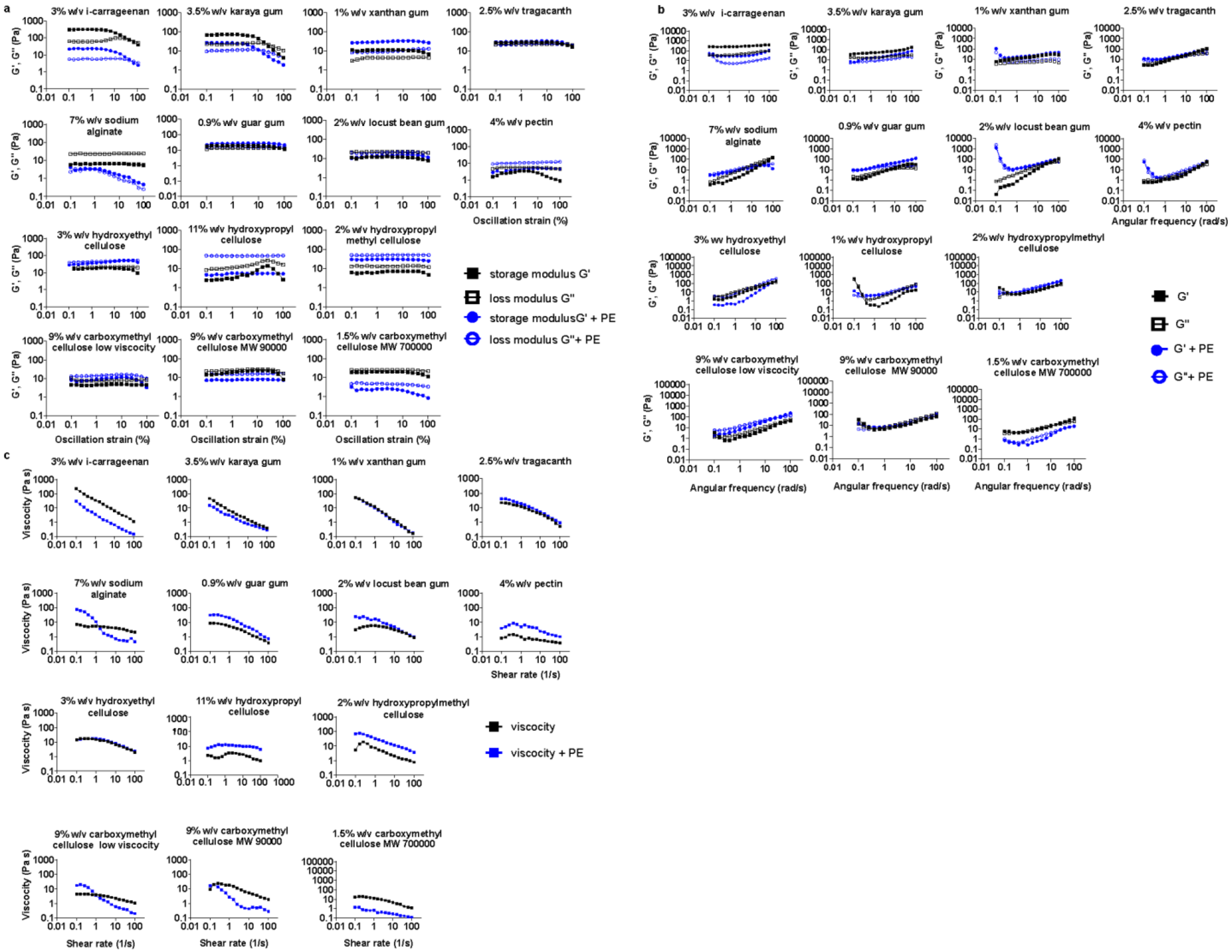

a
3% w/v i-carrageenan
3.5% w/v karaya gum
1% w/v xanthan gum
2.5% w/v tragacanth
7% w/v sodium alginate
0.9% w/v guar gum
2% w/v locust bean gum
4% w/v pectin
3% w/v hydroxyethyl cellulose
11% w/v hydroxypropyl cellulose
2% w/v hydroxypropyl methyl cellulose
9% w/v carboxymethyl cellulose low viscocity
9% w/v carboxymethyl cellulose MW 90000
1.5% w/v carboxymethyl cellulose MW 700000
G', G'' (Pa)
Oscillation strain (%)
storage modulus G'
loss modulus G''
storage modulusG' + PE
loss modulus G''+ PE
b
3% w/v i-carrageenan
3.5% w/v karaya gum
1% w/v xanthan gum
2.5% w/v tragacanth
7% w/v sodium alginate
0.9% w/v guar gum
2% w/v locust bean gum
4% w/v pectin
3% w/v hydroxyethyl cellulose
1% w/v hydroxypropyl cellulose
2% w/v hydroxypropylmethyl cellulose
9% w/v carboxymethyl cellulose low viscocity
9% w/v carboxymethyl cellulose MW 90000
1.5% w/v carboxymethyl cellulose MW 700000
G', G'' (Pa)
Angular frequency (rad/s)
G'
G''
G' + PE
G''+ PE
c
3% w/v i-carrageenan
3.5% w/v karaya gum
1% w/v xanthan gum
2.5% w/v tragacanth
7% w/v sodium alginate
0.9% w/v guar gum
2% w/v locust bean gum
4% w/v pectin
3% w/v hydroxyethyl cellulose
11% w/v hydroxypropyl cellulose
2% w/v hydroxypropylmethyl cellulose
9% w/v carboxymethyl cellulose low viscocity
9% w/v carboxymethyl cellulose MW 90000
1.5% w/v carboxymethyl cellulose MW 700000
Viscocity (Pa s)
Shear rate (1/s)
viscocity
viscocity + PE

Figure S3. Rheological properties of the hydrogels with and without the presence of the PEs (25 mM chenodeoxycholic acid sodium salt and 100 mM sodium cholate hydrate). a. The linear viscoelastic region (LVR) of the hydrogels was determined by strain sweeps at 10 rad/s from 0.1-100%. Data are presented as mean of n=3 independent biological replicates. **b.** The dynamic rheological behavior was monitored by oscillatory angular frequency sweeps in the range of 0.1–100 rad/s within the LVR (1% strain). Data are presented as mean of n=3 independent biological replicates. **c.** The flow curves of the hydrogels were obtained by shear rate sweeps between 0.1–100 1/s. Each line represents the arithmetic mean of two independent rheometer runs. Data are presented as mean of n=3 independent biological replicates.

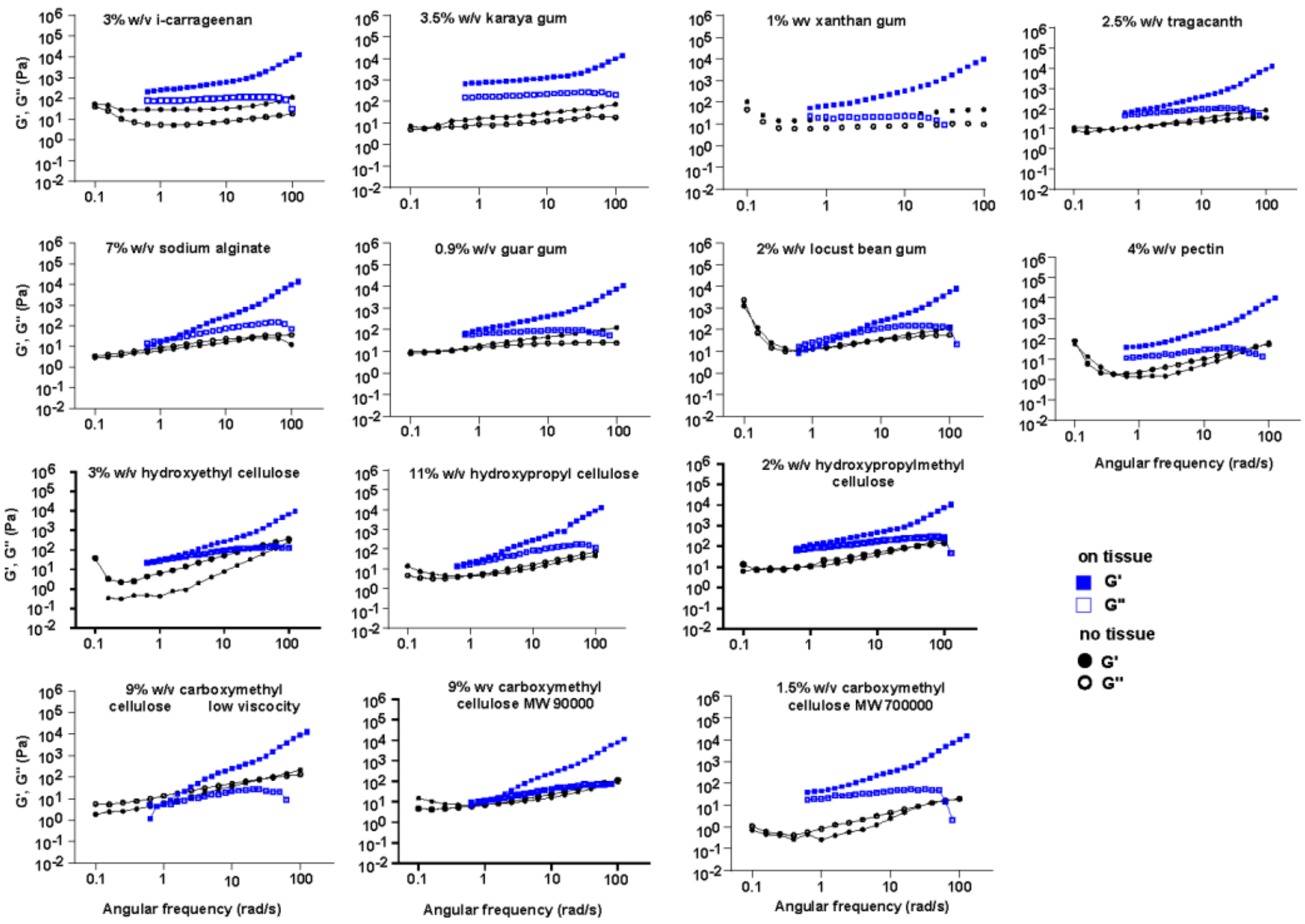

Figure S4. ***Ex vivo*** **assessment of the bioadhesive properties of the hydrogels.** Adhesion properties were tested using rheology. The hydrogels were mounted between porcine esophageal mucosae attached on the bottom and upper plates of the rheometer. Frequency sweeps were performed over the frequency range of 0.1 Hz to 100 Hz at a constant 1% strain with and without the presence of the esophageal mucosa. Data are presented as mean of n=3 independent biological replicates.

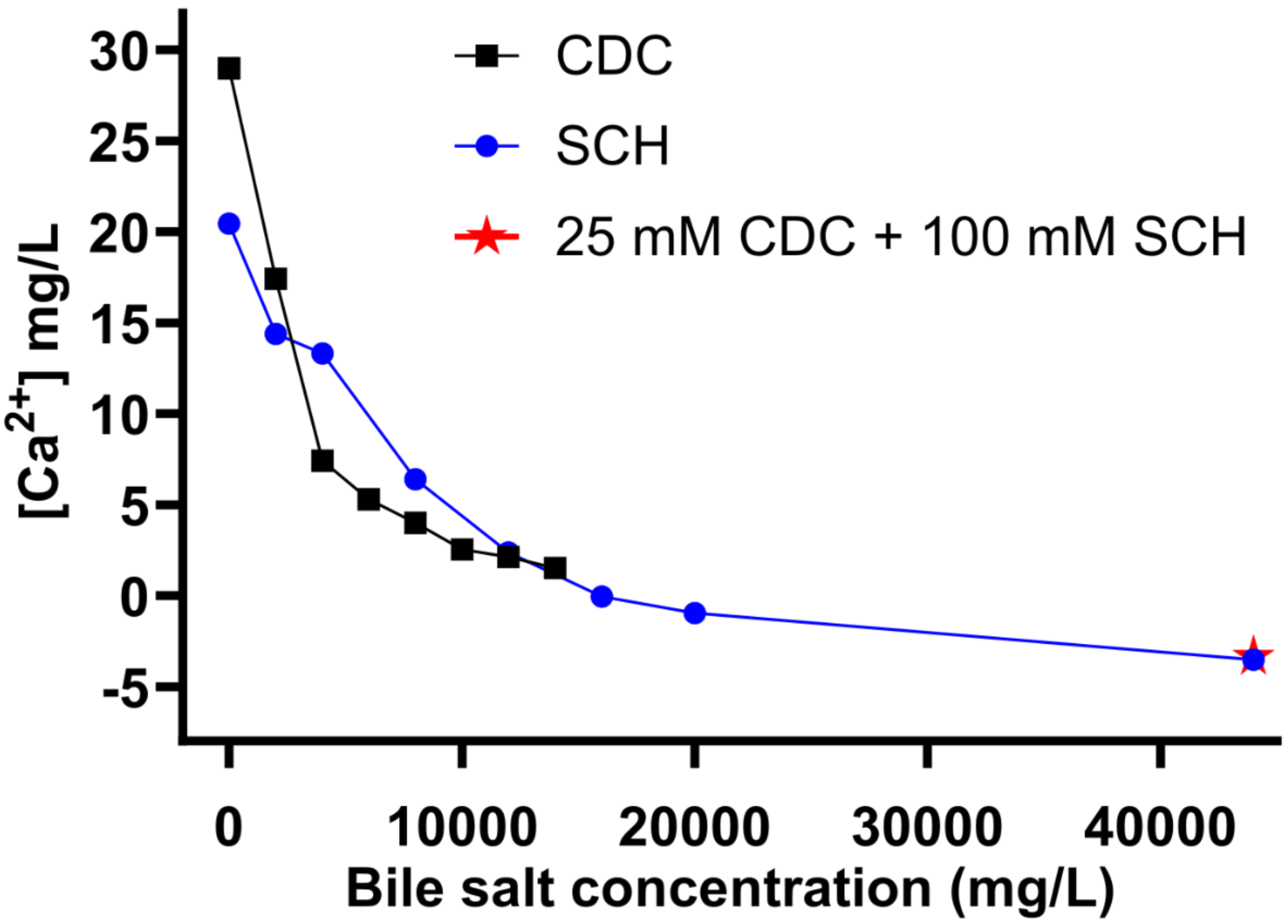

Figure S5. Bile salt interraction with calcium ions. Calcium levels were measured spectrophotometrically using the Ca^{2+}-sensitive dye Pontachrome Violet SW at increasing bile salt concentrations and in the presence of the binary mixture chenodeoxycholic acid sodium salt (CDC) at 25 mM and sodium cholate hydrate (SCH) at 100 mM. Data are presented as mean of n=3 independent biological replicates.

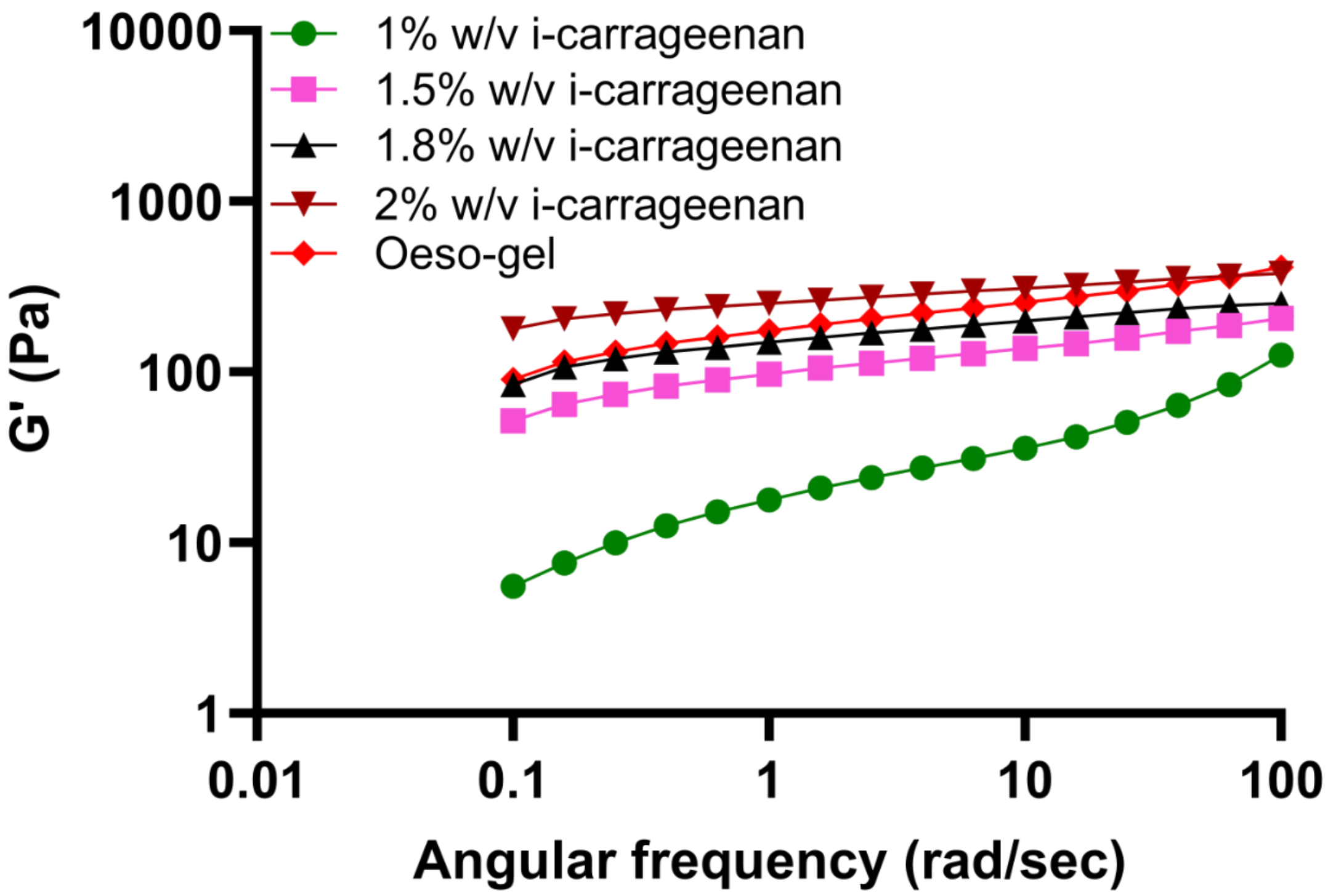

Figure S6. Rheological properties of Oeso-gel and i-carrageenan hydrogels. Frequency sweep measurements of increasing concentrations of i-carrageenan hydrogels (without PEs) and Oeso-gel were performed over the frequency range of 0.1 Hz to 100 Hz at a constant 1% strain. Each line represents the arithmetic mean of n=3 independent biological replicates.

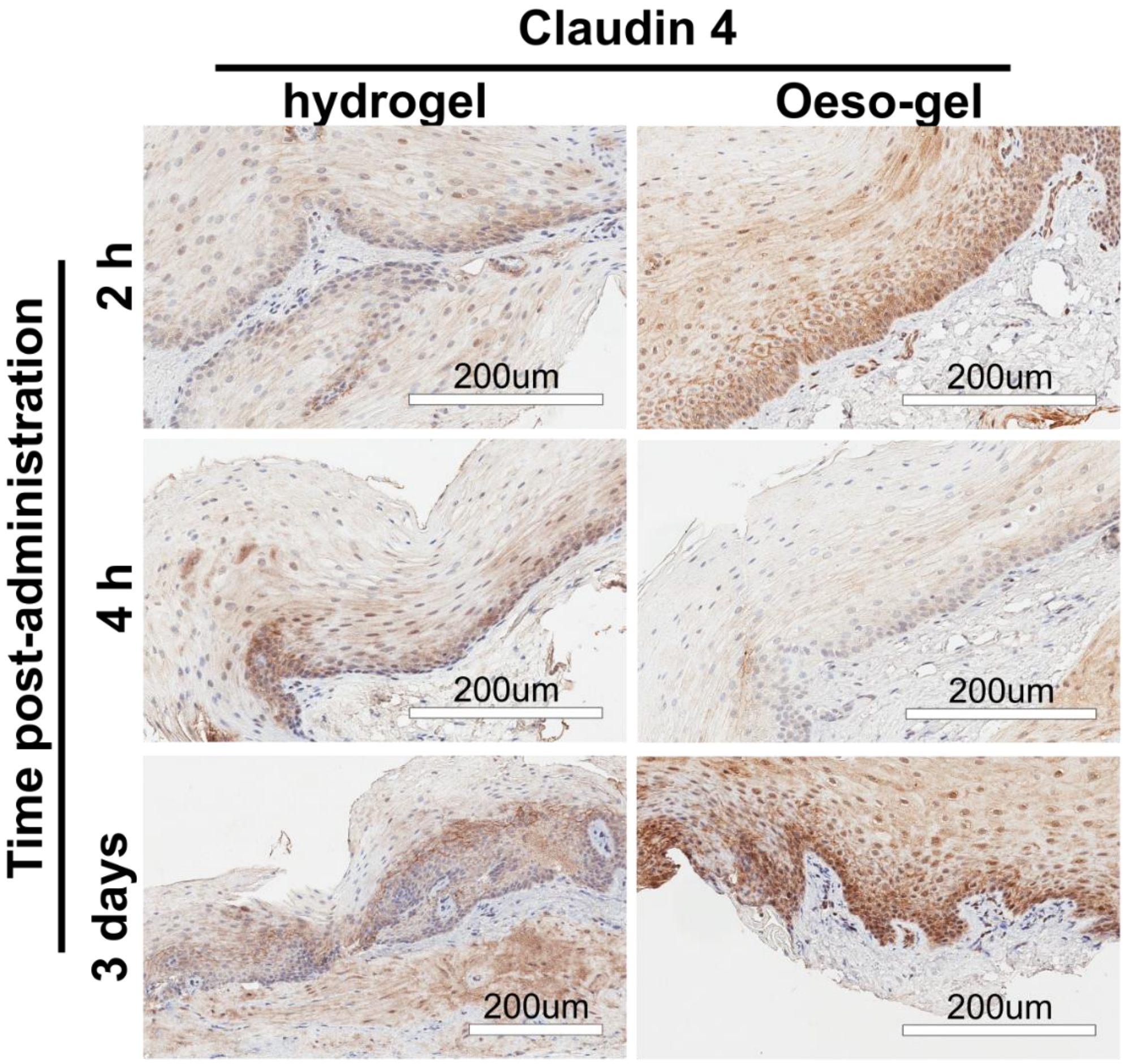

Figure S7. Representative immunohistochemical staining of Claudin 4 after the *in vivo* administration of the infliximab-loaded hydrogel (no PEs) and Oeso-gel to the esophagus of swine. Similar results were obtained from n = 2 independent biological replicates.

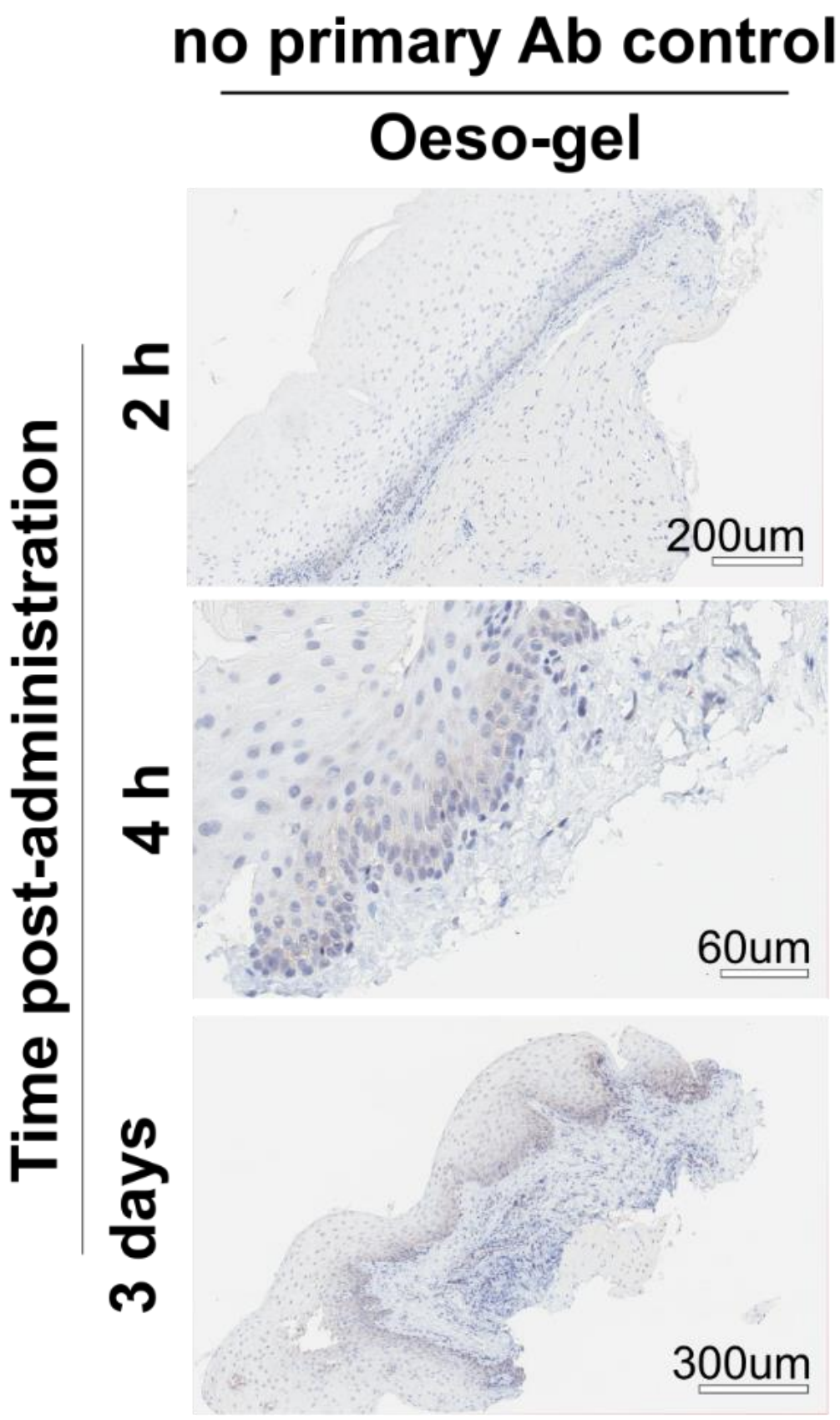

Figure S8. Representative immunohistochemistry control (no primary antibody-infliximab-control) on the esophageal biospies collected from the animals to which Oeso-gel was administered. Similar results were obtained from n = 2 independent biological replicates.

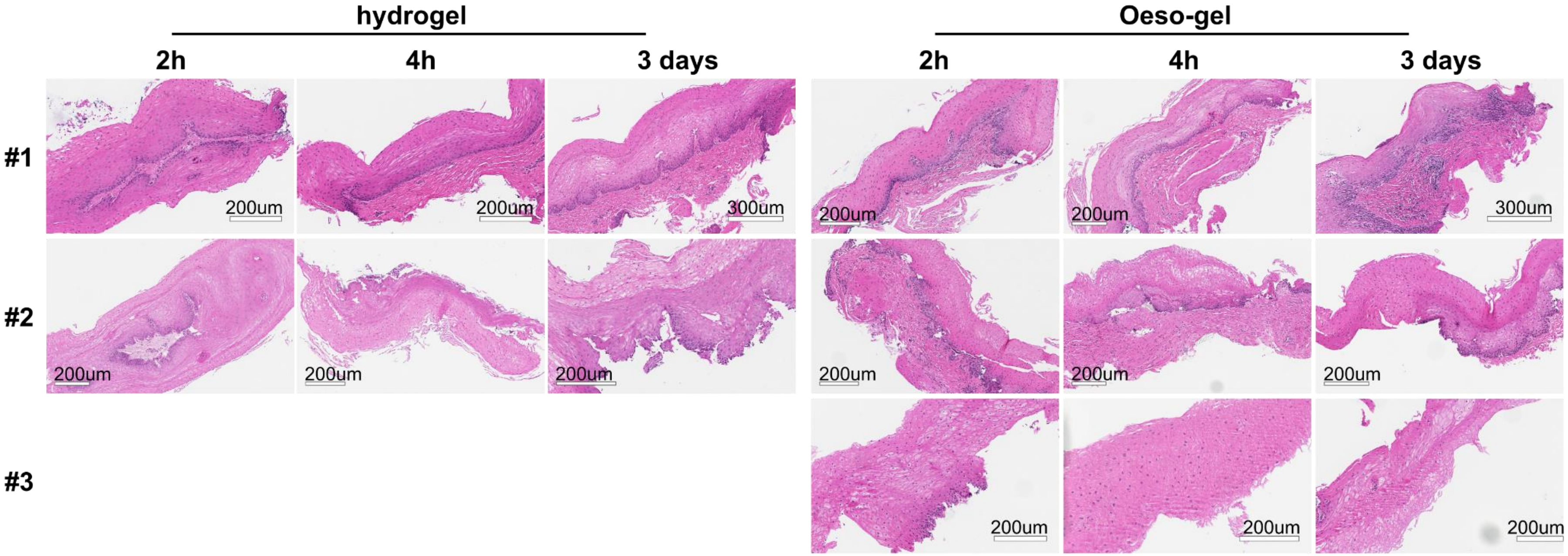

Figure S9. Hematoxylin and eosin (H&E) staining of all oesophageal biopsies collected after treatment with the infliximab-loaded hydrogel (no PEs, n=2 independent biological replicates) and Oeso-gel (n=3 independent biological replicates).

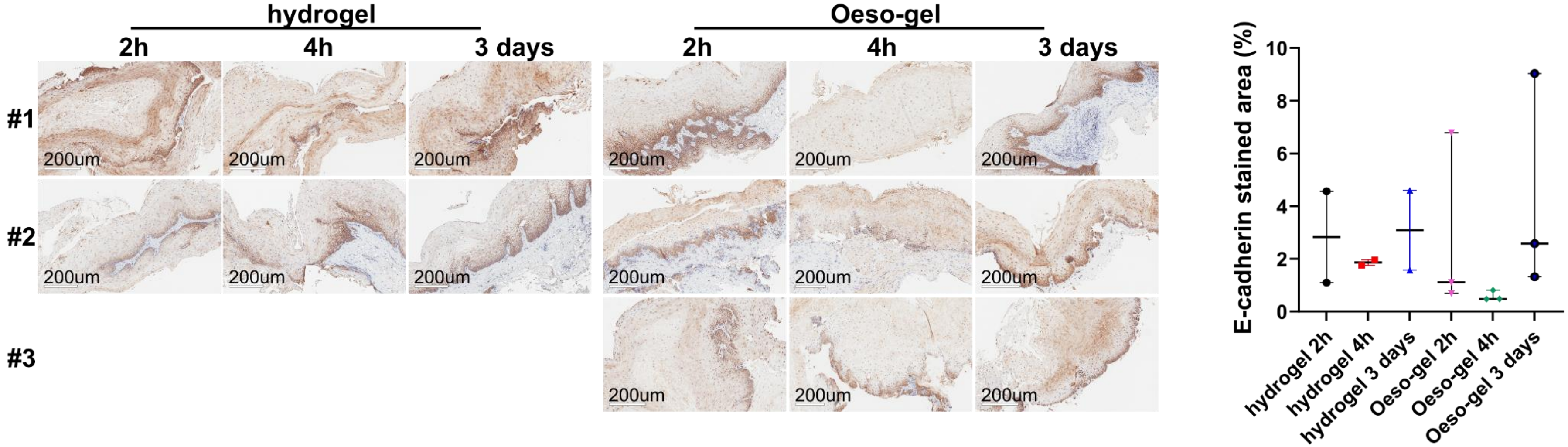

Figure S10. Immunohistochemical staining of E-cadherin in all oesophageal biopsies collected after treatment with the infliximab-loaded hydrogel (no PEs, n=2 independent biological replicates) and Oeso-gel (n=3 independent biological replicates). Data are shown as individual data points. The central horizontal line indicates the median, and the vertical bars represent the interquartile range (25th–75th percentiles, IQR). Each data point represents one independent biological replicate (hydrogel: n=2, Oeso-gel: n=3).

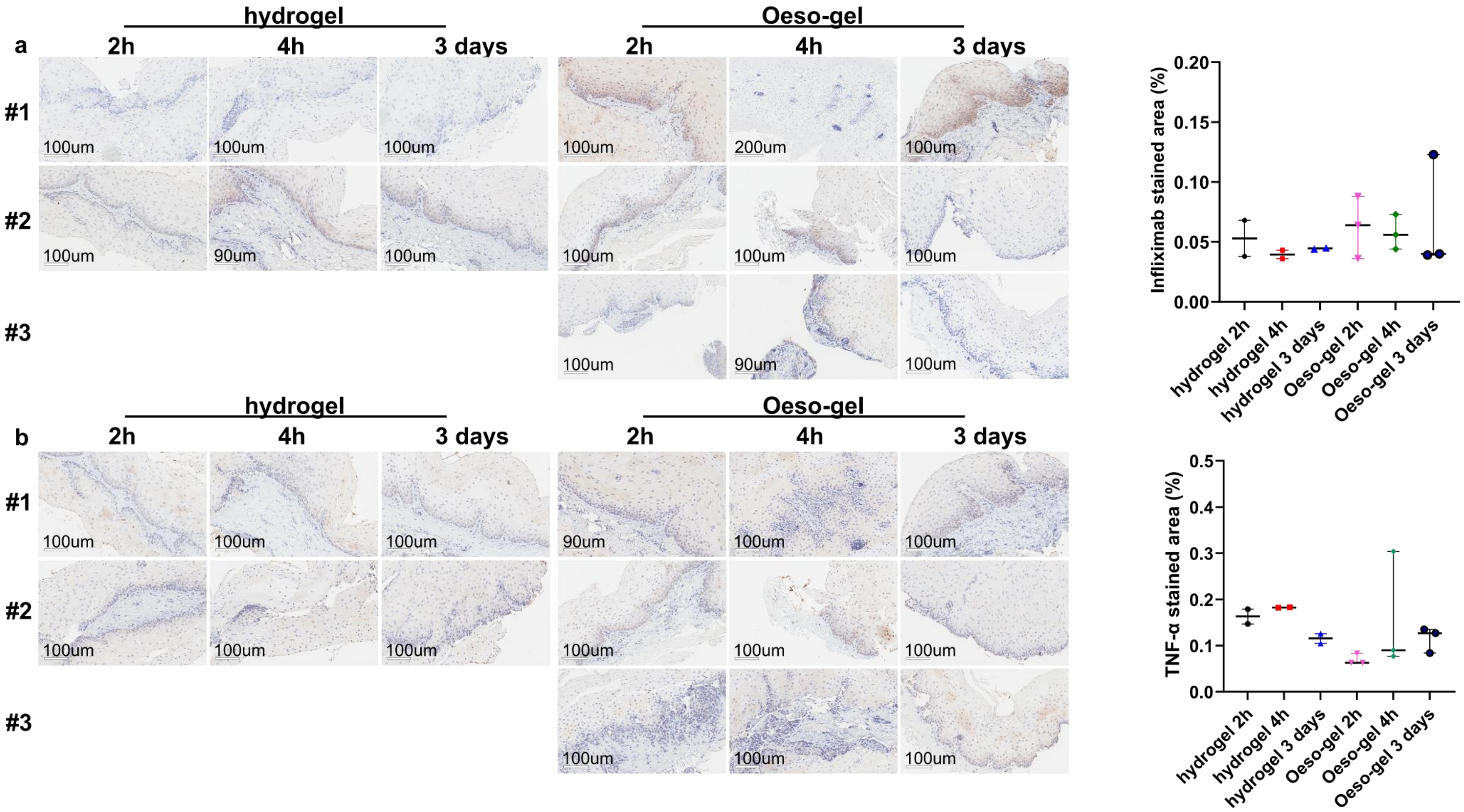
a
b
hydrogel
Oeso-gel
2h
4h
3 days
#1
#2
#3
100um
200um
90um
Infliximab stained area (%)
0.20
0.15
0.10
0.05
0.00
TNF-α stained area (%)
0.5
0.4
0.3
0.2
0.1
0.0
hydrogel 2h
hydrogel 4h
hydrogel 3 days
Oeso-gel 2h
Oeso-gel 4h
Oeso-gel 3 days

Figure S11. Immunohistochemical staining of **(a)** infliximab and **(b)** TNF-α in all oesophageal biopsies collected after treatment with the infliximab-loaded hydrogel (no PEs, n=2 independent biological replicates) and Oeso-gel (n=3 independent biological replicates). The central horizontal line indicates the median, and the vertical bars represent the interquartile range (25th–75th percentiles, IQR). Each data point represents one independent biological replicate (hydrogel: n=2, Oeso-gel: n=3).

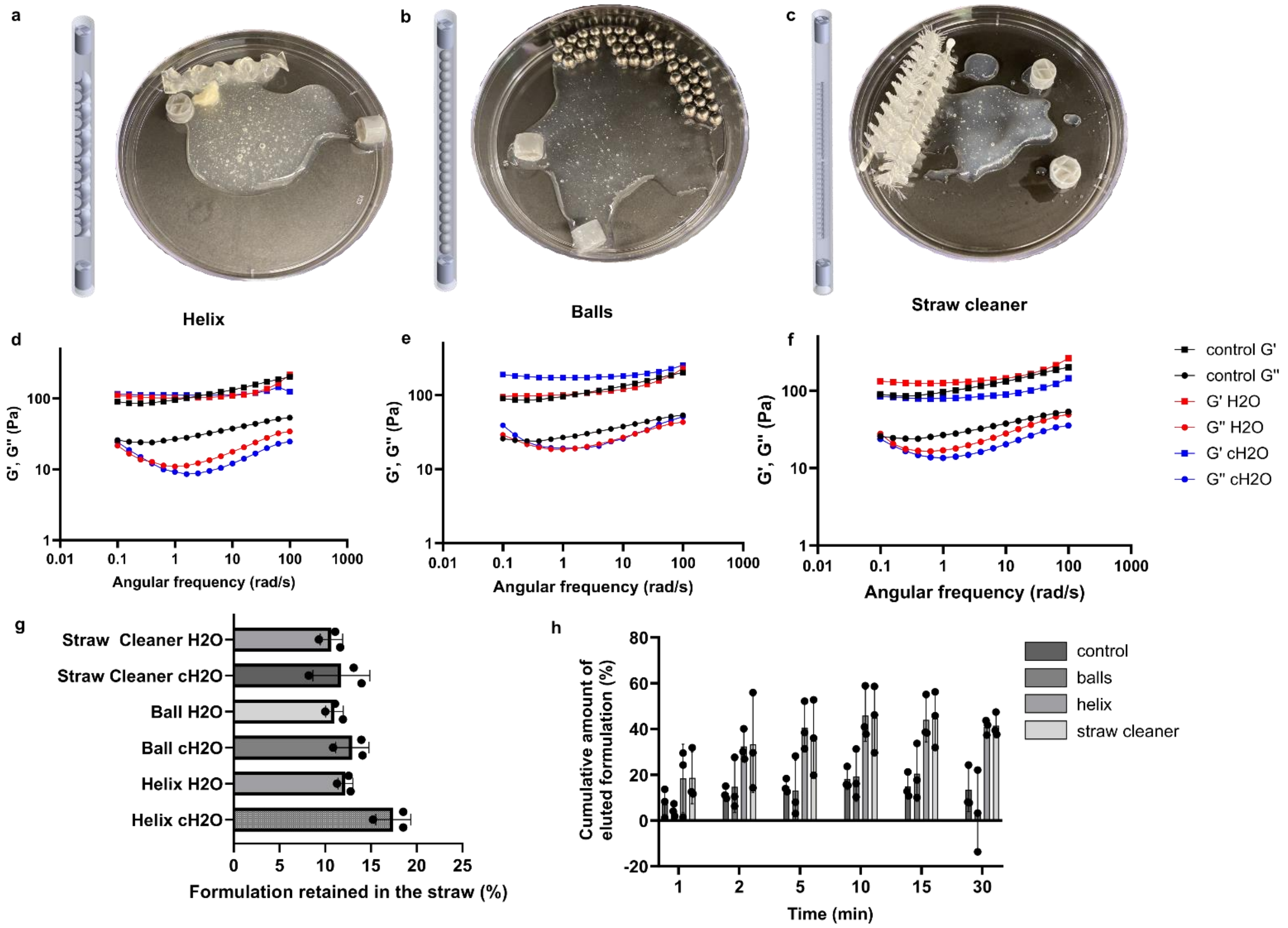
a
b
c
Helix
Balls
Straw cleaner
d
e
f
G', G'' (Pa)
Angular frequency (rad/s)
control G'
control G''
G' H2O
G'' H2O
G' cH2O
G'' cH2O
g
Straw Cleaner H2O
Straw Cleaner cH2O
Ball H2O
Ball cH2O
Helix H2O
Helix cH2O
Formulation retained in the straw (%)
h
Cumulative amount of eluted formulation (%)
Time (min)
control
balls
helix
straw cleaner

Figure S12. Development of a straw device for long-term storage and administration of Oeso-gel. Different static mixer designs were tested for their efficacy to reconstitute the freeze-dried formulation into a hydrogel with similar properties to the freshly prepared formulations. **(a)** The helix mixer resulted in inhomogeneous reconstitution and the formation of non-hydrated agglomerates. **(b)** The ball mixers resulted in homogenous and well mixed hydrogels, similar to the **(c)** straw cleaner-type mixer. Frequency sweeps were performed to compare the rheological properties of the reconstituted (with either plain or carbonated water) Oeso-gel to the freshly prepared control after using the **(d)** helix type mixer, **(e)** the ball mixers and **(f)** the straw cleaner-type mixer. Each line represents the arithmetic mean of three independent biological replicates. **(g)** The amount of hydrogel retained in the straw was calculated after reconstitution with plain or carbonated water and with different types of static mixers. Data are presented as mean ± S.D. of n=3 independent biological replicates. **(h)** The retention of the reconstituted hydrogels on the esophagus was tested *ex vivo* using the vertical plate configuration and was compared to the freshly prepared control. The freeze-dried formulation reconstituted using the ball mixers showed similar mucoadhesion to the control. Data are presented as mean ± S.D. of n=3 independent biological replicates.
