# Supplementary material for: Oesophageal tissue screening system for assessing the retention and mucosal absorption of biologics
Source: Nat Biomed Eng. 2026 Jun 12;10(10):2213–29. doi: 10.1038/s41551-026-01685-9 (PMC13672101; doi:10.1038/s41551-026-01685-9)
Supplement: Supplementary file 2 [file 41551_2026_1685_MOESM2_ESM.pdf]

nature portfolio

Corresponding author(s): Giovanni Traverso

Last updated by author(s): Apr 9, 2026

Reporting Summary

Nature Portfolio wishes to improve the reproducibility of the work that we publish. This form provides structure for consistency and transparency in reporting. For further information on Nature Portfolio policies, see our Editorial Policies and the Editorial Policy Checklist.

Statistics

For all statistical analyses, confirm that the following items are present in the figure legend, table legend, main text, or Methods section.

n/a	Confirmed	
☐	☒	The exact sample size (*n*) for each experimental group/condition, given as a discrete number and unit of measurement
☐	☒	A statement on whether measurements were taken from distinct samples or whether the same sample was measured repeatedly
☐	☒	The statistical test(s) used AND whether they are one- or two-sided<br>*Only common tests should be described solely by name; describe more complex techniques in the Methods section.*
☒	☐	A description of all covariates tested
☒	☐	A description of any assumptions or corrections, such as tests of normality and adjustment for multiple comparisons
☐	☒	A full description of the statistical parameters including central tendency (e.g. means) or other basic estimates (e.g. regression coefficient) AND variation (e.g. standard deviation) or associated estimates of uncertainty (e.g. confidence intervals)
☐	☒	For null hypothesis testing, the test statistic (e.g. *F*, *t*, *r*) with confidence intervals, effect sizes, degrees of freedom and *P* value noted<br>*Give P values as exact values whenever suitable.*
☒	☐	For Bayesian analysis, information on the choice of priors and Markov chain Monte Carlo settings
☒	☐	For hierarchical and complex designs, identification of the appropriate level for tests and full reporting of outcomes
☐	☒	Estimates of effect sizes (e.g. Cohen's *d*, Pearson's *r*), indicating how they were calculated

Our web collection on statistics for biologists contains articles on many of the points above.

Software and code

Policy information about availability of computer code

Data collection	Molecular features for molecules are extract with rdkit 2022.09.1 (https://github.com/rdkit/rdkit).
Data analysis	The machine learning model was developed with xgboost 1.5.0 (https://github.com/dmlc/xgboost/).<br>Explanation visualizations were generated with shap 0.42.1 (https://github.com/shap/shap).<br>Data analysis was performed using GraphPad Prism 10 (GraphPad Software, San Diego, CA, USA).<br>CAD design was performed using SolidWorks 2022 SP0 (Dassault Systèmes SolidWorks Corp.).<br>Sllicing was performed using PreForm software version 3.28.0 (Formlabs).

For manuscripts utilizing custom algorithms or software that are central to the research but not yet described in published literature, software must be made available to editors and reviewers. We strongly encourage code deposition in a community repository (e.g. GitHub). See the Nature Portfolio guidelines for submitting code & software for further information.

Data

Policy information about availability of data

All manuscripts must include a data availability statement. This statement should provide the following information, where applicable:

- Accession codes, unique identifiers, or web links for publicly available datasets
- A description of any restrictions on data availability
- For clinical datasets or third party data, please ensure that the statement adheres to our policy

All the data supporting the results in this study are available within the paper and its Supplementary Information. Source data for main figures are provided within the paper.

Research involving human participants, their data, or biological material

Policy information about studies with human participants or human data. See also policy information about sex, gender (identity/presentation), and sexual orientation and race, ethnicity and racism.

Reporting on sex and gender	The study used de-identified ex vivo human esophageal tissues obtained from deceased donors through the National Disease Research Interchange (NDRI). No living human participants were involved.Human esophageal tissues were obtained from six deceased donors: three male and three female.
Reporting on race, ethnicity, or other socially relevant groupings	Five donors were reported as Caucasian and one donor as Black, according to NDRI documentation. This information was provided by the tissue bank and was not used for sample selection, grouping, or analysis.
Population characteristics	Donor ages ranged from 28 to 62 years. All donors had varying medical histories unrelated to sample selection criteria. Tissues were provided fully de-identified by NDRI.
Recruitment	No recruitment was performed. NDRI procured tissues from next-of-kin–authorized deceased donors and supplied fully de-identified specimens.
Ethics oversight	All tissues were obtained through the National Disease Research Interchange (NDRI), an NIH-funded human tissue procurement organization. NDRI confirmed next-of-kin authorization for research use for each donor ("Research Authorization Obtained: Yes" in all donor files). All biospecimens were fully de-identified before receipt. Research involving de-identified tissues obtained from deceased donors does not constitute human subjects research and is exempt from IRB oversight.

Note that full information on the approval of the study protocol must also be provided in the manuscript.

Field-specific reporting

Please select the one below that is the best fit for your research. If you are not sure, read the appropriate sections before making your selection.

☒ Life sciences ☐ Behavioural & social sciences ☐ Ecological, evolutionary & environmental sciences

For a reference copy of the document with all sections, see nature.com/documents/nr-reporting-summary-flat.pdf

Life sciences study design

All studies must disclose on these points even when the disclosure is negative.

Sample size	All in vitro experiments were performed with three independent replicates (n = 3), unless otherwise stated. Ex vivo experiments were performed using tissues from a minimum of three independent biological samples. In some ex vivo experiments, additional replicates (n > 3) were conducted to compensate for occasional technical failures inherent to tissue-based permeability assays (e.g. loss of tissue integrity, incomplete sealing, or procedural artifacts). These failed runs were identified based on predefined technical criteria and were not included in the analysis. In vivo studies: In our study, we aimed to investigate the effects of the treatment in a large animal model, specifically swine, positioned in a semi-orthostatic position to closely mimic the human esophagus orientation. Due to the inherent challenges associated with working with large animals, including logistical constraints and the need for precise positioning during the experiments, for the treated group, we utilized three animals (n=3), while the control group consisted of two animals (n=2). This sample size was chosen based on practical considerations and the complexity of conducting experiments in a large animal model. The semi-orthostatic positioning and monitoring of swine during experiments pose substantial logistical challenges, which further constrained the number of animals that could be ethically and practically included.
Data exclusions	Technical failures identified a priori (e.g. loss of tissue integrity or assay malfunction) were not considered valid experimental replicates and were therefore not included in the analysis. No valid biological data were excluded.
Replication	All in vitro experiments were performed with three independent replicates (n = 3). Ex vivo experiments (including permeability, retention and mucoadhesion studies) were conducted using tissues from three independent biological samples. Due to the complexity of ex vivo tissue-based assays, occasional technical failures (e.g., loss of tissue integrity, or procedural artifacts) occurred and were predefined as invalid prior to data analysis. Additional replicates were therefore performed as needed to obtain the target number of valid biological replicates, resulting

in n > 3 for selected conditions. No valid experimental data were excluded from the analyses. The ex vivo experiment using porcine oesophageal tissue and X-ray imaging to visualize formulation retention over time was performed once (Figure 5c). This experiment was qualitative and illustrative in nature and was not used for statistical analysis. In vivo experiments were performed in a swine model with two animals in the control group (n = 2) and three animals in the treatment group (n = 3). All replication attempts were successful. Endoscopic visualization of formulation retention in the porcine oesophagus was performed in a single animal to qualitatively assess formulation positioning and retention in vivo (Figure 7a). Repeated endoscopic insertion was not feasible, as the endoscope mechanically disrupted and partially removed the formulation, thereby altering the experimental system. Accordingly, this experiment was designed as an illustrative, qualitative assessment and was not intended for quantitative analysis or statistical comparison. Histological and immunohistochemical stainings (H&E, immunohistochemistry and immunofluorescence) were performed on two independent biological samples for ex vivo experiments (n = 2) using porcine tissue and on tissues from three treated animals (n = 3) and two control animals (n = 2) for in vivo studies. For human oesophageal histology, biological replication was limited by tissue availability; therefore, control and hydrogel conditions were analysed in one independent biological sample (IHC and H&E), while two independent biological samples were available for the Oeso-gel condition.

Randomization

Randomization was not applicable to in vitro and ex vivo experiments, as samples were processed according to predefined experimental conditions and outcomes were not influenced by group allocation. in vivo studies: Animal allocation in the different experimental groups was random.

Blinding

For in vitro and ex vivo experiments, blinding was not relevant, as measurements were quantitative and instrument-based and samples were processed according to predefined experimental conditions rather than subjective outcome assessment. in vivo studies: The complexity of handling large animal models, particularly in a semi-orthostatic position, necessitated close monitoring and intervention, making blinding impractical.

Reporting for specific materials, systems and methods

We require information from authors about some types of materials, experimental systems and methods used in many studies. Here, indicate whether each material, system or method listed is relevant to your study. If you are not sure if a list item applies to your research, read the appropriate section before selecting a response.

Materials & experimental systems

n/a	Involved in the study
☐	☒ Antibodies
☒	☐ Eukaryotic cell lines
☒	☐ Palaeontology and archaeology
☐	☒ Animals and other organisms
☒	☐ Clinical data
☒	☐ Dual use research of concern
☒	☐ Plants

Methods

n/a	Involved in the study
☒	☐ ChIP-seq
☒	☐ Flow cytometry
☒	☐ MRI-based neuroimaging

Antibodies

Antibodies used

Primary Antibodies
1. Anti-Infliximab (HCA212). Supplier: Bio-Rad. Catalog number: HCA212Clone: AbD17837. Host: Human recombinant monoclonal. Antigen: Recombinant infliximab idiotype. Dilution used: 10 µg/mL. Lot number: 168396.
2. Anti-TNF-α (ab6671). Supplier: Abcam. Catalog number: ab6671. Clone: Polyclonal (Rb pAb)Host: Rabbit polyclonal. Antigen: Full-length recombinant human TNF (P01375)Dilution used: 1:50. Lot number: 1053654-8.
3. Claudin-4 Supplier: ThermoFisher Scientific. Catalog number: PA5-32354. Clone: Polyclonal. Host: Rabbit. Antigen: Synthetic peptide from C-terminal human Claudin-4. Dilution used: 1:10. Lot number: Not recorded.
4. E-cadherin. Supplier: ThermoFisher Scientific. Catalog number: PA5-80457. Clone: Polyclonal. Host: Rabbit. Antigen: Recombinant human E-cadherin (Met1–Ile707). Dilution used: 1:100. Lot number: UK2885776
Secondary Antibodies:
5. Anti-V5-Tag:HRP. Supplier: Bio-Rad. Catalog number: MCA1360P. Clone: SV5-Pk1. Host: Mouse monoclonal. Dilution used: 10 µg/mL. Lot number: 158243
6. Goat anti-Rabbit IgG (Alexa Fluor 488). Supplier: ThermoFisher Scientific. Catalog number: A-11008. Host: Goat polyclonal Fluorophore: Alexa Fluor 488. Concentration used: 4 µg/mL. Lot number: Not recorded

Validation

1) Human anti-Infliximab antibody (Bio-Rad HCA212, clone AbD17837): This recombinant human Fab antibody specifically recognizes the infliximab monoclonal antibody and has been characterized by the manufacturer using binding and inhibition assays. Manufacturer validation data include specificity assays demonstrating binding to infliximab and competitive or bridging ELISA formats to confirm target recognition and functional inhibition of infliximab binding. The intrinsic affinity (KD) for infliximab has been measured by real-time interaction analysis, indicating high specificity for the intended target.
2. Anti-TNF-α (ab6671): This antibody is raised against full-length recombinant human TNF-α protein and is supplied by the manufacturer with validation for immunohistochemistry based on reactivity to human TNF-α (Uniprot: P01375).
3. Claudin-4 (PA5-32354) was raised against a C-terminal synthetic peptide from the human Claudin-4 C-terminus. According to the supplier's documentation, it has been validated for use in immunohistochemistry and immunofluorescence on formalin-fixed paraffin-embedded tissues, with demonstrated specificity for Claudin-4.
4. E-cadherin (PA5-80457): This antibody is raised against recombinant human E-cadherin (Met1–Ile707) and the manufacturer's documentation shows it has been validated in IHC and IF applications.

Animals and other research organisms

Policy information about studies involving animals; ARRIVE guidelines recommended for reporting animal research, and Sex and Gender in Research

Laboratory animals	Yorkshire pigs (Sus scrofa domestica), 4–8 months old, sourced from the Cummings School of Veterinary Medicine at Tufts University (Grafton, MA, USA). These animals are outbred livestock animals; therefore, substrain and specific genetic background information are not applicable.
Wild animals	N/A
Reporting on sex	Only female pigs were available for use in the study, as they are generally more amenable to social housing and tend to be less aggressive than males. As such, the sex was not considered in the study design.
Field-collected samples	N/A
Ethics oversight	All procedures conformed to the protocols approved by the Massachusetts Institute of Technology Committee on Animal Care.

Note that full information on the approval of the study protocol must also be provided in the manuscript.

Plants

Seed stocks	N/A
Novel plant genotypes	N/A
Authentication	N.A
