# Supplementary material for: Oesophageal tissue screening system for assessing the retention and mucosal absorption of biologics
Source: Nat Biomed Eng. 2026 Jun 12;10(10):2213–29. doi: 10.1038/s41551-026-01685-9 (PMC13672101; doi:10.1038/s41551-026-01685-9)
Supplement: Supplementary file 3 [file 41551_2026_1685_MOESM3_ESM.pdf]

nature portfolio

Peer Review File

Oesophageal Tissue Screening system for Assessing the Retention and Mucosal Absorption of Biologics

Corresponding Author: Dr Giovanni Traverso

Version 0:

Decision Letter:

Dear Gio,

Thank you again for submitting to *Nature Biomedical Engineering* your manuscript, "Oesophageal Tissue Screening (OTiS) Platform", and please accept my apologies for the delay in getting back to you. The manuscript has been initially seen by 3 experts, but despite our efforts to receive all the reports, we are still waiting for the comments from Reviewer #3. Should we receive their comments, I will forward them to you. In the meantime, I recruited one additional expert and you can find the reports we received at the end of this message.

You will see that the reviewers appreciate the work. However, they express concerns about the technical reporting in support of the claims, and provide useful suggestions for improvement. We hope that with further work you can address the criticisms and convince the reviewers of the merits of the study. In particular, we would expect that a revised version of the manuscript provides:

* Thorough reporting of the physiochemical features considered in the machine learning model.
* Quantification of relevant data, and rigorous statistical analysis.

When you are ready to resubmit your manuscript, please upload the revised files, a point-by-point rebuttal to the comments from all reviewers, the reporting summary, and a cover letter that explains the main improvements included in the revision and responds to any points highlighted in this decision.

Please follow the following recommendations:

* Clearly highlight any amendments to the text and figures to help the reviewers and editors find and understand the changes (yet keep in mind that excessive marking can hinder readability).

* If you and your co-authors disagree with a criticism, provide the arguments to the reviewer (optionally, indicate the relevant points in the cover letter).

* If a criticism or suggestion is not addressed, please indicate so in the rebuttal to the reviewer comments and explain the reason(s).

* Consider including responses to any criticisms raised by more than one reviewer at the beginning of the rebuttal, in a section addressed to all reviewers.

* The rebuttal should include the reviewer comments in point-by-point format (please note that we provide all reviewers will the reports as they appear at the end of this message).

* Provide the rebuttal to the reviewer comments and the cover letter as separate files.

We expect that you will be able to resubmit the manuscript within 15 weeks of receiving this message. If this is the case, you will be protected against potential scooping. Otherwise, we will be happy to consider a revised manuscript as long as the significance of the work is not compromised by work published elsewhere or accepted for publication at *Nature Biomedical Engineering*.

We hope that you will find the referee reports helpful when revising the work. Please do not hesitate to contact me should you have any questions.

Best wishes,

Valeria

__
Dr Valeria Caprettini
Senior Editor, *Nature Biomedical Engineering*

____
Reviewer #1 (Report for the authors (Required)):

Although esophageal inflammatory diseases are global threats, oral delivery of biologics for the esophagus is an under-investigated field. Based on deep clinical understandings, the author integrated multidisciplinary engineering and biological tools and strategies to tackle such challenges, with a clear goal for clinical translation. The author first defined the delivery issues for esophagus delivery and then developed a high-throughput screening platform to identify various chemicals and their combinations for enhanced permeability of esophageal mucosa for macromolecules. Based on the mucoadhesive hydrogel formulations combined with the most effective permeation enhancers, the optimized macromolecular permeability was validated using various ex vivo esophagus tissues. Finally, the author assessed the formulation retention and mucosal uptake of infliximab in a pig model in a semi-orthostatic position. In general, both esophageal diseases and specific drug development for such conditions are less developed. The current study is timely, well-designed, and implemented, with great potential for further translational development. This review is optimistic for this study and believes the current study is worth a rapid publication after addressing the following issues.

1. The strength of the current study is on the engineering side and directly addresses the clinical needs for esophageal disease drug delivery; however, the data to support the underlying molecular mechanism for the enhancers and formulations are not convincing.
2. All studies were lasted for 4 hours. For long-term safety concerns, there is no data to support this. It is recommended to evaluate the low, medium, and high dosages for oral delivery formulations.
3. For the in vivo assessment of the pig model (figure 7), the therapeutic efficacy would be provided alongside direct comparison with clinical standard treatment. Only representative images for the histological study were provided. More samples and related statistical analysis should be provided.
4. The readability of Figure 4, 5 panel is relatively low. There are various types in the manuscript.
5. The title of the manuscript is recommended to reflect more about the research content instead of being a platform only, since various enhancers and formations have been identified and validated.

Reviewer #2 (Report for the authors (Required)):

In this manuscript, “Oesophageal Tissue Screening (OTiS) Platform” Karavasili et al describe a new method to investigate agents to improve the permeability of drugs in the esophagus, and they identified permeation enhancers and a hydrogel delivery platform to enable safe and effective permeation of the esophagous to enable the delivery of macromolecule drugs. The work conducts in-depth screening experiments using tissue models, computational assessments of the structural features of the enhancers, and effective delivery via in vivo experiments in a porcine model. The work clearly describes a useful method for discovery and results that have significant implications in the treatment of disease, including and beyond oeosinophilic esophagitis. Also exciting are the mechanistic studies on the permeation enhancement. This reviewer recommends publication upon addressing the following issues:

1. Supplementary Figures that are cited in the main text are missing in the Supplementary Information file. Please add these figures to the revised manuscript SI file.

2. Regarding the machine learning model, it is warranted to list all of the physiochemical features that were considered, and at least those in the top 40 most highly correlated features, and also to show the raw correlations of the physiochemical features to the experimental results. This information is important especially important because machine learning is notoriously a “black box.” The SHAP analysis was appreciated and important.

3. In Figure 6b, no negative control is apparently shown; one should be provided if valid in this type of comparison.

4. Some degree of image quantification is warranted, at least those crucial to the main claims of the paper, such as Figure 7 d + e.

5. The impact of peristaltic action in the esophagus was not discussed, regarding either its role in transporting the hydrogel down the esophagus or the potential for peristalsis to impart mechanical forces on the hydrogel. For the in vivo studies, it is not clear whether peristalsis would be occurring or inhibited/slowed in any way, and whether peristaltic forces would be imparted to the hydrogel during the experiment. Also, any differences regarding peristalsis between humans and pigs would

be important to note.

6. The work to determine the mechanism of the permeation enhancement (Pages 6-7) is important and exciting but does not seem sufficiently clearly discussed in the results section, and supporting evidence may be warranted. The work should preferably be separated out under another heading, and the experimental results should be carefully discussed in order of what is known so that the conclusions are clear, in terms of what is demonstrated experimentally and what is previously known. For instance, regarding the sentence “Transport of hydrophilic macromolecules across epithelia occurred mostly via the paracellular pathway, which is regulated and limited by the presence of intercellular junction proteins.” it is not clear whether this is a conclusion from the experiments in the paper or from a literature citation. The conclusion of increased paracellular transport seems likely, but additional experimentation such as in vitro imaging or transwell measurements, may better make this case.

Minor Comments:

1. Figure 6b: CDH and SDH should be defined in caption.

2. Papp (apparent permeability) should be defined in the main text instead of only in the Methods section.

3. It is not clear whether the Dropbox link describing complete details of all relationships for profit and not for profit for G.T. can be viewed anonymously since sign-in is required.

Reviewer #4 (Report for the authors (Required)):

The authors describe the development and validation of an Oesophageal Tissue Screening platform aimed at improving drug formulation and delivery for oesophageal disorders. Through ex vivo testing on porcine and human oesophageal tissues, the authors identified a binary mixture of bile salts capable of significantly increasing the permeability of large molecules (up to 876-fold for a 4 kDa model drug). They incorporated these permeation enhancers into a mucoadhesive hydrogel that remained on the tissue surface for extended periods, overcoming the natural clearance mechanisms of the oesophagus. In vivo validation in pigs (positioned semi-orthostatically) demonstrated enhanced mucosal uptake of infliximab (150 kDa), with no evidence of permanent tissue damage and reversible modulation of cell–cell junction proteins. Overall, the results indicate that this platform can be used to identify new strategies for local, non-invasive delivery of macromolecules to the oesophagus.

This platform represents a methodological innovation. Traditional Franz diffusion cells allow only a few permeability assessments at a time, whereas this platform enables substantially higher throughput and introduces gravity and salivary flow to replicate the anatomical constraints of the oesophagus more accurately. The hydrogel formulation also demonstrates tissue adhesion under physiologically relevant flow conditions, indicating significant performance gains over standard liquid suspensions and film-forming approaches. In addition, the study offers mechanistic insights into how bile salt excipients can temporarily modulate cell–cell junctions, advancing the mechanistic understanding of paracellular transport in oesophageal tissues.
By demonstrating in vivo retention and uptake of a clinically relevant biologic in large animal models, while no permanent mucosal damage or systemic absorption was observed, the study points toward genuine translational potential.

The work addresses a gap in oesophageal drug delivery. Research on targeted therapies for this particular segment of the GI tract has been relatively limited. At present, there are no effective marketed formulations specifically designed to deliver biologics to the oesophagus. Demonstrating that a hydrogel-based delivery system can markedly enhance local macromolecule uptake without permanent tissue disruption opens the door to novel, non-invasive therapies for conditions beyond eosinophilic oesophagitis, improving both patient acceptance and adherence. Beyond just treating oesophageal conditions, it could spur the design of targeted formulations for other sections of the gastrointestinal tract that require localized delivery.
I recommend minor revision after addressing the following comments:

1. The authors have performed a lot of experiments using a large number of excipients and should be credited for that. Please explain the rational for testing these excipients
2. Permeability studies were conducted at ambient temperature, the authors are invited to comment on the effect of temperature and how their results mimic the in vivo situation
3. The authors are invited to comment if the hydrogels could host small lipophilic APIs
4. For clarity, please spell out all abbreviations in full upon their first use, e.g. GI, EO (page 2), SHAP values (page 5), TJ, AJ (page 7)
5. Can you please confirm the animal sedation protocol?
6.Please provide Supplementary Figures 7 and 8 that seem to be missing from the Supplementary information file.
7.Please adjust Tables S1 and S2 to fit within the page margins.

Version 1:

Decision Letter:

Dear Gio,

Thank you for your revised manuscript, "Oesophageal Tissue Screening (OTiS) Platform". Having consulted with the original reviewers (whose comments you will find at the end of this message), I am pleased to write that we shall be happy to publish the manuscript in *Nature Biomedical Engineering*.

We will be performing detailed checks on your manuscript, and in due course will send you a checklist detailing our editorial and formatting requirements. You will need to follow these instructions before you upload the final manuscript files.

Please do not hesitate to contact me if you have any questions.

Best wishes,

Valeria

__
Dr Valeria Caprettini
Senior Editor, *Nature Biomedical Engineering*

____
Reviewer #1 (Report for the authors (Required)):

The authors have thoroughly addressed most, if not all, issues raised during the previous review. Congratulations for the authors generated a great work. This reviewer has no more comments.

Reviewer #2 (Report for the authors (Required)):

The authors have sufficiently addressed all outstanding comments and publication is recommended.

Reviewer #4 (Report for the authors (Required)):

The authors have adequately addressed my previous comments, and the manuscript has been substantially improved. I believe it is suitable for publication after minor revisions. Please consider the following additional points:
1. Supplementary Figure 12: Panels a–c appear to have image inserts that extend beyond the frame. Kindly correct the layout.
2. Figure S1: Please revise the description of the statistical analysis, as no individual data points are shown. This is understandable due to the data volume, but the text should clarify this appropriately.

Version 2:

Decision Letter:

Dear Gio,

I am happy to inform you that your manuscript, "Oesophageal Tissue Screening system for Assessing the Retention and Mucosal Absorption of Biologics", has now been accepted for publication in *Nature Biomedical Engineering*.

Over the next few weeks, the figures will be checked for production quality, the text edited to ensure that it conforms to house style, and the manuscript typeset.

Our Articles are published about 40 days after the acceptance date (we recommend that you inform your institutional press office of this timeframe), and you will be notified of the actual publication date a few days in advance. Articles can be

published any working day of the week, and are pushed live shortly after 10 am London time.

Publishing agreement. You will be asked to digitally sign a publishing agreement (grant of rights). After the signed publishing agreement has been received, the proofs of the article will be sent to you for review. If you have any queries during the production process, or you cannot meet the requested deadline for returning the proofs, please contact rjsproduction@springernature.com.

Nature Biomedical Engineering is a Transformative Journal. Authors may publish their research with us through the traditional subscription access route, or make their paper immediately open access through payment of an article-processing charge. More information about publication options is available.

You may need to take specific actions to comply with funder and institutional open-access mandates. If the work described in the accepted manuscript is supported by a funder that requires immediate open access (as outlined, for example, by Plan S) and your manuscript was originally submitted on or after January 1st 2021, then you should select the gold OA route. Authors selecting subscription publication will need to accept our standard licensing terms (including our self-archiving policies), and these will supersede any other terms that the author or any third party may assert apply to any version of the manuscript.

Acceptance of your manuscript is conditional on agreement, by all authors, with both our media embargo and confidentiality and pre-publicity policies. In particular, you may arrange your own publicity of the Article (for instance, through your institutional press office), as long as you ensure that journalists strictly adhere to the media embargo.

To assist you in disseminating the work, as soon as the Article is published you will be able to take advantage of the Springer Nature SharedIt initiative to generate a unique shareable link to the Article that will allow anyone (with or without a subscription) to read it. Recipients of the link who are subscribers will also be able to download and print the PDF.

Thank you for having submitted this work to *Nature Biomedical Engineering*.

Best wishes,

Valeria

__
Dr Valeria Caprettini
Senior Editor, *Nature Biomedical Engineering*

Open Access This Peer Review File is licensed under a Creative Commons Attribution 4.0 International License, which permits use, sharing, adaptation, distribution and reproduction in any medium or format, as long as you give appropriate credit to the original author(s) and the source, provide a link to the Creative Commons license, and indicate if changes were made.
In cases where reviewers are anonymous, credit should be given to 'Anonymous Referee' and the source.
The images or other third party material in this Peer Review File are included in the article's Creative Commons license, unless indicated otherwise in a credit line to the material. If material is not included in the article's Creative Commons license and your intended use is not permitted by statutory regulation or exceeds the permitted use, you will need to obtain permission directly from the copyright holder.
To view a copy of this license, visit https://creativecommons.org/licenses/by/4.0/

<u>Point-by-point response: *nBME-24-3013*</u>

We thank the reviewers and editorial team for the constructive comments. All changes to the main text and supplementary information are highlighted in yellow in the respective documents.

Response to Reviewer #1:
Report for the authors (Required):
Although esophageal inflammatory diseases are global threats, oral delivery of biologics for the esophagus is an under-investigated field. Based on deep clinical understandings, the author integrated multidisciplinary engineering and biological tools and strategies to tackle such challenges, with a clear goal for clinical translation. The author first defined the delivery issues for esophagus delivery and then developed a high-throughput screening platform to identify various chemicals and their combinations for enhanced permeability of esophageal mucosa for macromolecules. Based on the mucoadhesive hydrogel formulations combined with the most effective permeation enhancers, the optimized macromolecular permeability was validated using various ex vivo esophagus tissues. Finally, the author assessed the formulation retention and mucosal uptake of infliximab in a pig model in a semi-orthostatic position. In general, both esophageal diseases and specific drug development for such conditions are less developed. The current study is timely, well-designed, and implemented, with great potential for further translational development. This review is optimistic for this study and believes the current study is worth a rapid publication after addressing the following issues.

We appreciate the reviewer's positive feedback on our work.

1. The strength of the current study is on the engineering side and directly addresses the clinical needs for esophageal disease drug delivery; however, the data to support the underlying molecular mechanism for the enhancers and formulations are not convincing.

We thank the reviewer for this important comment. We respectfully submit that our study provides comprehensive mechanistic insight into the function of our oesophageal-targeted formulation through a multi-tiered approach that combines:

- Literature-supported mechanistic rationale
- High-throughput ex vivo screening
- Direct testing on both porcine and human tissues
- Histological and molecular analysis of tight junction protein modulation
- In vivo validation in a large animal model

Our goal at this development stage was to rigorously demonstrate both <u>functionality</u> and <u>mechanism of action</u> of the formulation in a physiologically relevant context.

1. Mechanistic Rationale and Experimental Design
We grounded our approach in established literature on paracellular transport, tight junction modulation, and calcium depletion-mediated loosening of epithelial barriers (e.g., Shen *et al.*, 2011; Ma *et al.*, 2000; Baruch *et al.*, 1991; Vllasaliu *et al.*, 2012). This informed the design of our experimental workflow (see cited references in the main text and methods).
Rather than relying on oversimplified monolayer models, which often fail to replicate the complex structure and dynamic environment of the oesophagus, we developed ex vivo models that incorporate key physiological parameters such as gravity and salivary clearance, enabling

simultaneous assessment of **adhesion**, **permeability**, **tissue integrity**, and **mechanism** in both porcine and human oesophageal tissues.

2. Robust Ex Vivo and Histological Evidence

We conducted extensive *ex vivo* permeability and mucoadhesion studies using custom-built horizontal and vertical plate systems (see Figs. 3, 5, 6; Fig. S1). These allowed us to identify a bile salt-based binary enhancer system that significantly increased permeability without compromising mucosal integrity.

To probe molecular mechanisms, we performed histological, immunohistochemical, and immunofluorescence analyses. We found that Oeso-gel treatment significantly reduced claudin 4 and E-cadherin expression, key markers of tight and adherens junctions, without histological signs of tissue damage (see Fig. 6d–e). These results directly support a paracellular transport mechanism.

Furthermore, we demonstrated that the permeation enhancers (bile salts) sequester Ca^{2+}, a mechanism known to transiently disrupt junctional complexes (see Fig. S5), aligning with previous findings on calcium depletion-mediated permeability enhancement.

3. In Vivo Confirmation of Mechanism and Safety

We validated our *ex vivo* findings in a swine model with semi-orthostatic positioning to replicate human esophageal anatomy (Fig. 7). *In vivo* data confirmed:

- Prolonged oesophageal retention of the hydrogel
- Enhanced mucosal uptake of infliximab
- Reversible modulation of junction proteins (E-cadherin and Claudin 4), with recovery of expression by day 3
- No observable mucosal toxicity

This level of in vivo mechanistic validation is, to our knowledge, unprecedented in the field of esophageal drug delivery.

4. Manuscript Revisions to Emphasize Mechanism

In response to this comment, we have revised the manuscript to better highlight mechanistic insights. A dedicated section titled *"Oeso-gel's effect on paracellular transport across tight junctions"* has been added (page 7), summarizing the histological, immunochemical, and calcium-depletion data and directly linking these findings to the observed permeability enhancements.

Added text (page 7):

> ***Oesogel's effect on paracellular transport across tight junctions***
>
> *Transport of hydrophilic macromolecules across epithelia occurs predominantly via the paracellular route, regulated by intercellular junction proteins (e.g., tight junctions [TJs] and adherens junctions [AJs]).[35] These structures act as selective barriers, limiting the diffusion of macromolecules and ions between cells. Previous studies have demonstrated that tight junction proteins, such as claudins, and adherens junction proteins, such as E-cadherin, are strongly associated with junction barrier function and are key regulators of epithelial integrity and permeability.[36]*
>
> *Building on this foundation, we investigated whether the significant increase in FD4 permeability seen upon treatment with Oesogel could be attributed to changes in the cellular distribution of these junction proteins. To this end, ex vivo human oesophageal tissues were analysed before (control) and after contact with the hydrogel formulation (with or without the PEs) using both immunohistochemistry **(Fig. 6d)** and*

*immunofluorescence (**Fig. 6e**). In control tissues, both proteins were localized on the cell membrane at the cell–cell contacts. Claudin 4 exhibited a strong membrane localization predominantly in the suprabasal layer, while E-cadherin staining was more prominent in the basal epithelial layer, consistent with prior findings on the stratified organization of oesophageal epithelia.[37 38] Upon treatment with the hydrogel (without PEs), a mild reduction in membrane-bound claudin 4 and E-cadherin was observed, with E-cadherin showing a more pronounced reduction. However, in tissues treated with the Oeso-gel, both claudin 4 and E-cadherin immunoreactivity were nearly undetectable by the end of the 4-hour permeability study. The same staining pattern was also observed after immunofluorescence staining of the human oesophageal tissue samples (**Fig. 6e**).*
*Interestingly, despite the apparent loss of junctional membrane staining, no evidence of cell detachment or widespread tissue damage was observed that would indicate protein integrity impairment as a result of the tissue treatment with the Oeso-gel, suggesting that the fundamental epithelial architecture remained intact. Given this lack of overt damage yet markedly enhanced permeability, we hypothesized that the Oesogel primarily affected the paracellular pathway rather than compromising overall tissue integrity. Calcium ions (Ca^{2+}) are vital for preserving TJ integrity and regulating paracellular transport.[39] Such Ca^{2+}-dependent modulation of TJs is well documented in the literature and has been shown to reversibly modulate the TJ barrier and increase paracellular permeability in epithelial models.[39 40] Bile salts have long been recognized for their Ca^{2+}-binding properties, effectively reducing the pool of free Ca^{2+} and further facilitating the loosening of TJ barriers.[35 41] Therefore, we hypothesized that the bile salts (PEs) present in Oesogel sequester free Ca^{2+}, leading to a conformational reorganization of junction complexes. Our data revealed a concentration-dependent decrease in free Ca^{2+} with increasing concentrations of CDC and SCH (PEs), culminating in nearly complete Ca^{2+} depletion at 25 mM CDC and 100 mM SCH (**Supplementary Fig. 5**).*
These data strongly suggest that calcium depletion in the local environment, triggered by the bile salts' high Ca^{2+} affinity, facilitates a temporary loosening of intercellular junctions. Such an effect aligns with the mechanism proposed by earlier investigations, in which chelating or reducing extracellular Ca^{2+} resulted in heightened macromolecular transport across epithelial barriers.[42]

We hope this response clarifies that our study not only addresses an important clinical need through engineering innovation, but also rigorously supports the molecular mechanism of action through multiple convergent lines of evidence across species and systems.

References:
Target Specific Tight Junction Modulators.
Adv Drug Deliv Rev 2021, 171, 266–288, Tight Junction Pore and Leak Pathways: A Dynamic Duo. Annual Review of Physiology 2011, 73 (Volume 73, 2011), 283–309. https://doi.org/10.1146/annurev-physiol-012110-142150,
Acid Modulates the Squamous Epithelial Barrier Function by Modulating the Localization of Claudins in the Superficial Layers. Lab Invest 2012, 92 (1), 22–31. https://doi.org/10.1038/labinvest.2011.139,
Dysregulation of Claudin-7 Leads to Loss of E-Cadherin Expression and the Increased Invasion of Esophageal Squamous Cell Carcinoma Cells. Am J Pathol 2007, 170 (2), 709–721. https://doi.org/10.2353/ajpath.2007.060343,
Calcium Regulation of Tight Junction Permeability. Ann N Y Acad Sci 2012, 1258, 93–99. https://doi.org/10.1111/j.1749-6632.2012.06539.x,

Mechanism of Extracellular Calcium Regulation of Intestinal Epithelial Tight Junction Permeability: Role of Cytoskeletal Involvement. Microsc Res Tech 2000, 51 (2), 156–168. https://doi.org/10.1002/1097-0029(20001015)51:2<156::AID-JEMT7>3.0.CO;2-J,
Calcium Binding to Bile Salts. Chem Phys Lipids 1991, 57 (1), 17–27. https://doi.org/10.1016/0009-3084(91)90045-d,
Evaluation of Calcium Depletion as a Strategy for Enhancement of Mucosal Absorption of Macromolecules. Biochem Biophys Res Commun 2012, 418 (1), 128–133. https://doi.org/10.1016/j.bbrc.2011.12.146)

2. All studies were lasted for 4 hours. For long-term safety concerns, there is no data to support this. It is recommended to evaluate the low, medium, and high dosages for oral delivery formulations.

We appreciate the reviewer's concern. Our *ex vivo* studies were conducted over 4–8 hours, consistent with the reported timeframe in the literature for maintaining oesophageal tissue viability and barrier integrity during permeability testing (AAPS PharmSciTech 2011; Arch Oral Biol 2005; Pharm Res 2005; J Control Release 2007; J Pharm Sci 2005).

In our *in vivo* experiments, we deliberately matched this timeframe to correlate with the *ex vivo* results, while also accommodating the logistical challenges of maintaining semi-orthostatic positioning in large pigs under anesthesia for extended periods.
Importantly, we monitored tissue integrity both acutely and 3 days post-administration, demonstrating through histological and immunohistochemical analyses that the tissue architecture and junctional protein expression (claudin 4, E-cadherin) remained intact and recovered after transient modulation (see Fig. 6, Fig. 7, Supplementary Figs. 7, 9–11).

We also demonstrated local infliximab uptake at a low dose (1 mg/mL, total dose 3 mL). This is noteworthy when compared to the standard IV infliximab dose (5–10 mg/kg) administered systemically every few weeks. While we agree that a comprehensive long-term safety and dose-response study (including low, medium, and high dosing) is warranted, we propose that these studies are best pursued after optimizing dosing frequency and establishing therapeutic efficacy through future clinical investigations.

References:

AAPS PharmSciTech. 2011 May 4;12(2):587–592. doi: 10.1208/s12249-011-9621-2
Arch Oral Biol. 2005 Dec;50(12):981-7. doi: 10.1016/j.archoralbio.2005.04.008
Pharm Res. 2005 Sep;22(9):1525-9. doi: 10.1007/s11095-005-6020-y
J Control Release. 2007 Sep 26;122(2):135-40. doi: 10.1016/j.jconrel.2007.05.017
J Pharm Sci. 2005 Dec;94(12):2777-88. doi: 10.1002/jps.20409

3. For the *in vivo* assessment of the pig model (figure 7), the therapeutic efficacy would be provided alongside direct comparison with clinical standard treatment. Only representative images for the histological study were provided. More samples and related statistical analysis should be provided.

We thank the reviewer for this insightful suggestion. Our current in vivo pig studies were explicitly designed as proof-of-concept experiments, with the aim of validating:

- Local mucosal uptake of infliximab,
- Formulation retention on the oesophageal surface,

- And the underlying mechanism of paracellular transport enhancement via tight junction modulation.

They were not intended to evaluate therapeutic efficacy, and as such, direct comparison with clinical treatment (e.g., IV Remicade) was not feasible at this stage.
Furthermore, as the reviewer correctly notes, there is currently no established large animal model that accurately recapitulates eosinophilic esophagitis, precluding rigorous disease-based efficacy assessments or direct benchmarking against standard clinical interventions.

That said, we fully agree that such comparisons will be essential for advancing this platform toward clinical translation, and we anticipate incorporating **head-to-head comparisons in future human trials**, where patient-relevant endpoints and validated clinical standards can be appropriately evaluated.

To address the reviewer's request for additional data, we have now:

- Included all available histological samples in the revised Supplementary Figures 9–11.
- Performed and reported statistical analyses across experimental timepoints and treatment groups (see manuscript pp. 8–9).

Summary of findings:

- E-cadherin and Claudin 4: A progressive decrease in E-cadherin staining was observed between 2–4 hours post Oeso-gel administration ($p > 0.05$), suggesting transient junction loosening. By day 3, junctional staining recovered, indicating the reversibility of Oeso-gel's action. Claudin 4 followed a similar pattern (Supplementary Fig. 7). Although inter-sample variability prevented statistical significance ($p > 0.05$), the trend was consistent across replicates.
- Infliximab uptake: Positive staining was detected in the basal oesophageal mucosa as early as 2 hours, with sustained presence at 4 hours and day 3 (Fig. 7d, Supplementary Fig. 11a). Notably, infliximab staining was significantly higher in the Oeso-gel group compared to hydrogel without permeation enhancers at 4 hours ($p < 0.01$). No systemic infliximab was detected in serum, confirming localized delivery.
- TNF-α modulation: Immunostaining revealed co-localization of TNF-α with infliximab uptake, with a statistically significant reduction in TNF-α staining at 2 hours in the Oeso-gel group ($p < 0.01$), possibly due to competitive binding. This provides indirect functional evidence of target engagement.

These updated data points collectively support the mechanistic action, mucosal retention, and functional tissue uptake of the formulation. While not a therapeutic study per se, the consistency of these findings provides a strong foundation for future efficacy studies in appropriate clinical settings.

The manuscript has been revised as follows [page 8-9]:

> *A progressive decrease in E-cadherin staining intensity was observed from 2 to 4 hours post-administration (p>0.05), consistent with a transient loosening of epithelial junctions. By day 3, staining intensity appeared to recover, suggesting reformation of cell–cell contacts and indicating the reversible nature of Oeso-gel's action (p>0.05). Although a trend was observed, the changes in staining intensity did not reach statistical significance (p > 0.05), likely reflecting inter-sample variability and suggesting the need for further validation in larger cohorts. The same effect was observed for Claudin 4* ***(Supplementary***

Fig. 7). *A similar staining pattern for E-cadherin was also noted on the biopsies collected from the animals to which the hydrogel (without PEs) was administered, albeit to a significantly lesser extent compared to the Oeso-gel treated group (t=4h: $p<0.01$).* *Positive immunohistochemical staining for infliximab was observed in the basal layers of the oesophageal mucosa as early as 2 hours following Oeso-gel administration* ***(Fig. 7d and Supplementary Fig. 11a)****. Staining intensity at 4 hours remained comparable to that at 2 hours, indicating sustained mucosal presence of the antibody over this interval. Retention of infliximab was also evident three days post-administration, suggesting prolonged mucosal residence. In contrast, the hydrogel-treated group (without PEs) showed no appreciable increase in staining across the experimental timeline ($p > 0.05$). Although not statistically significant ($p > 0.05$), the Oeso-gel group exhibited a consistent trend toward higher infliximab staining intensity compared to the hydrogel group, supporting the role of permeation enhancers in facilitating tissue uptake. No primary antibody controls were included to assess non-specific binding* ***(Supplementary Fig. 8)****. Immunolocalization of the cytokine revealed that TNF-α staining co-localized with infliximab uptake, and low levels of TNF-α tissue expression were detected in the oesophageal biopsies of both groups* ***(Fig. 7e and Supplementary Fig. 11b).*** *A trend toward reduced TNF-α staining was observed in the Oeso-gel group compared to the hydrogel group, reaching statistical significance at 2 hours post-administration ($p < 0.01$). This reduction may result from competitive binding of infliximab to TNF-α, limiting its detection by immunohistochemistry, a phenomenon previously reported in the context of anti-TNF therapies.[44,45] While indirect, this finding may support the notion of higher infliximab uptake in the Oeso-gel group relative to hydrogel alone. No detectable infliximab levels were quantified in the serum of the animals in either group.*

4. The readability of Figure 4, 5 panel is relatively low. There are various types in the manuscript.

We appreciate the reviewer's comment. The font size in figures 4 and 5 has been increased to improve readability.

5. The title of the manuscript is recommended to reflect more about the research content instead of being a platform only, since various enhancers and formations have been identified and validated.

We appreciate the reviewer's comment and have revised the title accordingly to: *'Oesophageal Tissue Screening (OTiS) Platform for Assessing the Retention and Mucosal Absorption of Biologics'*.

Response to Reviewer #2:

Report for the authors (Required): In this manuscript, "Oesophageal Tissue Screening (OTiS) Platform" Karavasili et al describe a new method to investigate agents to improve the permeability of drugs in the esophagus, and they identified permeation enhancers and a hydrogel delivery platform to enable safe and effective permeation of the esophagous to enable the delivery of macromolecule drugs. The work conducts in-depth screening experiments using tissue models, computational assessments of the structural features of the enhancers, and effective delivery via *in vivo* experiments in a porcine model. The work clearly describes a useful method for discovery and results that have significant implications in the treatment of disease, including and beyond oeosinophilic esophagitis. Also exciting are the mechanistic studies on the permeation enhancement. This reviewer recommends publication upon addressing the following issues:

1. Supplementary Figures that are cited in the main text are missing in the Supplementary Information file. Please add these figures to the revised manuscript SI file.

We thank the reviewer for their observation and apologize for the oversight. We have now included the missing Supplementary Figures 7 and 8 in the revised Supplementary Information file.

2. Regarding the machine learning model, it is warranted to list all of the physiochemical features that were considered, and at least those in the top 40 most highly correlated features, and also to show the raw correlations of the physiochemical features to the experimental results. This information is important especially important because machine learning is notoriously a "black box." The SHAP analysis was appreciated and important.

We thank the reviewer for their comment. A list of the top 40 most highly correlated physiochemical features along with their raw correlation values have been added in the Supporting Information file (Table S3).

3. In Figure 6b, no negative control is apparently shown; one should be provided if valid in this type of comparison.

We thank the reviewer for their comment. In this experiment, our goal was to screen and compare various hydrogel compositions in the presence of permeation enhancers (PEs) under conditions simulating the vertical orientation of the esophagus and salivary fluid flow. Including a hydrogel group without PEs would have produced negligible permeability across all tested hydrogels, making it impossible to discriminate effectively among the various compositions and thus identify the best performer. To address the question of PE-free conditions, we instead opted to show the best-performing hydrogel with and without PEs in Figure 6c, where the effect of PEs on permeability is clearly illustrated. As shown, the absence of PEs yielded negligible permeability, serving as the negative control for our most promising hydrogel formulation.

4. Some degree of image quantification is warranted, at least those crucial to the main claims of the paper, such as Figure 7 d + e.

We appreciate the reviewer's comment. Statistical analysis has been performed to all available histological samples (Supplementary Figures 9-11) and the manuscript has been revised as follows [page 8-9]:

> *A progressive decrease in E-cadherin staining intensity was observed from 2 to 4 hours post-administration ($p>0.05$), consistent with a transient loosening of epithelial junctions. By day 3, staining intensity appeared to recover, suggesting reformation of cell–cell contacts and indicating the reversible nature of Oeso-gel's action ($p>0.05$). Although a trend was observed, the changes in staining intensity did not reach statistical significance ($p > 0.05$), likely reflecting inter-sample variability and suggesting the need for further validation in larger cohorts. The same effect was observed for Claudin 4* ***(Supplementary Fig. 7)****. A similar staining pattern for E-cadherin was also noted on the biopsies collected from the animals to which the hydrogel (without PEs) was administered, albeit to a significantly lesser extent compared to the Oeso-gel treated group (t=4h: $p<0.01$).*
>
> *Positive immunohistochemical staining for infliximab was observed in the basal layers of the oesophageal mucosa as early as 2 hours following Oeso-gel administration* ***(Fig. 7d and Supplementary Fig. 11a)****. Staining intensity at 4 hours remained comparable to that*

at 2 hours, indicating sustained mucosal presence of the antibody over this interval. Retention of infliximab was also evident three days post-administration, suggesting prolonged mucosal residence. In contrast, the hydrogel-treated group (without PEs) showed no appreciable increase in staining across the experimental timeline ($p > 0.05$). Although not statistically significant ($p > 0.05$), the Oeso-gel group exhibited a consistent trend toward higher infliximab staining intensity compared to the hydrogel group, supporting the role of permeation enhancers in facilitating tissue uptake. No primary antibody controls were included to assess non-specific binding ***(Supplementary Fig. 8)****. Immunolocalization of the cytokine revealed that TNF-α staining co-localized with infliximab uptake, and low levels of TNF-α tissue expression were detected in the oesophageal biopsies of both groups* ***(Fig. 7e and Supplementary Fig. 11b).*** *A trend toward reduced TNF-α staining was observed in the Oeso-gel group compared to the hydrogel group, reaching statistical significance at 2 hours post-administration ($p < 0.01$). This reduction may result from competitive binding of infliximab to TNF-α, limiting its detection by immunohistochemistry, a phenomenon previously reported in the context of anti-TNF therapies.[44,45] While indirect, this finding may support the notion of higher infliximab uptake in the Oeso-gel group relative to hydrogel alone. No detectable infliximab levels were quantified in the serum of the animals in either group.*

5. The impact of peristaltic action in the esophagus was not discussed, regarding either its role in transporting the hydrogel down the esophagus or the potential for peristalsis to impart mechanical forces on the hydrogel. For the *in vivo* studies, it is not clear whether peristalsis would be occurring or inhibited/slowed in any way, and whether peristaltic forces would be imparted to the hydrogel during the experiment. Also, any differences regarding peristalsis between humans and pigs would be important to note.

We thank the reviewer for their valuable comment. The Conclusions section [page 10] has been revised as follows to address the reviewer's comment:

> *In our in vivo study, the animals were sedated throughout the experimental procedure, and it is well-documented that sedation can significantly affect oesophageal motor function, making it challenging to accurately account for normal peristaltic action.[59] Furthermore, anatomical and physiological distinctions between the porcine and human esophagus may further influence the mechanical forces acting on Oeso-gel in each species. Consequently, differences in peristaltic strength or coordination could affect how the hydrogel is transported and retained, meaning our in vivo findings in sedated pigs may not fully replicate clinical conditions in humans. It is therefore important to incorporate real-time imaging or manometric monitoring in future clinical evaluations to more accurately assess how active peristalsis affects hydrogel retention and transport under clinically relevant conditions.*

6. The work to determine the mechanism of the permeation enhancement (Pages 6-7) is important and exciting but does not seem sufficiently clearly discussed in the results section, and supporting evidence may be warranted. The work should preferably be separated out under another heading, and the experimental results should be carefully discussed in order of what is known so that the conclusions are clear, in terms of what is demonstrated experimentally and what is previously known. For instance, regarding the sentence "Transport of hydrophilic macromolecules across epithelia occurred mostly via the paracellular pathway, which is regulated and limited by the presence of intercellular junction proteins." it is not clear whether this is a conclusion from the experiments in the paper or from a literature citation. The conclusion of increased paracellular transport

seems likely, but additional experimentation such as *in vitro* imaging or transwell measurements, may better make this case.

We appreciate the reviewer's feedback and agree that separating the mechanistic discussion will help clarify our findings and the reasoning behind our conclusions. In response, we have now reorganized our manuscript and introduced a separate section in the results titled "Oesogel's effect on paracellular transport across tight junctions". We have also revised the relevant section in the manuscript to distinguish well-established principles from the literature from the new experimental evidence provided by our ex vivo and *in vivo* findings. The following text has been added in the revised manuscript [page 7]:

> ***Oesogel's effect on paracellular transport across tight junctions***
>
> *Transport of hydrophilic macromolecules across epithelia occurs predominantly via the paracellular route, regulated by intercellular junction proteins (e.g., tight junctions [TJs] and adherens junctions [AJs]).[35] These structures act as selective barriers, limiting the diffusion of macromolecules and ions between cells. Previous studies have demonstrated that tight junction proteins, such as claudins, and adherens junction proteins, such as E-cadherin, are strongly associated with junction barrier function and are key regulators of epithelial integrity and permeability.[36]*
>
> *Building on this foundation, we investigated whether the significant increase in FD4 permeability seen upon treatment with Oesogel could be attributed to changes in the cellular distribution of these junction proteins. To this end, ex vivo human oesophageal tissues were analysed before (control) and after contact with the hydrogel formulation (with or without the PEs) using both immunohistochemistry (**Fig. 6d**) and immunofluorescence (**Fig. 6e**). In control tissues, both proteins were localized on the cell membrane at the cell–cell contacts. Claudin 4 exhibited a strong membrane localization predominantly in the suprabasal layer, while E-cadherin staining was more prominent in the basal epithelial layer, consistent with prior findings on the stratified organization of oesophageal epithelia.[37 38] Upon treatment with the hydrogel (without PEs), a mild reduction in membrane-bound claudin 4 and E-cadherin was observed, with E-cadherin showing a more pronounced reduction. However, in tissues treated with the Oeso-gel, both claudin 4 and E-cadherin immunoreactivity were nearly undetectable by the end of the 4-hour permeability study. The same staining pattern was also observed after immunofluorescence staining of the human oesophageal tissue samples (**Fig. 6e**).*
>
> *Interestingly, despite the apparent loss of junctional membrane staining, no evidence of cell detachment or widespread tissue damage was observed that would indicate protein integrity impairment as a result of the tissue treatment with the Oeso-gel, suggesting that the fundamental epithelial architecture remained intact. Given this lack of overt damage yet markedly enhanced permeability, we hypothesized that the Oesogel primarily affected the paracellular pathway rather than compromising overall tissue integrity. Calcium ions (Ca^{2+}) are vital for preserving TJ integrity and regulating paracellular transport.[39] Such Ca^{2+}-dependent modulation of TJs is well documented in the literature and has been shown to reversibly modulate the TJ barrier and increase paracellular permeability in epithelial models.[39 40] Bile salts have long been recognized for their Ca^{2+}-binding properties, effectively reducing the pool of free Ca^{2+} and further facilitating the loosening of TJ barriers.[35 41] Therefore, we hypothesized that the bile salts (PEs) present in Oesogel sequester free Ca^{2+}, leading to a conformational reorganization of junction complexes. Our data revealed a concentration-dependent decrease in free Ca^{2+} with increasing concentrations of CDC and SCH (PEs), culminating in nearly complete Ca^{2+} depletion at 25 mM CDC and 100 mM SCH (**Supplementary Fig. 5**).*
>
> *These data strongly suggest that calcium depletion in the local environment, triggered by the bile salts' high Ca^{2+} affinity, facilitates a temporary loosening of intercellular junctions. Such an effect aligns with the mechanism proposed by earlier investigations,*

in which chelating or reducing extracellular Ca^{2+} resulted in heightened macromolecular transport across epithelial barriers.[42]

While additional *in vitro* experiments in relevant cell lines (e.g., Transwell permeability assays, live-cell imaging) could further clarify the precise molecular events underlying junction-protein modulation, our focus in this study was to evaluate Oeso-gel's performance under conditions closely mimicking clinical usage, where the three-dimensional architecture and multicellular environment of the oesophageal tissue are preserved. Importantly, we observed the same junction-protein modulation trend in *in vivo* oesophageal tissues harvested after topical application of Oeso-gel, reinforcing that these findings are not an artifact of the *ex vivo* setup. Together, our data extend the established knowledge on bile salt–induced junction-protein modulation by demonstrating a clear correlation between decreased junction-protein staining and enhanced macromolecular permeability in a physiologically relevant oesophageal model.

We anticipate that future work using more simplified *in vitro* systems could complement our findings by providing mechanistic details at the molecular or subcellular level, but we believe that the current approach offers direct translational relevance for oesophageal drug delivery. We appreciate the reviewer's suggestions and hope these revisions fully address the concerns regarding clarity and supporting evidence.

Minor Comments:

1. Figure 6b: CDH and SDH should be defined in caption.

We thank the reviewer for the careful review of our manuscript. We have updated the caption for Figure 6b to include the full definitions of CDH and SDH.

2. Papp (apparent permeability) should be defined in the main text instead of only in the Methods section.

We thank the reviewer for their observation. We have now provided the definition for Papp (apparent permeability) upon its first occurrence in the main text (Page 4-Section Overcoming the oesophageal mucosal barrier with a binary mixture of permeation enhancers).

3. It is not clear whether the Dropbox link describing complete details of all relationships for profit and not for profit for G.T. can be viewed anonymously since sign-in is required.

We have added a dated version to the revised manuscript.

Response to Reviewer #3:

None

Response to Reviewer #4:

Report for the authors (Required): **The authors describe the development and validation of an Oesophageal Tissue Screening platform aimed at improving drug formulation and delivery for oesophageal disorders. Through ex vivo testing on porcine and human oesophageal tissues, the authors identified a binary mixture of bile salts capable of significantly increasing the permeability of large molecules (up to 876-fold for a 4 kDa model drug). They incorporated these permeation enhancers into a mucoadhesive hydrogel that remained on the tissue surface for extended periods, overcoming the natural clearance mechanisms of the oesophagus. *In vivo* validation in pigs (positioned semi-orthostatically) demonstrated enhanced mucosal uptake of infliximab (150 kDa), with no evidence of permanent tissue damage and reversible modulation of cell–cell junction**

proteins. Overall, the results indicate that this platform can be used to identify new strategies for local, non-invasive delivery of macromolecules to the oesophagus.
This platform represents a methodological innovation. Traditional Franz diffusion cells allow only a few permeability assessments at a time, whereas this platform enables substantially higher throughput and introduces gravity and salivary flow to replicate the anatomical constraints of the oesophagus more accurately. The hydrogel formulation also demonstrates tissue adhesion under physiologically relevant flow conditions, indicating significant performance gains over standard liquid suspensions and film-forming approaches. In addition, the study offers mechanistic insights into how bile salt excipients can temporarily modulate cell–cell junctions, advancing the mechanistic understanding of paracellular transport in oesophageal tissues.
By demonstrating *in vivo* retention and uptake of a clinically relevant biologic in large animal models, while no permanent mucosal damage or systemic absorption was observed, the study points toward genuine translational potential.
The work addresses a gap in oesophageal drug delivery. Research on targeted therapies for this particular segment of the GI tract has been relatively limited. At present, there are no effective marketed formulations specifically designed to deliver biologics to the oesophagus. Demonstrating that a hydrogel-based delivery system can markedly enhance local macromolecule uptake without permanent tissue disruption opens the door to novel, non-invasive therapies for conditions beyond eosinophilic oesophagitis, improving both patient acceptance and adherence. Beyond just treating oesophageal conditions, it could spur the design of targeted formulations for other sections of the gastrointestinal tract that require localized delivery.
I recommend minor revision after addressing the following comments:

We thank the reviewer for their time and valuable feedback on our work.

1. The authors have performed a lot of experiments using a large number of excipients and should be credited for that. Please explain the rational for testing these excipients

We thank the reviewer for their comment. We have updated the manuscript as follows [Page 13]:

> *'These excipients were chosen to represent a diverse library of chemical permeation enhancers while still including conventional penetration enhancers frequently cited in the literature for use across the GI tract.'*

2. Permeability studies were conducted at ambient temperature, the authors are invited to comment on the effect of temperature and how their results mimic the *in vivo* situation

We appreciate the reviewer's valuable observation. We acknowledge that a thermostated setup for the vertical plates would more closely mimic *in vivo* conditions and could be a beneficial improvement in future iterations of our set up. However, our primary objective in these *ex vivo* studies was to compare and rank the performance of various formulations, serving as a screening tool to identify the most promising candidate for subsequent *in vivo* evaluation. While not physiologically temperature-matched, these experiments still provided useful comparative data for formulation selection.

3. The authors are invited to comment if the hydrogels could host small lipophilic APIs

We would like to thank the reviewer for raising this point. Our primary objective was to identify permeation enhancers specifically tailored for macromolecules, which follow different

absorption pathways than small lipophilic APIs. Consequently, the Oeso-gel as formulated may not be immediately applicable for small lipophilic drugs. However, the hydrogel platform itself (without the selected permeation enhancers) could still offer benefits for small lipophilic APIs by prolonging their retention on the oesophageal surface, potentially enhancing local absorption.

4. For clarity, please spell out all abbreviations in full upon their first use, e.g. GI, EO (page 2), SHAP values (page 5), TJ, AJ (page 7)

We thank the reviewer for their careful review of our manuscript. We have revised the text to ensure that all abbreviations are spelled out in full upon their first occurrence.

5. Can you please confirm the animal sedation protocol?

We thank the reviewer for their comment. We have updated the manuscript as follows page 16]:

> *'Animals were sedated with intramuscular injection of midazolam (~0.25 mg/kg) and dexmedetomidine (~0.03 mg/kg), and after intubation, anaesthesia was maintained with isoflurane at 2% with oxygen.'*

6.Please provide Supplementary Figures 7 and 8 that seem to be missing from the Supplementary information file.

We thank the reviewer for their observation and apologize for the oversight. We have now included the missing Supplementary Figures 7 and 8 in the revised Supplementary Information file.

7.Please adjust Tables S1 and S2 to fit within the page margins.

We thank the reviewer for highlighting this formatting issue. We have resized Tables S1 and S2 to ensure they fit within the page margins in the revised Supplementary Information file.
